# *N*-Methyl deuterated rhodamines for protein labelling in sensitive fluorescence microscopy†

**DOI:** 10.1039/d1sc06466e

**Published:** 2022-06-28

**Authors:** Kilian Roßmann, Kerem C. Akkaya, Pascal Poc, Corentin Charbonnier, Jenny Eichhorst, Hannes Gonschior, Abha Valavalkar, Nicolas Wendler, Thorben Cordes, Benjamin Dietzek-Ivanšić, Ben Jones, Martin Lehmann, Johannes Broichhagen

**Affiliations:** Leibniz-Forschungsinstitut für Molekulare Pharmakologie Berlin Germany broichhagen@fmp-berlin.de mlehmann@fmp-berlin.de; Section of Endocrinology and Investigative Medicine, Imperial College London London W12 0NN UK; Department of Chemical Biology, Max Planck Institute for Medical Research Heidelberg Germany; Leibniz Institute for Photonic Technology Jena e.V. (Leibniz-IPHT), Research Department Functional Interfaces Jena Germany; Physical and Synthetic Biology, Faculty of Biology, Ludwig-Maximilians-Universität München Großhaderner Str. 2-4, Planegg-Martinsried 82152 Germany

## Abstract

Rhodamine fluorophores are setting benchmarks in fluorescence microscopy. Herein, we report the deuterium (d12) congeners of tetramethyl(silicon)rhodamine, obtained by isotopic labelling of the four methyl groups, show improved photophysical parameters (*i.e.* brightness, lifetimes) and reduced chemical bleaching. We explore this finding for SNAP- and Halo-tag labelling in live cells, and highlight enhanced properties in several applications, such as fluorescence activated cell sorting, fluorescence lifetime microscopy, stimulated emission depletion nanoscopy and single-molecule Förster-resonance energy transfer. We finally extend this idea to other dye families and envision deuteration as a generalizable concept to improve existing and to develop new chemical biology probes.

## Abstract 2

Deuteration enhances photophysical and chemical properties of fluorescent rhodamine dyes for higher brightness in sensitive microscopy.

## Graphical abstract

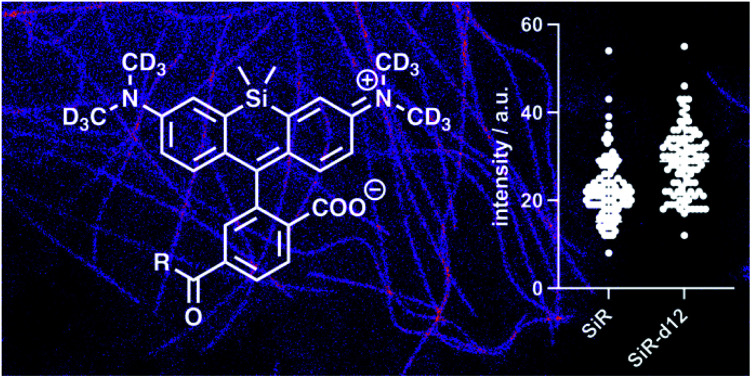

## Introduction

Fluorescence microscopy is the technique of choice in modern biomedical research to elucidate structures or to interrogate function. Efforts to improve performances resulted in the development of super-resolution microscopy (nanoscopy), which is defined to obtain resolution higher than the diffraction limit described by Abbe's law.^1–3^ While optics and instruments have been advanced constantly, the elaboration of synthetic molecular dyes has driven the field to the current state-of-the-art.^4^ Given their small size, and the possibility to target them to cellular organelles by linking them to molecular targeting scaffolds (*e.g.* ER-Tracker™ or MitoTracker™) or to self-labelling protein (*e.g.* SNAP/Halo-tag fusions) substrates, is making them an attractive choice for the visualization and interrogation of biomolecular function.^5,6^ Several chemical scaffolds, for instance nitrobenzodioxazoles (NBDs)^7^ and coumarins,^8^ remain interesting synthetic targets, yet xanthene dyes, which include tetramethylrhodamine (TMR) and silicon rhodamine (SiR), experienced a renaissance in terms of novel modifications to tune and boost important parameters, such as color, brightness, lifetime and reactivity.^9–13^ Isotopic labelling was recently among this,^14–16^ yet remained underexplored.

## Results

**Fig. 1:**
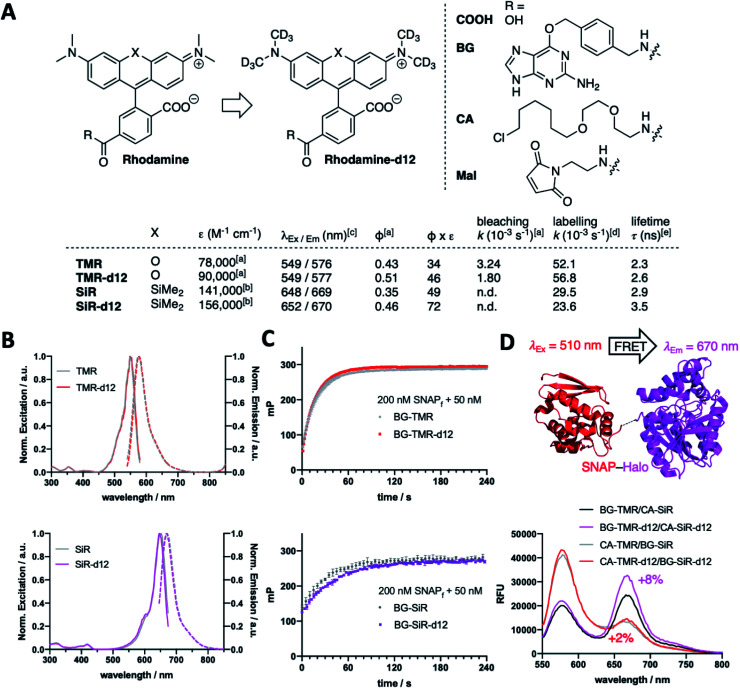
Deuteration improves tetramethylrhodamine brightness. (A) The isotopic deuterium incorporation on the four methyl groups leads to d12 variants of TMR (X = O) and SiR (X = SiMe_2_). Derivatization on the 6-carboxylate allows synthesis of BG-, CA- and Mal-congeners for SNAP-tag, Halo-tag and thiol labelling, respectively. (B) Excitation and emission spectra of TMR(-d12) (top) and SiR(-d12) (bottom). (C) Fluorescence polarization (mP) assay of BG-TMR(-d12) (top) and BG-SiR(-d12) (bottom) when incubated with SNAP_f_ to determine labelling kinetics. (D) *In vitro* FRET assay of a BG-TMR(-d12) and CA-SiR(-d12) labelled SNAP-Halo protein shows an increase of acceptor/donor emission ratio for deuterated compounds. *n* = 3 measurements. ^*a*^In PBS; ^*b*^in EtOH + 1% TFA; ^*c*^in activity buffer (containing in mM: NaCl 50, HEPES 50, pH 7.3); ^*d*^R = BG and SNAP_f_*in vitro* in activity buffer; ^*e*^SNAP bound in cells.

We fill the gap of isotopic labelling by designing, synthesizing, and testing carboxy-6-tetramethylrhodamines, in which each CH_3_ group is replaced by CD_3_ (Fig. 1A and Scheme S1†). Comparing deuterated and conventional rhodamines, we find that some photophysical properties remained unchanged, such as maximal excitation and emission wavelengths (*λ*_Ex/Em_(TMR(-d12)) ∼ 550/575 nm; *λ*_Ex/Em_(SiR(-d12)) ∼ 650/670 nm) (Fig. 1A and B), while others do change dramatically, such as extinction coefficients (*ε*(TMR)^9^ = 78 000 *vs. ε*(TMR-d12) = 90 000 M^−1^ cm^−1^; *ε*(SiR)^9^ = 141 000 *vs. ε*(SiR-d12) = 156 000 M^−1^ cm^−1^) and absolute quantum yields (*Φ*(TMR) = 0.43 *vs. Φ*(TMR-d12) = 0.51; *Φ*(SiR) = 0.35 *vs. Φ*(SiR-d12) = 0.46) leading to augmented brightness (*ε* × *Φ* × 10^−3^: TMR = 34 *vs.* TMR-d12 = 46; SiR = 49 *vs.* SiR-d12 = 72) (Fig. 1A and Table S1†). As silicon rhodamines are highly fluorogenic dyes, it is important to assess their maximal extinction coefficients for comparison in 1% TFA in EtOH,^9,13^ which on the flipside does not resemble a physiological solvent system to acquire for instance bleaching experiments. As such, we used TMR(d12) in buffer and subjected samples to strong white light irradiation and recorded fluorescence output after different time points. Indeed, we obtained slower bleaching rates for the deuterated compound (*k*(TMR) = 3.24 × 10^−3^*vs. k*(TMR-d12) = 1.80 × 10^−3^ s^−1^) with respect to the hydrogen-bearing molecule (Fig. 1A and ESI Fig. 1†). We furthermore equipped TMR-d12 and SiR-d12 with a bioorthogonal *O*^6^-benzylguanine (BG), a chloroalkane (CA) or a maleimide (Mal) linker handle on their 6-carboxy position for SNAP-, Halo-tag or thiol labelling, respectively. The *in vitro* labelling of SNAP with BG-TMR-d12 and BG-SiR-d12 could be traced by an increase in fluorescence polarization to determine labelling kinetics, which do not differ between hydrogenated and deuterated substrates (*k*(TMR) = 52.1 × 10^−3^*vs. k*(TMR-d12) = 56.8 × 10^−3^ s^−1^; *k*(SiR) = 29.5 × 10^−3^*vs. k*(SiR-d12) = 23.6 × 10^−3^ s^−1^) (Fig. 1A and C). By labelling a SNAP-Halo construct with CA-TMR/BG-SiR or BG-TMR/CA-SiR as donor/acceptor pair, the improved photophysical properties lead to an increased efficiency in Förster resonance energy transfer (FRET) by 2% and 8%, respectively, for the d12 variants (Fig. 1D). These results highlight that even subtle chemical changes can have pronounced effects on spectroscopic properties, exploring the chemical space in a new direction.

**Fig. 2:**
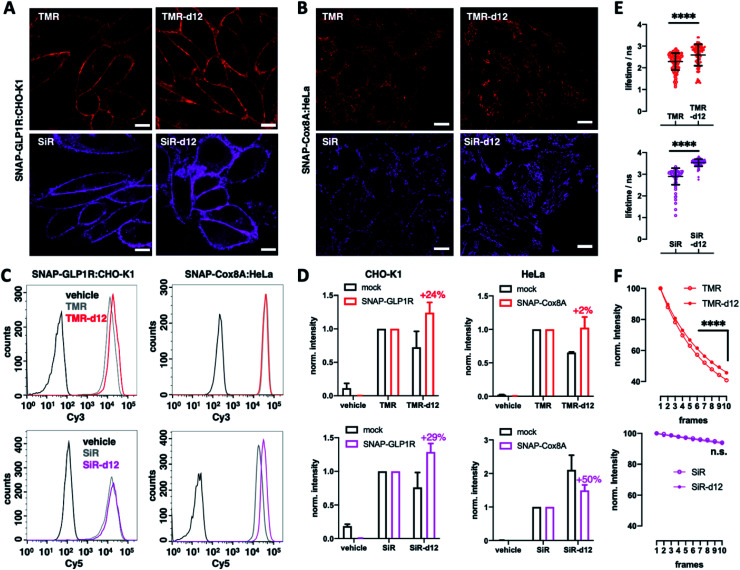
Microscopy and cell sorting reveal increased intensities of tetramethylrhodamines. (A) BG-TMR(-d12) (top) and BG-SiR(-d12) (bottom) label stable SNAP-GLP1R expressing CHO-K1 cells. Scale bar = 10 μm. (B) BG-TMR(-d12) (top) and BG-SiR(-d12) (bottom) label stable SNAP-Cox8A expressing HeLa cells. Scale bar = 10 μm. (C) Raw data from cell sorting of BG-TMR(-d12) (top) and BG-SiR(-d12) (bottom) labelled stable SNAP-GLP1R:CHO-K1 and SNAP-Cox8A:HeLa cells from (A) and (B). (D) As for C, normalized results including mock cells. (E) Fluorescence lifetimes of BG-TMR(-d12) (top) and BG-SiR(-d12) (bottom) labelled SNAP-GLP1R:CHO-K1 cells. Measurements from individual cells pooled from 2 independent experiments. Mean ± SD, **** indicates statistical significance (unpaired *t*-test, *p* < 0.0001). (F) As for (E) but bleaching curves. *n* = 3 independent experiments. Mean ± SD. **** indicates statistical significance (unpaired *t*-test, *p* < 10^−10^), n.s. = non-significant.

We next turned to SNAP labelling and imaging in live cells on targets that are expressed extracellular or intracellular to determine their tagging and permeability characteristics.^6^ First, we employed CHO-K1 cells stably expressing SNAP-tagged glucagon-like peptide 1 receptor (SNAP-GLP1R:CHO-K1),^17^ a cell line intensely used to study the physiology of this class B G protein-coupled receptor (GPCR), which is involved in glucose homeostasis and a drug target in diabetic patients,^18,19^ as a benchmark for d12 performances. As such, cells were labelled with 1 μm BG-TMR/SiR(-d12) for 30 min, before washing and live imaging by confocal microscopy, revealing staining of SNAP-GLP1R with all deuterated and parental dyes tested (Fig. 2A). Secondly, having established labelling on the outer plasma membrane, we investigated intracellular staining in live HeLa cells that stably express SNAP-tagged Cox8A (SNAP-Cox8A:HeLa) in the inner mitochondrial membrane (Fig. 2B), which has been used to study mitochondrial ultrastructures in live cells.^20,21^ As for SNAP-GLP1R, we observed clean labelling with all dyes, and for both colors with an observable increase in brightness for the d12 derivatives. With this enhanced performance in microscopy, we wanted to quantify brightness by fluorescence activated cell sorting (FACS) to obtain robust values over large sample sizes. Accordingly, we labelled SNAP-GLP1R:CHO-K1 and SNAP-Cox8A:HeLa cells with both, BG-TMR(-d12) and BG-SiR(-d12) to compare red and far-red color intensities by subsequent sorting (Fig. 2C). Histograms of labelled SNAP-GLP1R:CHO-K1 cells showed a right-shift in fluorescence intensity when dyes were deuterated (Fig. 2C, left). In line with this, labelled SNAP-Cox8A:HeLa cells exhibited a pronounced shift to higher intensities for SiR-d12 compared to its non-deuterated congener (Fig. 2C, right), while TMR(d12) only displayed a subtle change. By normalizing intensities and comparison, we calculate higher mean intensities for our deuterated dye versions (Fig. 2D). While no large increase was observed in SNAP-Cox8A:HeLa cells for TMR-d12 (2%), mean intensity was markedly increased in SNAP-GLP1R:CHO-K1 cells (24%). Furthermore, SiR-d12 outperformed SiR on SNAP-GLP1R and SNAP-Cox8A with an intensity increase of 29% and 50%, respectively. In addition, fluorescent lifetime confocal microscopy (FLIM) revealed longer fluorescent lifetimes for d12 congeners compared to their counterparts (*τ*(TMR) = 2.3 *vs. τ*(TMR-d12) = 2.6 ns; *τ*(SiR) = 2.9 *vs. τ*(SiR-d12) = 3.5 ns) (Fig. 2E and Table S1†). Accounting for a higher chemical stability, TMR-d12 was not as susceptible to bleaching as TMR, while SiR and SiR-d12 exhibited similar, and compared to TMR, more photostable trends of bleaching in this microscopic setup (Fig. 2F). Setting the stage for more imaging opportunities, we endowed our recently reported LUXendin651, a SiR-linked antagonistic peptide with high affinity and selectivity towards GLP1R,^22,23^ with the SiR-d12 congener *via* cysteine conjugation to Mal-SiR-d12 (ESI Fig. 2A and B†). While this peptide targets the orthosteric site of GLP1R (ESI Fig. 2C†), we observed higher signal intensities and longer lifetimes (ESI Fig. 2D, E and Table S2†) of LUXendin651-d12 in fixed SNAP-GLP1R:CHO-K1 cells when compared to its first generation LUXendin651. These results demonstrate that rhodamines with CD_3_ bearing amines are not only applicable to live cell imaging but outperform non-deuterated fluorophores, which is in line with our *in vitro* data of the unbound dyes.

**Fig. 3:**
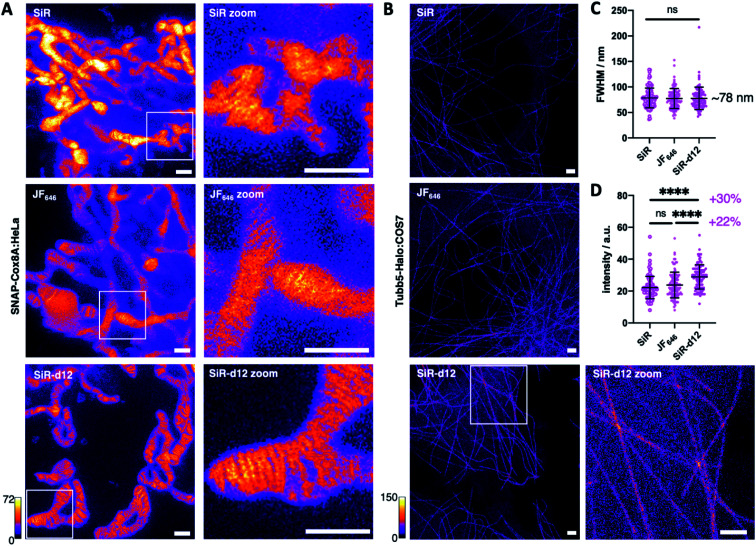
Deuteration improves tetramethylrhodamine performances and STED nanoscopy. (A) STED nanoscopy of BG-SiR (upper), BG-JF_646_ (middle) and BG-SiR-d12 (lower) in live SNAP-Cox8A:HeLa cells resolving mitochondrial cristae under identical imaging conditions. Scale bar = 1 μm (B) STED nanoscopy of CA-SiR (upper), CA-JF_646_ (middle) and CA-SiR-d12 (lower) in live Tubb5-Halo stably transfected COS7 cells resolving microtubule structures under identical imaging conditions. Scale bar = 1 μm. (C) FWHM of tubulin structures from (B), *n* = 100 line scans. n.s. = non-significant (unpaired *t*-test). Mean ± SD. (D) Intensity derived from tubulin structures from (B), *n* = 100. **** indicates statistical significance (unpaired *t*-test, *p* < 0.0001). Mean ± SD.

With these encouraging results, we decided to test our deuterated probes in stimulated emission by depletion (STED) microscopy, a state-of-the-art imaging technique to reveal cellular dynamics and structures.^4^ As such, and with SiR being one of the most successful far-red dyes for nanoscopy,^24^ we investigated super-resolution images acquired in live SNAP-Cox8A:HeLa cells and included JaneliaFluor646 (JF_646_) as an additional benchmark of dyes in the far-red regime. After incubation with 1 μm BG-SiR, BG-JF_646_ or BG-SiR-d12, we recorded images of mitochondrial cristae under the same conditions, and while all three dyes displayed labelling, SiR-d12 was able to resolve cristae sharper with less background (Fig. 3A). While this can have multiple reasons that may not only be attributed to dye performance, we targeted the cytoskeleton by labelling and imaging live Tubb5-Halo^25^ stably transfected COS7 cells (Tubb5-Halo:COS7) with homogenous expression levels (Fig. 3B). Microtubules resemble a classical benchmark to demonstrate the power of nanoscopy due to their constant diameter of ∼25 nm. After incubation with 1 μm CA-SiR, CA-JF_646_ or CA-SiR-d12, we observed microtubular fine structures with a full width half-maximum (FWHM) of ∼78 nm for all far-red dyes (Fig. 3C), notably with a marked increase in fluorescence intensity for SiR-d12 of 30% and 22% compared to SiR and JF_646_, respectively (Fig. 3D). Taken together, our deuterated d12 silicon rhodamine displayed augmented brightness in nanoscopic experiments while retaining resolution.

**Fig. 4:**
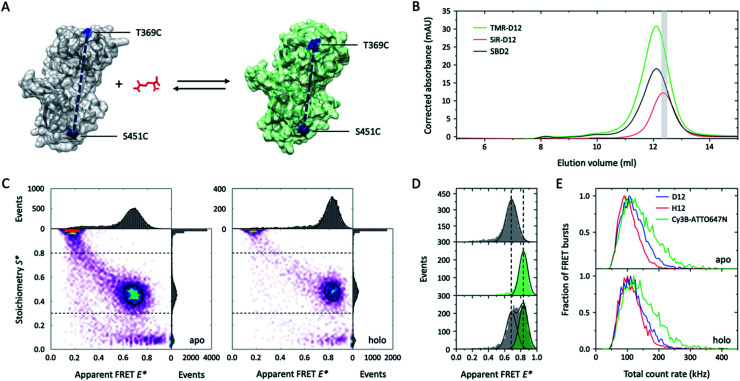
Deuteration improves tetramethylrhodamine performances in single-molecule FRET applications. (A) Crystal structure of GlnPQ-SBD2 in apo (grey, pdb file: 4KR5) and holo state (green, pdb file: 4KQP) with labeling positions indicated in blue. (B) Size-exclusion chromatography (SEC) was used to purify the protein after fluorophore labelling with maleimide-fused TMR–SiR(-d12) and Cy3B-ATTO647N.^31,32^ The degree of labelling could be determined *via* measurement of the protein absorption was measured at 280 nm (black curve), donor absorption (TMR-d12) at 540 nm, and acceptor dye absorption (SiR-d12) at 652 nm; molar concentrations were calculated based on published extinction coefficients.^31,32^ As indicated in grey, a fraction with high donor–acceptor yield was used for smFRET experiments. (C) E*–S* histograms obtained by μsALEX showing donor only (D-only, *S* > 0.8), acceptor only (A-only, *S* < 0.3), and species with both donor and acceptor fluorophore (DA, *S* > 0.3 and *S* < 0.8). Data shown here were recorded at excitation powers of 40 μW green and 15 μW red excitation power in imaging buffer without (apo) and with 100 μM glutamine (holo). (D) 1D-E* histograms of SBD2 in the absence (top), presence of 100 μM (middle) and 1 μM glutamine (low). (E) Comparison of the total count rate (sum of photon-count rates DA + DD + AA) of donor–acceptor labelled SBD2 proteins for TMR-d12–SiR-d12, TMR-SiR and Cy3B-ATTO647N in both holo and apo state reveals increased brightness of deuterated dyes. Additional data for all dyes and conditions is shown in ESI Fig. 3.†

Single-molecule FRET (smFRET)^26^ has become a well-established method to study (dynamic) conformational changes and heterogeneity of biomacromolecules.^27,28^ Alternating laser excitation^29^ (ALEX) describes one implementation of smFRET that allows the study of freely-diffusing molecules in solution at room temperature. Here, FRET efficiency is determined during short diffusional transits (on the timescale of milliseconds) of individual donor–acceptor-labelled molecules through the excitation volume of a confocal microscope. The technique allows observation of relative distance changes^29^ but also absolute distances^30^ with a spatial and temporal resolution limited by the available photon budget (count-rate).^27,28^ We thus tested whether higher count rates are available from deuterated fluorophores that are specifically attached to cysteine residues in proteins. Our test system was SBD2, the soluble extracellular substrate domain of the amino acid importer GlnPQ from *Lactococcus lactis*.^31,32^ SBD2 shows ligand-induced conformational changes between the ligand-free open and the ligand-bound closed state (Fig. 4A, apo *vs.* holo). The SBD2 variant has two label sites (T369C–S451C) that were stochastically labelled with the dye combinations TMR-d12–SiR-d12 (Fig. 4B and ESI Scheme 1†) and their non-deuterated form TMR–SiR, as well as Cy3B-ATTO647N as a control. For all dye combinations we achieved average labelling yields of ∼40–90% for each of the dyes; see ESI Fig. 3† for details.

The resulting E–S histograms of all different donor–acceptor pairs showed the expected donor-only (*S* > 0.8, D-only), acceptor-only (*S* < 0.3, A-only) and a donor–acceptor-containing species (*S* between 0.3–0.8, DA). The abundance of the DA population was on average >30% (ESI Fig. 3†), which generally facilitated fast data acquisition within ∼30 min. Analysis of the DA-species revealed a low FRET apo and high-FRET holo state (Fig. 4C), which is consistent with our previous investigations^31,32^ and the idea that the protein changes from the open to its closed state dependent on the glutamine concentration in the buffer (Fig. 4A). Also, the biochemical properties of SBD2 were preserved after fluorophore labelling indicated by equal population of both conformational states at a glutamine concentration close to the dissociation constant *K*_d_ of the protein of around 1 μM (Fig. 4D).

Further inspection of the data revealed differences in the photophysical properties of the dyes. While bleaching artefacts, *i.e.*, bridges between the three major populations, were almost absent for all pairs at the chosen laser powers of 40 μW green and 15 μW red excitation, the overall sum count-rate of donor-based donor-emission (DD), donor-based acceptor-emission (DA) and acceptor-based acceptor-emission (AA) was distinct for all dye combinations. In both apo and holo state Cy3B-ATTO647N was by far brighter in comparison to TMR–SiR with a significant number of molecules with count-rates >200 kHz (Fig. 4E). In agreement with results from Fig. 1–3, deuteration results in enhanced count-rates for TMR-d12–SiR-d12 over TMR–SiR. In summary, our results from smFRET investigations show that deuteration of rhodamines is a simple solution to improve spatial and temporal resolution in solution-based experiments *via* enhanced count rates. It was particular useful to see that both TMR–SiR (in deuterated and non-deuterated form) did not require addition of photostabilizers to the imaging buffer to obtain high-quality E–S histograms. Furthermore, the dye pair TMR–SiR has not been characterized and apparently shows a Förster radius similar to Cy3B-ATTO647N, which is larger than for the most commonly-used pairs in the field (*e.g.*, ∼5.0 nm for Alexa Fluor 555/647).^27,28^

**Fig. 5:**
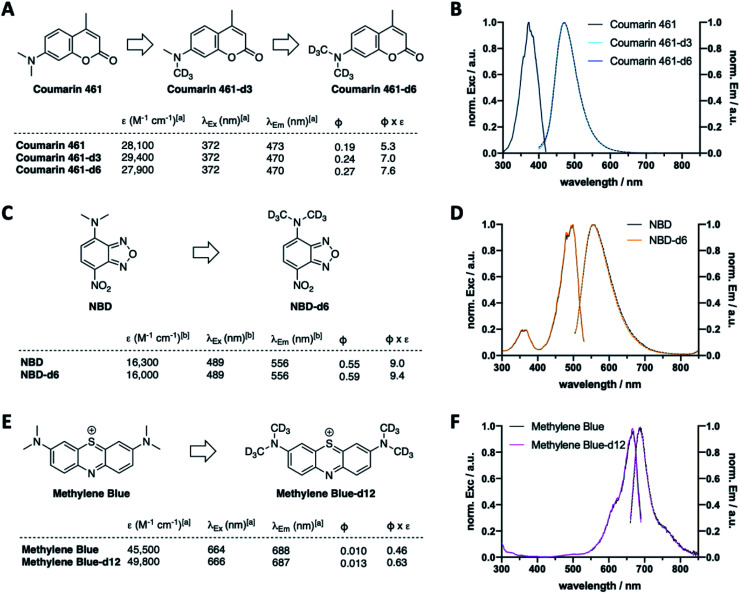
Deuteration improves fluorescent properties beyond tetramethylrhodamine dyes. (A) Deuterium installation on one or two methyl groups leads to d3 or d6 variants of Coumarin 461. (B) Excitation and emission spectra of Coumarin(-d3/6). (C) Deuterium installation on both methyl groups leads the d6 variant of NBD. (D) Excitation and emission spectra of NBD(-d6). (E) Deuterium installation on the methyl groups leads to d12 variant of methylene blue(-d12). (F) Excitation and emission spectra of methylene blue(-d12).

Following the results of deuterated rhodamines in various sensitive state-of-the-art fluorescence applications, we next asked if our deuteration approach is limited to rhodamine scaffolds or a general concept to enhance fluorescent dye properties. As such, we deuterated Coumarin 461 to obtain Coumarin 461-d3 and -d6 (Fig. 5A, Scheme S2A and Table S3†), which showed similar extinction coefficients (*ε*(Coumarin 461) = 28 100 *vs. ε*(Coumarin 461-d3) = 29 400 *vs. ε*(Coumarin 461-d6) = 27 900 M^−1^ cm^−1^) and similar maximal excitation and emission wavelengths (*λ*_Ex/Em_(Coumarin 461(-d3/6)) ∼ 372/470 nm) (Fig. 5B). As expected, quantum yield increased successively by deuteration level, giving rise to 43% higher brightness of Coumarin 461-d6 *vs.* Coumarin 461. Along these lines, CD_3_ installment on NBDs (Fig. 5C, D and Scheme S2B†) and methylene blue (Fig. 5E, F and Scheme S2C†) to give NBD-d6 and methylene blue-d12 followed the same trend: no change in excitation and emission wavelengths (Fig. 5D and F), yet brightness was enhanced in both cases by 4% and 37% for NBD and methylene blue, respectively, stemming from the product of extinction coefficient and quantum yield (*ε*(NDB) = 16 300 *vs. ε*(NBD-d6) = 16 000 M^−1^ cm^−1^; *ε*(methylene blue) = 45 500 *vs. ε*(methylene blue-d12) = 49 800 M^−1^ cm^−1^; *Φ*(NBD) = 0.55 *vs. Φ*(NBD-d12) = 0.59; *Φ*(methylene blue) = 0.010 *vs. Φ*(methylene blue-d12) = 0.013). This is encouraging towards the exploration of deuteration as a general approach to boost desired photophysical properties.

**Fig. 6:**
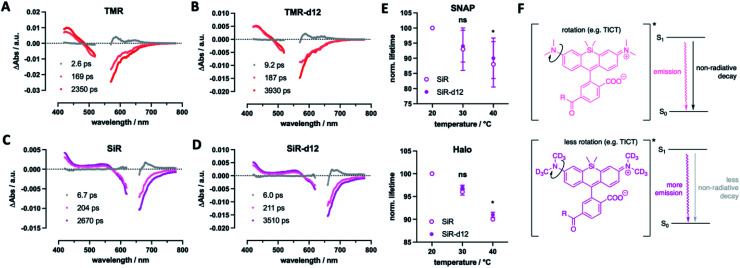
Mechanistic investigations for the deuteration effect. (A–D) Transient absorption spectroscopy for TMR (A), TMR-d12 (B), SiR (C) and SiR-d12 (D) is in agreement with fluorescent lifetimes that directly shows the absence of a dark and long-lived triplet state (E). Fluorescent lifetime was recorded in live COS7 cells stably transfected with Tubb5-SNAP:SiR(d12) (upper panel) and Tubb5-Halo:SiR(d12) (lower panel) in a temperature dependent manner with SiR-d12 showing significant higher lifetimes at elevated temperatures. (F) Mechanistic explanation of the deuteration effect by less non-radiative decay stemming from the suppressed twisted intramolecular charge transfer (TICT) and rotation around the dimethyl amino groups in SiR-d12 leads to higher lifetimes and quantum yields. *n* = 3 independent experiments derived from each 50–70 cells. * indicates statistical significance (unpaired *t*-test, *p* < 0.05). Mean ± SD.

Finally, we were curious to find some mechanistic insights of how deuterium incorporation improves fluorescent rhodamines. Firstly, we determined excited-state lifetimes for TMR(-d12) and SiR(-d12) by transient absorption spectroscopy, which agree with the lifetimes obtained by fluorescence lifetime imaging (Fig. 6A–D). This indicates that the decay of the luminescent state correlates with recovery of the electronic ground state. Particularly, the decay of the emissive state does not yield a long-lived triplet state, which would appear in a long-lived transient absorption signature. This led us to further investigate fluorescence lifetime, which depends on the rotation of the alkyl amine group, and as such is temperature sensitive, decreasing at elevated temperatures.^33^ If deuteration of the methyl groups affects rotational movements, we would be able to observe smaller changes in lifetime at increasing temperatures. For this reason, we acquired fluorescent lifetimes of SiR and SiR-d12 bound to Tubb5-SNAP and Tubb5-Halo-tags (Fig. 6E) in a temperature dependent manner by FLIM (Tables S4 and S5†). Indeed, when comparing lifetimes at 20, 30 and 40 °C, we found that both Tubb5-SNAP:SiR-d12 and Tubb5-Halo:SiR-d12 retained significant longer lifetimes at 40 °C when normalized to lifetimes at 20 °C. We therefore reason that non-radiative decay of the excited singlet state *via* rotation around the dimethyl amino group is suppressed due to the stronger and heavier nature of deuterium (Fig. 6F).

## Discussion

In our study, we synthesized and tested deuterated fluorophores with enhanced fluorescent properties in a set of applications. The use of deuterium to improve fluorescence emission properties has been addressed in the past by using deuterated solvents.^34^ In a preprint^14^ uploaded at the same time to our preprint,^15^ the Lavis laboratory reported on a similar approach, supporting our findings: by equipping the rhodamine nitrogen atoms with deuterated ring systems, they describe Halo-tag substrates with enhanced fluorescent properties (*e.g.* brightness and single particle tracing) for protein labelling in live cells.^16^ In this work, we install labelling moieties for SNAP- and Halo-tag conjugation and apply them in different experimental setups. This led to the finding that our TMR-d12 and SiR-d12 improve by swapping hydrogen with deuterium on the methyl groups, enhancing photophysical parameters, such as brightness and lifetime, while reducing critical chemical parameters, such as bleaching. We employed quantitative ^1^H NMR to assess concentrations of dyes in solution to precisely determine extinction coefficients, and measured quantum yields directly by use of an integrating sphere, and found in both systems significant higher values for d12 fluorophores.

While more mechanistic reasons for the enhanced properties may exist, we argue the following: (i) affecting the rotation around the aromatic carbon–nitrogen bond (in our case due to higher mass of the CD_3_ groups) has marked effects on fluorescent properties,^35^ which could suppress non-radiative decays and in turn enhances quantum yield and lifetime;^9^ (ii) a lower zero-point energy of the C–D *vs.* C–H bond results in slower reaction kinetics, as an higher energy barrier has to be overcome,^36^ and this would reduce bleaching through for example generated reactive oxygen species. This is backed up by the Lavis laboratory,^16^ since (i) it was observed that deuteration of azetidines (which are rotationally more locked)^9^ does not lead to a large increase in quantum yield, and (ii) light-induced demethylation of deuterated fluorophores is slower when compared to their non-deuterated congeners. Both of these arguments describe lower quantum yields of non-radiative decays, and result in an improved quantum yield for emission. In particular, the phenomenon in question is twisted intramolecular charge transfer (TICT), which is known to be temperature-dependent,^37^ and has been explored in many ways to enhance quantum yield of fluorophores by introducing steric demand and/or donor engineering.^38^ This is further supported by the Lavis lab, as it was shown that deuteration alters quantum yield on dyes depending on the TICT donor/acceptor.^16^ More experimentation is needed, ideally in combination with *in silico* calculations, that, for instance, have been performed on dyes under acidic H/D exchange^39^ where “a close examination of the low-lying singlet and triplet electronic states along the torsional motion of the amino groups revealed that the key to the isotope effect is changes in non-radiative channels”.^39^ As such, tunneling rates and intersystem crossing differences may contribute to our observed changes in fluorophore behavior. Keeping this in mind, we showcase deuterated dyes that outperform their parent molecules in multiple experiments.

The enhancements are significant and broadly applicable, ranging from *in vitro* FRET, to live cellular labelling and sorting, lifetime and super-resolution microscopy on SNAP- and Halo-tags and smFRET using maleimide-thiol chemistry. We observed “sharper” imaging on SNAP-Cox8A:HeLa cells with SiR-d12 compared to JF_646_ and SiR. As these experiments were performed in live cells, the subjective perception of enhanced imaging may be attributed to different expression levels, more or less healthy mitochondria, cell cycle phase, and the dynamic change in cristae thickness. Although our observations were consistent in three independent experiments, it remains difficult to quantify, and as such, we aimed to include an unambiguous experiment to determine the STED performance of SiR-d12. For this reason, we chose live Tubb5-Halo:COS7 cells, where the distribution of Halo-tagged microtubules is homogeneous and the diameter of the fine-structure is constant at 25 nm. Indeed, in this setup, we found outstanding performance of deuterated Halo:SiR-d12 compared to JF_646_ and SiR with a marked increase in brightness and no significant difference in resolution. In times where photon counts in sophisticated imaging experiments (*e.g.* MINFLUX^40^) are becoming increasingly more important, we anticipate that fluorophore deuteration provides a method to advance in the field as was shown by applications of deuterated dyes in single molecule experiments.

Furthermore, the concept was expandable to other dye scaffolds, such as coumarins, NBDs, and the thiazene containing dye methylene blue, giving enhanced brightness for all deuterated species. It should be noted here that extinction coefficients are within a close margin, however, quantum yields are significantly increased for all dyes tested.

We anticipate this concept (i) to be generalizable to other xanthenes (*e.g.* SNARFs, and quenchers like QSY7) at N–C_α_–H positions for improving and fine-tuning spectroscopic properties; (ii) to be further explored with other isotopes, such as ^13^C, ^15^N or even ^3^H that can be used as an additional, orthogonal radioactive tracer; (iii) to be used in different labelling approaches, such as the attachment to sulfonated BG (SBG) scaffolds allowing the separation of SNAP-tagged receptor pools,^41^ to biomolecule targeting probes,^42–44^ to “click chemistry” reagents (*e.g.* cyclopropenes, cyclooctenes)^45^ or to photoswitchable ligands,^46,47^ and (iv) to serve as multimodal dyes for isotope labelled mass spectrometric analysis, correlative light-electron microscopy (CLEM)^48^ and confocal Raman microscopy.^49,50^ Such efforts are of ongoing interest in our laboratories.

## Methods

### Synthesis

Chemical synthesis (ESI Schemes 1 and 2†) and characterization of compounds is outlined in the ESI.† Purity of all dyes was determined to be of >95% by UPLC-UV/Vis traces at 254 nm and dye specific *λ*_max_ that were recorded on a Waters H-class instrument equipped with a quaternary solvent manager, a Waters autosampler, a Waters TUV detector and a Waters Acquity QDa detector with an Acquity UPLC BEH C18 1.7 μm, 2.1 × 50 mm RP column (Waters Corp., USA).

### Extinction coefficients, excitation and emission profiles and quantum yield

To assess photophysical parameters, ^1^H NMR spectra of TMR(d12) and SiR(d12) were recorded with an internal standard (DMF) to determine concentrations. The same solutions were diluted 1 : 1000 in activity buffer (containing in mM: NaCl 50, HEPES 50, pH 7.3) or EtOH + 1% TFA, and absorbance spectra were acquired on a NanodropOne 2000C using a 1 cm Hellma Quartz cuvette. Extinction coefficients of d12 dyes were then calculated referenced to literature values of their non-deuterated parental molecules according to eqn (1).

1 *ε*_deuterated_ = *ε*_ref_ × ((Abs_deuterated_/Abs_ref_) × (*c*_ref_/*c*_deuterated_))

1 : 100 dilutions in activity buffer were transferred into Greiner black flat bottom 96 well plates and excitation and emission profiles were recorded on a TECAN INFINITE M PLEX plate reader (TMR: *λ*_Ex_ = 505 ± 10 nm; *λ*_Em_ = 550–800 ± 20 nm; 10 flashes; 20 μs integration time; SiR: *λ*_Ex_ = 605 ± 10 nm; *λ*_Em_ = 640–800 ± 20 nm; 10 flashes; 20 μs integration time; Coumarin: *λ*_Ex_ = 360 ± 10 nm; *λ*_Em_ = 400–750 ± 20 nm; 10 flashes; 40 μs integration time; NBD: *λ*_Ex_ = 440 ± 10 nm; *λ*_Em_ = 480–750 ± 20 nm; 10 flashes; 40 μs integration time; methylene blue: *λ*_Ex_ = 630 ± 10 nm; *λ*_Em_ = 660–850 ± 20 nm; 10 flashes; 40 μs integration time). Absorbance values and integrated emission area (AUC) was used to calculate quantum yield (QY) according to eqn (2) under the assumption that there is no change in refractive indices between solutions (for Coumarin 461-d3/6, NBD-d6 and methylene blue-d12):

2 QY_deuterated_ = QY_ref_ × ((Abs_ref_/Abs_deuterated_) × (AUC_deuterated_/AUC_ref_))

Experiments were run in quadruplicate. Data normalization, integration and plotting was performed in GraphPad Prism 8.

Absolute quantum yields were determined for TMR(-d12) and SiR(-d12) by first measuring steady-state UV-vis absorption spectroscopy on a Jasco V780 spectrophotometer and a Specord S600 (Analytik Jena) in 1 cm cuvettes. Steady-state emission spectroscopy: emission spectra were measured on an Edinburgh FLS980 emission spectrofluorometer in a 1 cm cuvette at 90° angle. The solutions were prepared to have an absorbance of 0.1 at 510 nm for TMR and at 600 nm for SiR in PBS and quantum yields were determined on the same instrument equipped with an integrating sphere.

### *In vitro* photobleaching

Solutions (20 μM) of TMR and TMR-d12 were prepared in activity buffer (containing in mM: NaCl 50, HEPES 50, pH 7.3 + 0.1% BSA). An aliquot of 10 μL was transferred into a 1.5 mL Eppendorf vial and spun down to form an homogenous aqueous drop at the bottom of the plastic tube. An aliquot was exposed to a white light beam in order to bleach the fluorophore with an Hg (Xe) arc lamp (LOT-QuantumDesign GmbH, Darmstadt, Germany) for 0, 1, 2, 3, 4, or 5 minutes. After bleaching, each aliquot was diluted by addition of 90 μL activity buffer + 0.1% BSA and carefully mixed by pipetting up and down, and 50 μL of this solution were transferred into a 10 × 3 mm black quartz cuvette with a side window (Hellma, Jena, Germany). Fluorescence spectra were recorded with a Jasco spectrofluorometer (FP-6500) at 25 °C from 300–750 nm with an excitation wavelength of 544 nm over 1 nm steps (BW (Ex) 5 nm, BW (Em) 1 nm, PMT 475 V). The experiment was performed in triplicate for each illumination time, the data points averaged and plotted *versus* time.

### SNAP_f_ and SNAP–Halo expression and purification

SNAP_f_ was expressed and purified as described previously^16^ and complete amino acid sequences for constructs used can be found in the ESI.† SNAP-Halo with an N-terminal Strep-tag and C-terminal 10xHis-tag was cloned into a pET51b(+) expression vector for bacterial expression and complete amino acid sequences for constructs used can be found in the ESI.† For purification, SNAP-Halo was expressed in the *E. coli* strain BL21 pLysS. LB media contained ampicillin (100 μg mL^−1^) for protein expression. A culture was grown at 37 °C until an OD_600_ of 0.6 was reached at which point cells were induced with IPTG (0.5 mM). Protein constructs were expressed overnight at 16 °C. Cells were harvested by centrifugation and sonicated to produce cell lysates. The lysate was cleared by centrifugation and purified by Ni-NTA resin (Thermofisher) and Strep-Tactin II resin (IBA) according to the manufacturer's protocols. Purified protein samples were aliquoted in activity buffer (containing in mM: NaCl 50, HEPES 50, pH 7.3), flash frozen and stored at −80 °C.

### SNAP_f_ labelling kinetics

Kinetic measurements were performed on a TECAN Spark Cyto and on a TECAN GENios Pro plate reader by means of fluorescence polarization. Stocks of SNAP_f_ (400 nm) and substrates (100 nm) were prepared in activity buffer (containing in mM: NaCl 50, HEPES 50, pH 7.3) with additional 10 μg mL^−1^ BSA. SNAP_f_ and substrates were mixed (100 μL each) in a Greiner black flat bottom 96 well plate. Mixing was performed *via* a built-in injector on a TECAN GENios Pro or by manual pipetting on a TECAN Spark Cyto for TMR and SiR substrates, respectively. Fluorescence polarization reading was started immediately (TMR: *λ*_Ex_ = 535 ± 25 nm; *λ*_Em_ = 590 ± 35 nm; 10 flashes; 40 μs integration time; SiR: *λ*_Ex_ = 605 ± 20 nm; *λ*_Em_ = 670 ± 20 nm; 10 flashes; 40 μs integration time). Experiments were run in five repetitions and raw polarization values were one-phase decay fitted in GraphPad Prism 8.

### Protein labelling for FRET and full protein mass spectrometry

For protein labelling, 1 μL of the corresponding dye(s) (200 μm in DMSO) were diluted in 220 μL of a 227 nm solution of SNAP-Halo in activity buffer (10 μg mL^−1^ BSA was added to controls where no SNAP-Halo protein was present). This resulted in a ∼4-fold excess of labelling substrate and mixing was ensured by carefully pipetting the solution up and down. The reaction mixture was allowed to incubate at r.t. for 1 h before 20 μL were removed for QToF MS analysis to ensure full labelling. The remaining solutions were subjected to spin column purification (Sartorius Vivaspin 500 30 kDa MWCO PES, #VS0122) for three times by adding 500 μL of activity buffer for each cycle. Finally, the solutions were reconstituted in activity buffer, of which 200 μL were transferred into a Greiner black flat bottom 96 well plate and emission spectra were recorded on a TECAN INFINITE M PLEX (TMR: *λ*_Ex_ = 510 ± 10 nm; *λ*_Em_ = 550–800 ± 20 nm; 25 flashes; 20 μs integration time; SiR: *λ*_Ex_ = 610 ± 10 nm; *λ*_Em_ = 650–800 ± 20 nm; 10 flashes; 20 μs integration time) to observe FRET. Donor or acceptor only labelled constructs, and donor plus acceptor with the addition of 10 μg mL^−1^ BSA served as controls, and in these cases acceptor emission was not observed. FRET efficiency was calculated from the sum of maximal emission values derived from the raw spectra *I*_TMR_ (572–584 nm) and *I*_SiR_ (660–672 nm) according to eqn (3):

3 *E*_FRET_ = *I*_SiR_/(*I*_TMR_ + *I*_SiR_)

### Cell culture and FACS of SNAP-GLP1R:CHO-K1 and SNAP-Cox8A:HeLa cells

CHO-K1 cells stably expressing the human SNAP-GLP1R (Cisbio) (SNAP-GLP1R:CHO-K1) were maintained in DMEM supplemented with 10% FCS, 1% penicillin/streptomycin, 500 μg mL^−1^ G418, 25 mM HEPES, 1% nonessential amino acids and 2% l-glutamine. HeLa cells stably expressing SNAP-Cox8A (ref. 20) (Cox8A-SNAP:HeLa) were maintained in DMEM supplemented with 10% FCS, 1% penicillin/streptomycin. Cells were incubated with 1 μm of BG-conjugated dye or with 500 nm LUXendin651(-d12) and 5 μm of Hoechst33342 for 30 min at 37 °C, 5% CO_2_. For FACS, cells were washed with PBS w/o Ca^2+^ and Mg^2+^ and detached using Trypsin/EDTA. Detached cells were pelleted at 300 g at 4 °C, resuspended in DPBS w/o Ca^2+^/Mg^2+^ and kept on ice until fluorescence activated cell sorting (FACS). FACS was performed in LSR-Fortessa (BD Bioscience) on cells gated by SSC/FSC using 561 nm excitation and BP 586/18 (561–586/18 BD channel) for TMR(-d12) or 640 nm excitation and BP 670/30 (640–670/30 BD channel) for SiR(d12). Mean fluorescence intensities of >10 000 cells were analyzed and measured using Flow Jo (BD Bioscience). Fixation for LUXendin651(-d12) widefield microscopy was performed using 4% paraformaldehyde for 20 minutes before washing once with PBS, quenched with a solution of 0.1 M glycine and 0.1 M NH_4_Cl in PBS for 10 min and imaged in PBS.

### Confocal, FLIM, widefield and super-resolution microscopy

Live cell fluorescence lifetime and confocal microscopy on stable SNAP-GLP1R:CHO-K1 or live STED imaging of COS7 cells transfected with Halo-Tubb5 (COS7:Halo-Tubb5) (Addgene plasmid #64691, gift from Yasushi Okada) was performed using a Leica SP8 TCS STED FALCON (Leica Microsystems) equipped with a pulsed white-light excitation laser (80 MHz repetition rate, NKT Photonics), a 100× objective (HC PL APO CS2 100×/1.40 NA oil), a temperature controlled chamber and operated by LAS X. TMR(-d12) were excited using *λ* = 561 nm and emission signals were captured at *λ* = 576–670 nm. SiR(-d12) were excited using *λ* = 640 nm and emission signals were captured at *λ* = 655–748 nm. The confocal images were collected using a time gated Hybrid detector (0.5–6 ns). FLIM images of sufficient signal were acquired without gating on Hybrid detectors within 512 × 512 pxl of 114 nm/pxl with 10 repetitions. Fluorescence lifetime decay curves from selected regions with clear plasma membrane staining were fitted with two exponential functions and the mean amplitude weighted lifetime is reported for each region.

Confocal fluorescence microscopy for photobleaching experiments were performed on living cells expressing SNAP-GLP1R in PBS at room temperature on LSM710 or LSM780 (Carl Zeiss) operated by Zen Black Software using a 63× (1.40 NA oil) objective. TMR(d12) were excited using *λ* = 561 nm and emission signals were captured at *λ* = 564–712 nm. SiR(d12) were excited using *λ* = 633 nm and emission signals were captured at *λ* = 637–740 nm. Emission signal were collected on 34 channel spectral detector (QUASAR, Zeiss), a typical gain of 750 over a scan area of 512 × 512 pxl.

Confocal and STED microscopy experiments on living Cox8A-SNAP:HeLa cells were performed in Life cell imaging buffer (Gibco) at 37 °C using a 100× (1.45 NA lambda oil) objective on a Nikon TiEclipse operated by Micromanager and equipped with a STEDyCON (Abberior Instruments) with 405/488/561/640 excitation and a 775 nm depletion laser. TMR(-d12) were excited using *λ* = 561 nm and emission signals were captured at *λ* = 580–630 nm. SiR(-d12) were excited using *λ* = 640 nm and emission was collected at *λ* = 650–700 nm. Both Emission signals were collected by a time gated APD (0.5–8 ns) with 8× signal accumulation and 100 nm pixel size for confocal images. STED images for SiR(-d12) were collected with 41% 775 nm depletion laser power and 15 nm pixel size.

Live STED images of microtubules in COS7 cells expressing Halo-Tubb5 labelled with Halo-SiR or Halo-SiR-d12 were acquired using *λ* = 640 nm excitation, 775 nm depletion and emission signals captured at *λ* = 655–700 nm using a time gated Hybrid detector (0.5–6 ns) with 4× line averaging. Images of 1024 × 1024 pixel had a pixel size of 18.9 nm. Line profiles of 100 microtubules were selected in 4 cells and intensity, sigma and FWHM = 2.35 × sigma were determined after Gaussian fitting (Image J).

LUXendin651(-d12) was imaged on a TIE Nikon epifluorescence microscope equipped with a pE4000 (cool LED), Penta Cube (AHF 66–615), 60× oil NA 1.49 (Apo TIRF Nikon) and a sCMOS camera (Prime 95B, Photometrics) operated by NIS Elements (Nikon). For excitation the following LED wavelengths were used: Hoechst: 405 nm; LUXendin651(-d12): 635 nm.

### smFRET: labelling of SBD2 and analysis by μsALEX spectroscopy

We followed our published protocols for labelling and imaging of SBD2.^31,32,51^ In brief, His-tagged SBD2 was incubated in buffer containing 1 mm DTT to keep all cysteine residues in a reduced state. Subsequently, SBD2 was immobilized on a Ni Sepharose 6 Fast Flow resin (GE Healthcare) and then incubated overnight at 4 °C with 25 nmol of each fluorophore dissolved in labelling buffer (50 mm Tris–HCl pH 7.6, 150 mm NaCl). Subsequently, SBD2 was washed with one column volume labelling buffer to remove unbound fluorophores. Fluorophore labelled SBD2 was then eluted with 1 mL of elution buffer (50 mm Tris–HCl pH 7.6, 150 mm NaCl, 500 mm imidazole). SBD2 was then further purified by size-exclusion chromatography (ÄKTA pure, Superdex 75 Increase 10/300 GL, GE Healthcare) to remove remaining fluorophores and aggregates. For all proteins, the labelling efficiency was higher than 40% for each labelling site (ESI Fig. 3†).

μsALEX microscopy was performed on a home-built microscope that was described in Gebhardt *et al.*^52^ ALEX experiments were carried out by diluting the labelled proteins to concentrations of ∼125 or ∼250 pm in 50 mm Tris–HCl pH 7.6, 150 mm NaCl supplemented with the ligand glutamine at concentrations described in the text. Before each experiment, the coverslip was passivated for 5 minutes with a 1 mg mL^−1^ BSA solution in imaging buffer. Data recording and analysis was described previously.^52^ Single-molecule events were identified using an all-photon and dual-colour burst-search^53^ algorithm with a threshold of 15, a time window of 500 μs and a minimum total photon number of 100.

### Transient absorption spectroscopy

Excited-state lifetimes were measured by transient absorption spectroscopy using a home built set up.^54^ Pump-pulses with a temporal duration of 110 fs are generated by converting the fundamental of a Ti:Sapphire laser by a TOPAS C (Light Conversion Ltd). The pump pulse energy was adjusted to 100 μW at the sample position. The excited-state dynamics were probed by a white light supercontinuum, generated by focusing a small portion of the fundamental laser into a CaF_2_ plate. The polarisation between the pump beam and the probe beam was set to magic angle (54.7°). The samples were placed in a 1 mm cuvette with PBS as solvent. The sample concentration was adjusted to yield an OD between 0.1 and 0.2 at the excitation wavelength. To ensure sample integrity, absorption spectra of the samples were measured before and after each experiment.

The transient absorption data were analysed using the Python-based KiMoPack software. Prior to analysis, the data was chirp corrected and globally fit using a sum of exponentials.

## Data availability

Raw data can be provided from the corresponding authors upon reasonable request.

## Author contributions

JB conceived and supervised the study. JB designed, and with KR and PP synthesized and characterized chemical compounds. KR, CC and JB performed *in vitro* measurements. KCA, JE, HG and ML performed cell culture, cell sorting and microscopy. TC designed smFRET experiments. NW conducted smFRET experiments and data analysis. KR, AV and BDI recorded and evaluated quantum yields using an integrating sphere and time-resolved experiments. BJ provided reagents. JB wrote the manuscript with input from all authors.

## Conflicts of interest

JB has a licensing deal with Celtarys Research for LUXendin distribution.

## Supplementary Material

SC-013-D1SC06466E-s001
