# Supplementary material for: N-Methyl deuterated rhodamines for protein labelling in sensitive fluorescence microscopy
Source: Chem Sci. 2022 Jun 28;13(29):8605–17. doi: 10.1039/d1sc06466e (PMC9337740; doi:10.1039/d1sc06466e)
Supplement: SC-013-D1SC06466E-s001 [file SC-013-D1SC06466E-s001.pdf]

Electronic Supplementary Material (ESI) for Chemical Science.
This journal is © The Royal Society of Chemistry 2022

Supplemental Information for:

N-Methyl deuterated rhodamines for protein labelling in sensitive fluorescence microscopy

Kilian Roßmann[1,7], Kerem C. Akkaya[2,7], Pascal Poc[1], Corentin Charbonnier[1], Jenny Eichhorst[2], Hannes Gonschior[2], Abha Valavalkar[5], Nicolas Wendler[6], Thorben Cordes[6], Benjamin Dietzek-Ivanšić[5], Ben Jones[3], Martin Lehmann[2,*], and Johannes Broichhagen[1,4,*]

[1] Leibniz-Forschungsinstitut für Molekulare Pharmakologie, Berlin, Germany.

[2] Department of Pharmacology and Cell Biology, Leibniz-Forschungsinstitut für Molekulare Pharmakologie, Berlin, Germany.

[3] Section of Endocrinology and Investigative Medicine, Imperial College London, London W12 0NN, United Kingdom.

[4] Department of Chemical Biology, Max Planck Institute for Medical Research, Heidelberg, Germany.

[5] Leibniz Institute for Photonic Technology Jena e.V. (Leibniz-IPHT), Research Department Functional Interfaces, Jena, Germany.

[6] Physical and Synthetic Biology, Faculty of Biology, Ludwig-Maximilians-Universität München, Großhadernerstr. 2-4, 82152 Planegg-Martinsried, Germany

[7] These authors contributed equally.

To whom correspondence should be addressed:
mlehmann@fmp-berlin.de and broichhagen@fmp-berlin.de

Table of Contents

1. General 4

2. Synthesis 6

2.1. General procedure A for fluorophore coupling 6

2.2. 2-(6-(Bis(methyl-d_3)amino)-3-(bis(methyl-d_3)iminio)-3H-xanthen-9-yl)-4-carboxybenzoate (2) 6

2.3. 4-((4-(((2-Amino-9H-purin-6-yl)oxy)methyl)benzyl)carbamoyl)-2-(6-(bis(methyl-d_3)amino)-3-(bis(methyl-d_3)iminio)-3H-xanthen-9-yl)benzoate (BG-TMR-d12) 7

2.4. 2-(6-(Bis(methyl-d_3)amino)-3-(bis(methyl-d_3)iminio)-3H-xanthen-9-yl)-4-((2-(2-((6-chlorohexyl)oxy)ethoxy)ethyl)carbamoyl)benzoate (CA-TMR-d12) 8

2.5. 3,7-Bis(bis(methyl-d_3)amino)-5,5-dimethyl-3'-oxo-3'H,5H-spiro[dibenzo[b,e]siline-10,1'-isobenzofuran]-6'-carboxylic acid (4) 9

2.6. 4-((4-(((2-Amino-9H-purin-6-yl)oxy)methyl)benzyl)carbamoyl)-2-(7-(bis(methyl-d_3)amino)-3-(bis(methyl-d_3)iminio)-5,5-dimethyl-3,5-dihydrodibenzo[b,e]silin-10-yl)benzoate (BG-SiR-d12) 10

2.7. 2-(7-(Bis(methyl-d_3)amino)-3-(bis(methyl-d_3)iminio)-5,5-dimethyl-3,5-dihydrodibenzo[b,e]silin-10-yl)-4-((2-(2-((6-chlorohexyl)oxy)ethoxy)ethyl)carbamoyl)benzoate (CA-SiR-d12) 11

2.8. 2-(6-(Bis(methyl-d_3)amino)-3-(bis(methyl-d_3)iminio)-3H-xanthen-9-yl)-4-((2-(2,5-dioxo-2,5-dihydro-1H-pyrrol-1-yl)ethyl)carbamoyl)benzoate (Mal-TMR-d12) 12

2.9. 2-(7-(Bis(methyl-d3)amino)-3-(bis(methyl-d3)iminio)-5,5-dimethyl-3,5-dihydrodibenzo[b,e]silin-10-yl)-4-((2-(2,5-dioxo-2,5-dihydro-1H-pyrrol-1-yl)ethyl)carbamoyl)benzoate (Mal-SiR-d12) 12

2.10. LUXendin651-d12 13

2.11. 7-(Dimethylamino)-4-methyl-2H-chromen-2-one (Coumarin 461) 13

2.12. 4-Methyl-7-((methyl-d3)amino)-2H-chromen-2-one (6) 14

2.13. 4-Methyl-7-(methyl(methyl-d3)amino)-2H-chromen-2-one (Coumarin 461-d3) 14

2.14. 7-(Bis(methyl-d3)amino)-4-methyl-2H-chromen-2-one (Coumarin 461-d6) 15

2.15. N,N-Dimethyl-7-nitrobenzo[c][1,2,5]oxadiazol-4-amine (NBD) 15

2.16. N,N-Bis(methyl-d3)-7-nitrobenzo[c][1,2,5]oxadiazol-4-amine (NBD-d6) 16

2.17. 3,7-Bis(dimethylamino)phenothiazin-5-ium (Methylene Blue) 16

2.18. 3,7-Bis(bis(methyl-d3)amino)phenothiazin-5-ium (Methylene Blue-d12) 17

3. NMR spectra 18

3.1. 2-(6-(Bis(methyl-d_3)amino)-3-(bis(methyl-d_3)iminio)-3H-xanthen-9-yl)-4-carboxybenzoate (2) 18

3.2. 4-((4-(((2-Amino-9H-purin-6-yl)oxy)methyl)benzyl)carbamoyl)-2-(6-(bis(methyl-d_3)amino)-3-(bis(methyl-d_3)iminio)-3H-xanthen-9-yl)benzoate (BG-TMR-d12) 20

3.3. 2-(6-(Bis(methyl-d_3)amino)-3-(bis(methyl-d_3)iminio)-3H-xanthen-9-yl)-4-((2-(2-((6-chlorohexyl)oxy)ethoxy)ethyl)carbamoyl)benzoate (CA-TMR-d12) 22

3.4. 3,7-Bis(bis(methyl-d_3)amino)-5,5-dimethyl-3'-oxo-3'H,5H-spiro[dibenzo[b,e]siline-10,1'-isobenzofuran]-6'-carboxylic acid (4) 24

3.5. 4-((4-(((2-Amino-9H-purin-6-yl)oxy)methyl)benzyl)carbamoyl)-2-(7-(bis(methyl-d_3)amino)-3-(bis(methyl-d_3)iminio)-5,5-dimethyl-3,5-dihydrodibenzo[b,e]silin-10-yl)benzoate (BG-SiR-d12) 26

3.6. 2-(7-(Bis(methyl-d_3)amino)-3-(bis(methyl-d_3)iminio)-5,5-dimethyl-3,5-dihydrodibenzo[b,e]silin-10-yl)-4-((2-(2-((6-chlorohexyl)oxy)ethoxy)ethyl)carbamoyl)benzoate (CA-SiR-d12) 28

3.7. 2-(6-(Bis(methyl-d_3)amino)-3-(bis(methyl-d_3)iminio)-3H-xanthen-9-yl)-4-((2-(2,5-dioxo-2,5-dihydro-1H-pyrrol-1-yl)ethyl)carbamoyl)benzoate (Mal-TMR-d12) 30

3.8. 2-(7-(bis(methyl-d3)amino)-3-(bis(methyl-d3)iminio)-5,5-dimethyl-3,5-dihydrodibenzo[b,e]silin-10-yl)-4-((2-(2,5-dioxo-2,5-dihydro-1H-pyrrol-1-yl)ethyl)carbamoyl)benzoate (Mal-SiR-d12) 32
3.9. 7-(Dimethylamino)-4-methyl-2H-chromen-2-one (Coumarin 461) 34
3.10. 4-Methyl-7-((methyl-d3)amino)-2H-chromen-2-one (6) 36
3.11. 4-Methyl-7-(methyl(methyl-d3)amino)-2H-chromen-2-one (Coumarin 461-d3) 38
3.12. 7-(Bis(methyl-d3)amino)-4-methyl-2H-chromen-2-one (Coumarin 461-d6) 40
3.13. N,N-Dimethyl-7-nitrobenzo[c][1,2,5]oxadiazol-4-amine (NBD) 42
3.14. N,N-Bis(methyl-d3)-7-nitrobenzo[c][1,2,5]oxadiazol-4-amine (NBD-d6) 44
3.15. 3,7-Bis(dimethylamino)phenothiazin-5-ium (Methylene Blue) 46
3.16. 3,7-Bis(bis(methyl-d3)amino)phenothiazin-5-ium (Methylene Blue-d12) 48

4. SNAP$_f$ construct 50

5. SNAP–Halo construct and mass spectrometry 50

6. Supplementary Figures 51

7. Supplementary Schemes 54

8. Supplementary Tables 56

9. References 57

1. General

All chemical reagents and anhydrous solvents for synthesis were purchased from commercial suppliers (Sigma-Aldrich, Fluka, Acros, Fluorochem, TCI) and were used without further purification if not stated otherwise. Commercial coumarin 461 and methylene blue were HPLC purified before concentration assessment and measuring photophysical properties to ensure similar purity and composition as their synthesizes, deuterated counterparts. BG-TMR and BG-SiR were described before.[1]

NMR spectra were recorded at 300 K in deuterated solvents on a Bruker AVANCE III HD 400 equipped with a CryoProbe or on Bruker AV-III spectrometers using either a cryogenically cooled 5 mm TCI-triple resonance probe equipped with one-axis self-shielded gradients or room-temperature 5 mm broadband probe and calibrated to residual solvent peaks ($^{1}H/^{13}C$ in ppm): DMSO-d_6 (2.50/39.52), MeOD-d_4 (3.31/49.00). Multiplicities are abbreviated as follows: s = singlet, d = doublet, t = triplet, q = quartet, p = pentet, h = heptet, br = broad, m = multiplet. Coupling constants *J* are reported in Hz. Spectra are reported based on appearance, not on theoretical multiplicities derived from structural information. This also concerns the report on ^{13}C NMR, where not all signals were obtained even after long-time recording (presumably quaternary carbons since they do not experience an NOE and relaxation is slower), which is annotated for the respective molecules below.

UPLC-UV/Vis for purity assessment was performed on a Waters H-class instrument equipped with a quaternary solvent manager, a Waters autosampler, a Waters TUV detector and a Waters Acquity QDa detector with an Acquity UPLC BEH C18 1.7 µm, 2.1 × 50 mm RP column (Waters Corp., USA). Buffer A: 0.1% TFA in H_2O Buffer B: 0.1% TFA in MeCN. The typical gradient was from 5% B for 0.5 min; gradient to 95% B over 3.0 min; 95% B for 0.9 min; gradient to 5% B over 1.1 min with 0.6 mL/min flow. LC-MS was performed on i) a Shimadzu MS2020 connected to a Nexera UHPLC system equipped with a Waters ACQUITY UPLC BEH C18 (1.7 µm, 50 × 2.1 mm). Buffer A: 0.1% FA in H_2O Buffer B: acetonitrile. The typical gradient was from 10% B for 0.5 min → gradient to 90% B over 4.5 min → 90% B for 0.5 min → gradient to 99% B over 0.5 min with 1 mL/min flow, or ii) an Agilent 1260 Infinity II LC System equipped with Agilent SB-C18 column (1.8 µm, 2.1 × 50 mm). Buffer A: 0.1% FA in H_2O Buffer B: 0.1% FA acetonitrile. The typical gradient was from 10% B for 0.5 min → gradient to 95% B over 5 min → 95% B for 0.5 min → gradient to 99% B over 1 min with 0.8 mL/min flow. Retention times (t_R) are given in minutes (min). Chromatograms were imported into Graphpad Prism8 and purity was determined by calculating AUC ratios.

Preparative or semi-preparative HPLC was performed on different instruments. i) A Shimadzu Prominence 20A system or a Shimadzu Prominence 8A system (both Shimadzu Corporation, Kyoto, Japan) equipped with columns as followed: preparative column – Nucleodur C18 HTec, 5 µm, 250x32 mm; semi-preparative column – Nucleodur C18 HTec, 5 µm, 250x21 mm (all columns purchased from Macherey-Nagel, GmbH & Co. KG, Düren, Germany). Eluents A (0.1% TFA in H_2O) and B (0.1% TFA in MeCN) were applied as a linear gradient. Peak detection was performed at maximal absorbance wavelength. ii) An Agilent 1260 Infinity II LC System equipped with columns as followed: preparative column –Reprospher 100 C18 columns (10 µm: 50 x 30 mm at 20 mL/min flow rate; semi-preparative column – 5 µm: 250 x 10 mm at 4 mL/min flow rate. Eluents A (0.1% TFA in H_2O) and B (0.1% TFA in MeCN) were applied as a linear gradient. Peak detection was performed at maximal absorbance wavelength. iii) A Waters e2695 system on a Supelco Ascentis® C18 HPLC Column (5 µm, 250 × 21.2 mm at 8 mL/min). Eluents A (0.1% TFA in H_2O) and B (0.1% TFA in MeCN) were applied as a linear gradient.

High resolution mass spectrometry was performed on different instruments. i) A Bruker maXis II ETD hyphenated with a Shimadzu Nexera system. The instruments were controlled via Brukers otofControl 4.1 and Hystar 4.1 SR2 (4.1.31.1) software. The acquisition rate was set to 3 Hz and the following source parameters were used for positive mode electrospray ionization: End plate offset = 500 V; capillary voltage = 3800 V; nebulizer gas pressure = 45 psi; dry gas flow = 10 L/min; dry temperature = 250 °C. Transfer, quadrupole and collision cell settings are mass range dependent and were fine-adjusted with consideration of the respective analyte's molecular weight. For internal calibration sodium format clusters were used. Samples were desalted via fast liquid chromatography. A Supelco TitanTM C18 UHPLC Column, 1.9 µm, 80 Å pore size, 20 × 2.1 mm and a 2 min gradient from 10 to 98% aqueous MeCN with 0.1% FA (H_2O: Carl Roth GmbH + Co. KG ROTISOLV® Ultra LC-MS; MeCN: Merck KGaA LiChrosolv® Acetonitrile hypergrade for LC-MS; FA - Merck KGaA LiChropur® Formic acid 98%–100% for LC-MS) was used for separation. Sample dilution in 10% aqueous MeCN (hyper grade) and injection volumes were chosen dependent of the analyte's ionization efficiency. Hence, on-column loadings resulted between 0.25–5.0 ng. Automated internal re-calibration and data analysis of the recorded spectra were performed with Bruker's DataAnalysis 4.4 SR1 software. ii) An Agilent Technologies 6230 series accurate mass TOF LC-MS linked to an Agilent Technologies 1290 Infinity Series machine with a Thermo Accucore™ RP-MS column, 2.6 µm pore size, 30 × 2.1 mm, and a 3 min gradient from 5 to 99% aqueous MeCN with 0.1% TFA and MeCN with 0.1% TFA. flow rate: 0.8 mL/min; UV-detection: 220 nm, 254 nm, 300 nm.

Intact proteins were analyzed using a Waters H-class instrument equipped with a quaternary Solvent manager, a Waters sample manager-FTN, a Waters PDA detector and a Waters column manager with an Acquity UPLC protein BEH C4 column (300 Å, 1.7 µm, 2.1 mm x 50 mm). Proteins were eluted with a flow rate of 0.3 mL/min at a column temperature of 80 °C. The following gradient was used: A: 0.01% FA in H_2O; B: 0.01% FA in MeCN. gradient 5-95% B from 0-6 min. Mass analysis was conducted with a Waters XEVO G2-XS QTof analyzer. Proteins were ionized in positive ion mode applying a cone voltage of 40 kV. Raw data was analyzed with MaxEnt 1. After deconvolution of the crude spectra, no single or non-labelled SNAP-Halo construct was observed, indicating complete reaction.

Flash column chromatography (FCC) was performed on a Biotage Isolera One with pre-packed silica columns (0.040–0.063 mm, 230-400 mesh, Silicycle). Reactions and chromatography fractions were monitored by thin layer chromatography (TLC) on Merck silica gel 60 F254 glass plates. The spots were visualized either under UV light at 254 nm and/or 366 nm or with appropriate staining method (iodine, *para*-anisaldehyde, $KMnO_4$) followed by heating.

2. Synthesis

2.1. General procedure A for fluorophore coupling

In an Eppendorf tube, 1.0 equiv. of deuterated carboxy-dye was dissolved in 100 µL/mg DMSO and 8.0 equiv. of DIPEA. Upon addition of 1.5 equiv. TSTU (from a 10 mg/mL stock in DMSO) the reaction mixture was vortexed and allowed to incubate for 10 min, before 1.5 equiv. of amine (BG-NH_2, CA-NH_2 or Mal-NH_2 (*N*-(2-Aminoethyl)-maleinimid-trifluoracetat; Aldrich: #56591) were added. The mixture was vortexed again and allowed to incubate for 60 min before it was quenched by addition of 20 equiv. of acetic acid and 25vol% of water. HPLC (MeCN:H_2O+0.1% TFA = 10:90 to 90:10 over 60 minutes) provided the desired compound, which was aliquoted to 5 nmol and obtained as a colorful powders after lyophilization.

2.2. 2-(6-(Bis(methyl-d$_3$)amino)-3-(bis(methyl-d$_3$)iminio)-3*H*-xanthen-9-yl)-4-carboxybenzoate (2)

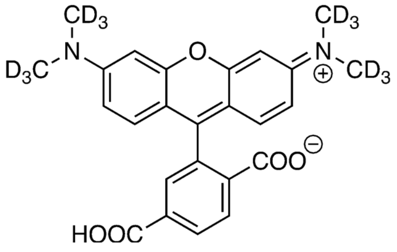

A Schlenk flask was charged under an argon atmosphere with 50.0 mg (71.8 µmol, 1.0 equiv.) of *tert*-butyl 3-oxo-3',6'-bis(((trifluoromethyl)sulfonyl)oxy)-3*H*-spiro[isobenzofuran-1,9'-xanthene]-6-carboxylate[2] (**1**), 13.2 mg (14.4 µmol, 0.2 equiv.) of tris(dibenzylideneacetone)-dipalladium(0) (Pd_2dba_3), 10.3 mg (21.5 µmol, 0.3 equiv.) of 2-(dicyclohexylphosphino)-2',4',6'-tri*iso*propylbiphenyl (XPhos), 112 mg (344 µmol, 4.8 equiv.) of Cs_2CO_3 and 18.9 mg (215 µmol, 3.0 equiv.) of $(D_3C)_2NH$ x HCl), before 2 mL of dry 1,4-dioxane were added via syringe. The reaction mixture was heated to 100 °C over night before it was cooled to room temperature and all volatiles were removed *in vacuo*. 10% TFA in DCM was added to the crude and the deprotection step was allowed to incubate at r.t. over 4 hours. After removal of all volatiles and reuptake of the crude in MeCN:H_2O = 1:1, HPLC (MeCN:H_2O+0.1% TFA = 10:90 to 90:10 over 60 minutes) provided 22.0 mg (49.7 µmol) of the desired compound as a red powder in 69% yield.

^{1}H NMR (600 MHz, MeOD-d$_4$): δ [ppm] = 8.42–8.37 (m, 2H), 7.97 (d, J = 1.2 Hz, 1H), 7.14 (d, J = 9.5 Hz, 2H), 7.04 (dd, J = 9.5, 2.5 Hz, 2H), 6.96 (d, J = 2.4 Hz, 2H).
^{13}C NMR (150 MHz, MeOD-d$_4$): δ [ppm] = 167.7, 167.4, 160.4, 159.1, 136.1, 135.9, 135.4, 132.8, 132.3, 132.0, 115.6, 114.9, 97.4. Two carbon signals missing.
HRMS (ESI): calc. for $C_{25}H_{11}D_{12}N_2O_5$ $[M+H]^+$: 443.2355, found: 443.2352.

2.3. 4-((4-(((2-Amino-9*H*-purin-6-yl)oxy)methyl)benzyl)carbamoyl)-2-(6-(bis(methyl-d_3)amino)-3-(bis(methyl-d_3)iminio)-3*H*-xanthen-9-yl)benzoate (BG-TMR-d12)

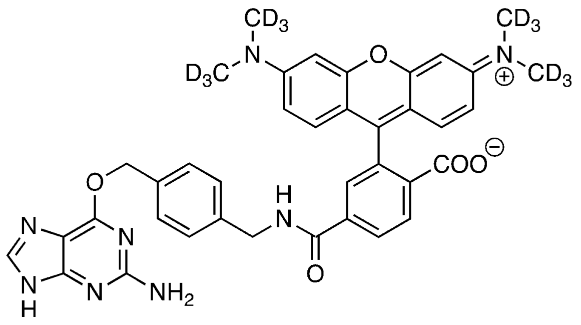

BG–TMR–d12 was prepared according to general procedure A.

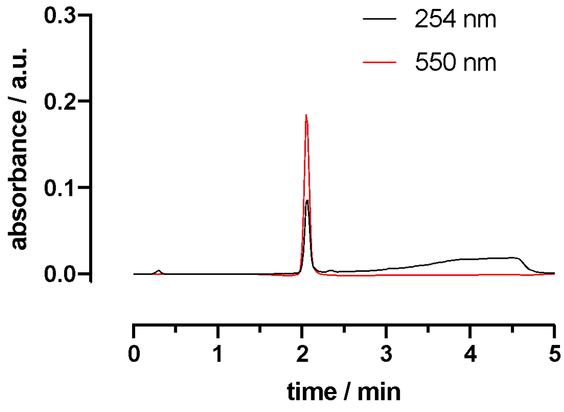

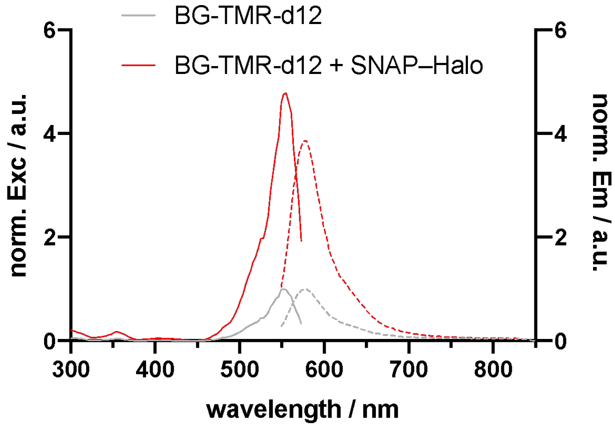

^{1}H NMR (600 MHz, MeOD): δ [ppm] = 8.41 (d, J = 8.3 Hz, 1H), 8.22 (d, J = 8.3, 1H), 8.20 (s, 1H), 7.85 (s, 1H), 7.51 (d, J = 7.9, 2H), 7.40 (d, J = 7.9 Hz, 2H), 7.14 (d, J = 9.5 Hz, 2H), 7.04 (dd, J = 9.5, 2.2 Hz, 2H), 6.98 (d, J = 2.2 Hz, 2H) 5.60 (s, 2H), 4.60 (s, 2H).
^{13}C NMR (150 MHz, MeOD): δ [ppm] = 167.8, 167.3, 161.1, 160.5, 159.1, 159.0, 154.4, 142.5, 140.4, 139.2, 135.9, 135.6, 135.0, 132.8, 132.0, 130.3, 130.0, 129.9, 128.9, 115.5, 114.8, 97.3, 70.2, 44.4. Two carbon signals missing.
HRMS (ESI): calc. for $C_{38}H_{24}D_{12}N_8O_5$ $[M+2H]^{2+}$: 348.1776, found: 348.1770.

2.4. 2-(6-(Bis(methyl-d3)amino)-3-(bis(methyl-d3)iminio)-3*H*-xanthen-9-yl)-4-((2-(2-((6-chlorohexyl)oxy)ethoxy)ethyl)carbamoyl)benzoate (CA-TMR-d12)

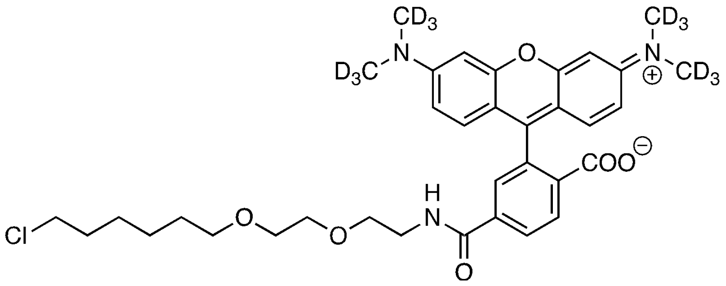

CA–TMR–d12 was prepared according to general procedure A.

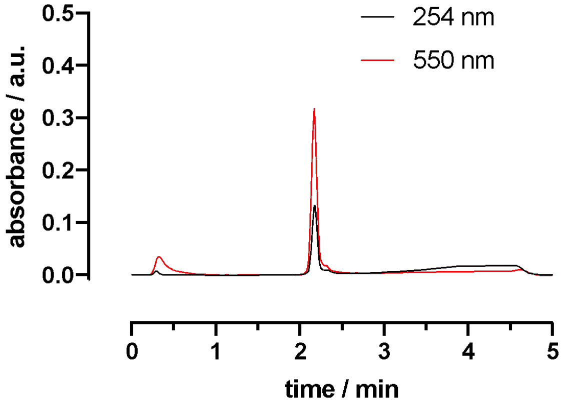

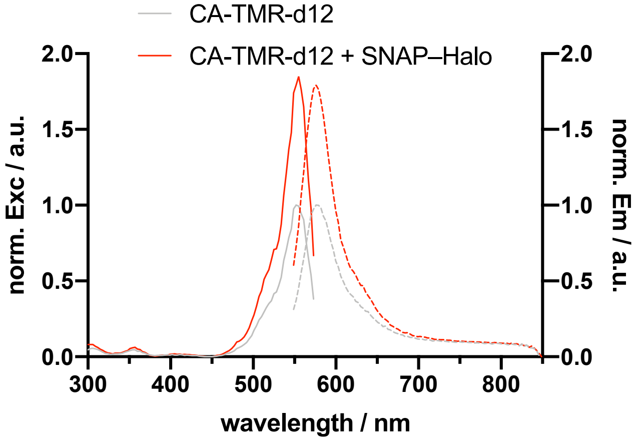

^{1}H NMR (600 MHz, MeOD-d$_4$): δ [ppm] = 8.79 (t, J = 5.1 Hz, 1H), 8.41 (d, J = 8.2 Hz, 1H) 8.22 (dd, J = 8.3, 1.6 Hz, 1H), 7.83 (d, J = 1.4 Hz, 1H), 7.16 (d, J = 9.5 Hz, 2H) 7.06 (dd, J = 9.5, 2.4 Hz, 2H), 6.98 (d, J = 2.4 Hz, 2H), 3.66 (t, J = 5.3 Hz, 2H), 3.64–3.58 (m, 4H) 3.55 (m, 2H), 3.53 (t, J = 6.63 Hz, 2H), 3.43 (t, J = 6.5 Hz, 2H), 1.71 (quint, J = 7.1 Hz, 2H), 1.50 (quint, J = 7.0 Hz, 2H) 1.40 (quint, J = 7.5 Hz, 2H), 1.32 (quint, J = 5.1 Hz, 2H).
^{13}C NMR (150 MHz, MeOD-d$_4$): δ [ppm] = 168.0, 167.3, 160.6, 159.1, 159.1, 139.4, 135.5, 134.8, 132.8, 132.1, 130.3, 130.1, 115.5, 114.9, 97.4, 72.1, 71.2, 71.2, 70.4, 45.7, 41.2, 33.7, 30.5, 27.7, 26.4.
HRMS (ESI): calc. for $C_{35}H_{31}D_{12}ClN_3O_6$ [M+H]$^+$: 648.3588, found: 648.3587.

2.5. 3,7-Bis(bis(methyl-d$_3$)amino)-5,5-dimethyl-3'-oxo-3'*H*,5*H*-spiro[dibenzo[*b,e*]siline-10,1'-isobenzofuran]-6'-carboxylic acid (4)

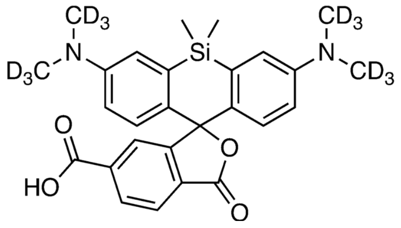

A Schlenk flask was charged under an argon atmosphere with 50.0 mg (67.7 µmol, 1.0 equiv.) of *tert*-butyl 5,5-dimethyl-3'-oxo-3,7-bis(((trifluoromethyl)sulfonyl)oxy)-3'*H*,5*H*-spiro[dibenzo[*b,e*]-siline-10,1'-isobenzo-furan]-6'-carboxylate[2] (**3**), 12.4 mg (13.5 µmol, 0.2 equiv.) of tris(dibenzylideneacetone)dipalladium(0) (Pd_2dba_3), 9.7 mg (20.3 µmol, 0.3 equiv.) of 2-(dicyclohexylphosphino)-2',4',6'-tri*iso*propylbiphenyl (XPhos), 106 mg (325 µmol, 4.8 equiv.) of Cs_2CO_3 and 17.8 mg (203 µmol, 3.0 equiv.) of $(D_3C)_2NH$ x HCl), before 2 mL of dry 1,4-dioxane were added via syringe. The reaction mixture was heated to 100 °C over night before it was cooled to room temperature and all volatiles were removed *in vacuo*. 10% TFA in DCM was added to the crude and the deprotection step was allowed to incubate at r.t. over 4 hours. After removal of all volatiles and reuptake of the crude in MeCN:H_2O = 1:1, HPLC (MeCN:H_2O+0.1% TFA = 10:90 to 90:10 over 60 minutes) provided 8.2 mg (16.9 µmol) of the desired compound as a purple-blue powder in 25% yield.

^{1}H NMR (600 MHz, MeOD-d$_4$): δ [ppm] = 8.34 (m, 2H), 7.83 (s, 1H), 7.35 (d, J = 2.75, 2H), 6.96 (d, J = 9.5 Hz, 2H), 6.77 (dd, J = 9.5, 2.4 Hz, 2H), 0.66 (s, 3H), 0.58 (s, 3H).
^{13}C NMR (150 MHz, MeOD-d$_4$): δ [ppm] = 168.1, 168.0, 154.8, 147.7, 144.3, 140.0, 135.6, 135.2, 131.7, 131.6, 131.1, 130.0, 121.5, 115.3, -0.7, -1.8. One carbon signal missing.
HRMS (ESI): calc. for $C_{27}H_{17}D_{12}N_2O_4Si$ $[M+H]^+$: 485.2644, found: 485.2645.

2.6. 4-((4-(((2-Amino-9*H*-purin-6-yl)oxy)methyl)benzyl)carbamoyl)-2-(7-(bis(methyl-d$_3$)amino)-3-(bis(methyl-d$_3$)iminio)-5,5-dimethyl-3,5-dihydrodibenzo[*b,e*]silin-10-yl)benzoate (BG-SiR-d12)

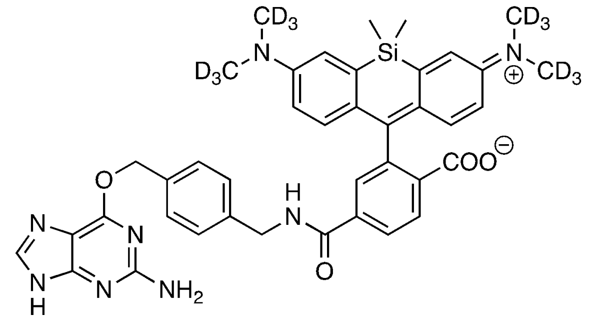

BG–SiR–d12 was prepared according to general procedure A.

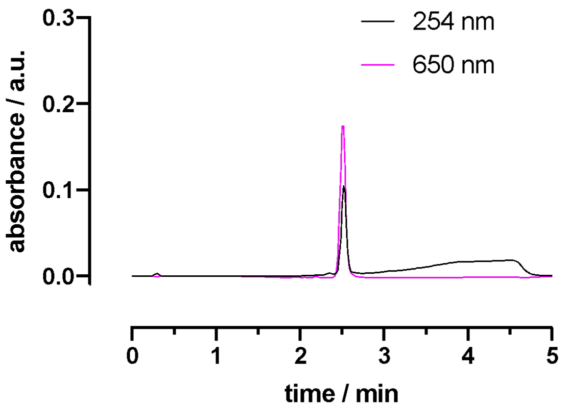

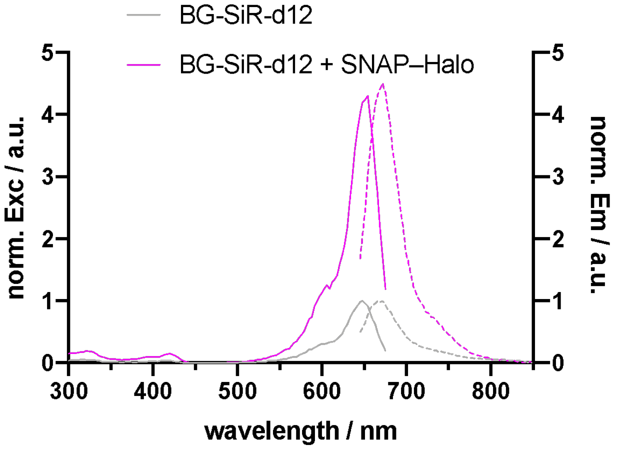

^{1}H NMR (600 MHz, MeOD-d$_4$): δ [ppm] = 8.33 (s, 1H), 8.30 (d, J = 8.2 Hz, 1H) 8.13 (d, J = 3.3 Hz, 1H), 7.72 (d, J = 1.1 Hz, 1H) 7.74 (d, J = 1.4 Hz, 1H), 7.50 (d, J = 8.0 Hz, 2H), 7.32 (d, J = 2.8 Hz, 2H), 6.95 (d, J = 9.5 Hz, 2H) 6.73 (dd, J = 9.5, 2.8 Hz, 2H) 5.62 (s, 2H), 4.58 (s, 2H), 0.64 (s, 3H), 0.58 (s, 3H).
^{13}C NMR (150 MHz, MeOD-d$_4$): δ [ppm] = 168.1, 167.9, 161.1, 158.0, 154.9, 153.4, 148.2, 144.1, 143.8, 140.7, 138.9, 135.4, 134.3, 131.9, 130.2, 129.9, 129.8, 129.0, 128.9, 121.5, 118.6, 116.7, 115.2, 70.9, 44.5, -0.8, -1.7. One carbon signal missing.
HRMS (ESI): calc. for $C_{40}H_{30}D_{12}N_8O_4Si$ $[M+2H]^{2+}$: 369.1920, found: 369.1917.

2.7. 2-(7-(Bis(methyl-d3)amino)-3-(bis(methyl-d3)iminio)-5,5-dimethyl-3,5-dihydrodibenzo[*b,e*]silin-10-yl)-4-((2-(2-((6-chlorohexyl)oxy)ethoxy)ethyl)carbamoyl)benzoate (CA-SiR-d12)

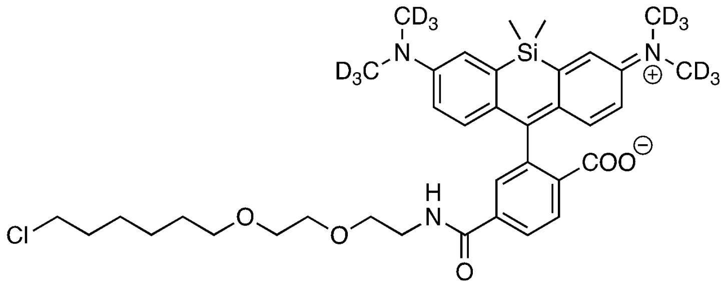

CA–SiR–d12 was prepared according to general procedure A.

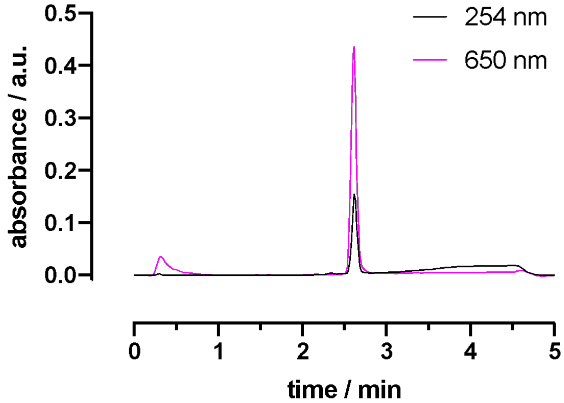

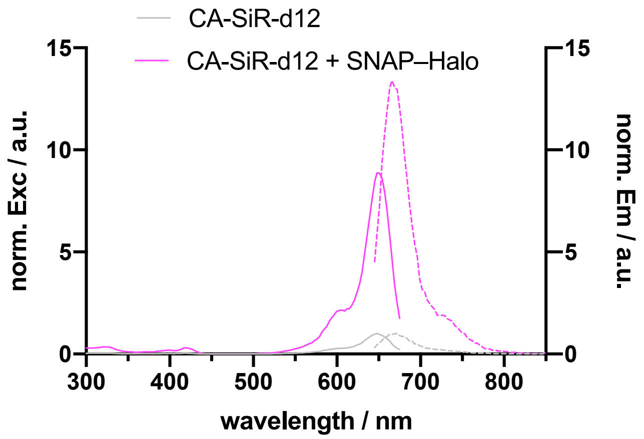

^{1}H NMR (600 MHz, MeOD-d$_4$): δ [ppm] = 8.13 (d, J = 7.9 Hz, 1H), 8.12 (d, J = 8.2 Hz, 1H), 7.72 (s, 1H), 7.32 (d, J = 2.6 Hz, 2H), 6.98 (d, J = 9.5 Hz, 2), 6.75 (dd, J = 9.5, 2.4 Hz, 2H), 3.65 (t, J = 5.3 Hz, 2H), 3.63–3.60 (m, 2H), 3.60–3.54 (m, 4H), 3.51 (t, J = 6.6 Hz, 2H), 3.43 (t, J = 6.5 Hz, 2H), 1.71 (quint, J = 7.0 Hz, 2H), 1.51 (quint, J = 7.0 Hz, 2H), 1.40 (quint, J = 7.5 Hz, 2H), 1.32 (quint, J = 8.1 Hz, 2H), 0.64 (s, 3H), 0.59 (s, 3H).
^{13}C NMR (150 MHz, MeOD-d$_4$): δ [ppm] = 168.1 167.8, 155.1, 148.5, 140.9, 139.0, 134.4, 132.0, 129.8, 121.5, 115.1, 72.1, 71.1, 70.4 45.7, 41.1 33.7, 30.7, 30.4, 27.6, 26.4, -0.88, -1.68. Four carbon signals missing.
HRMS (ESI): calc. for $C_{37}H_{37}D_{12}ClN_3O_5Si$ $[M+H]^+$: 690.3878, found: 690.3874.

2.8. 2-(6-(Bis(methyl-d$_3$)amino)-3-(bis(methyl-d$_3$)iminio)-3*H*-xanthen-9-yl)-4-((2-(2,5-dioxo-2,5-dihydro-1*H*-pyrrol-1-yl)ethyl)carbamoyl)benzoate (Mal-TMR-d12)

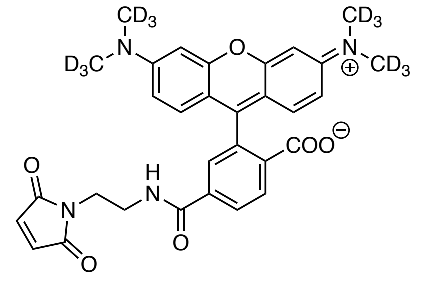

Mal–TMR–d12 was prepared according to general procedure A.

^{1}H NMR (600 MHz, DMSO-d$_6$): δ [ppm] = 8.79 (t, J = 4.8 Hz, 1H), 8.50 (br s, 1H), 8.05–8.03 (m, 2H), 7.51 (s, 1H), 6.95 (s, 1H), 6.52–6.48 (m, 6H), 3.52 (t, J = 4.9 Hz, 2H), 3.35–3.32 (m, 2H).
^{13}C NMR (150 MHz, DMSO-d$_6$): δ [ppm] = 170.9, 168.1, 165.0, 152.8, 152.1, 152.0, 140.4, 134.5, 128.9, 128.4, 124.7, 122.3, 116.4, 109.0, 105.5, 97.9, 84.7, 37.8, 36.9.
HRMS (ESI): calc. for $C_{31}H_{17}D_{12}N_4O_6$ $[M+H]^+$: 565.2835, found: 565.2879.

2.9. 2-(7-(Bis(methyl-d3)amino)-3-(bis(methyl-d3)iminio)-5,5-dimethyl-3,5-dihydrodibenzo[b,e]silin-10-yl)-4-((2-(2,5-dioxo-2,5-dihydro-1H-pyrrol-1-yl)ethyl)carbamoyl)benzoate (Mal-SiR-d12)

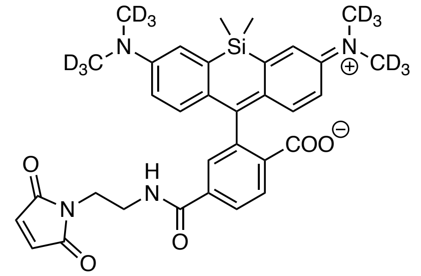

Mal–SiR–d12 was prepared according to general procedure A.

^{1}H NMR (600 MHz, DMSO-d$_6$): δ [ppm] = 8.91 (t, J = 5.6 Hz, 0.5H), 8.82 (t, J = 6.0 Hz, 0.7H), 8.09–8.04 (m, 1.2 H), 7.96 (d, J = 8.0 Hz, 0.6H), 7.68 (s, 0.4H), 7.58 (s, 0.6H), 7.09 (br s, 1.5H), 6.96 (s, 0.7H), 6.71–6.65 (m, 3.0H), 3.56–3.50 (m, 2.1H), 3.37 (q, J = 5.7 Hz, 1.3H), 2.86 (q, J = 7.3 Hz, 1.8H), 2.58 (t, J = 7.0 Hz, 0.9H), 0.64 (s, 3H), 0.53–0.52 (m, 3H). Two isomers.
^{13}C NMR (150 MHz, DMSO-d$_6$): δ [ppm] = 172.6, 171.0, 165.2, 164.9, 158.5, 158.3, 158.1, 157.8, 149.3, 139.6, 134.5, 130.6, 128.2, 128.0, 125.6, 122.7, 114.6, 113.9, 38.8, 37.8, 36.9, 36.7, 33.6, 33.0, -0.1, -1.3, -1.4. Two isomers, some carbons missing.
HRMS (ESI): calc. for $C_{31}H_{22}D_{12}N_4O_5Si$ $[M+H]^+$: 607.3124, found: 607.3192.

2.10. LUXendin651-d12

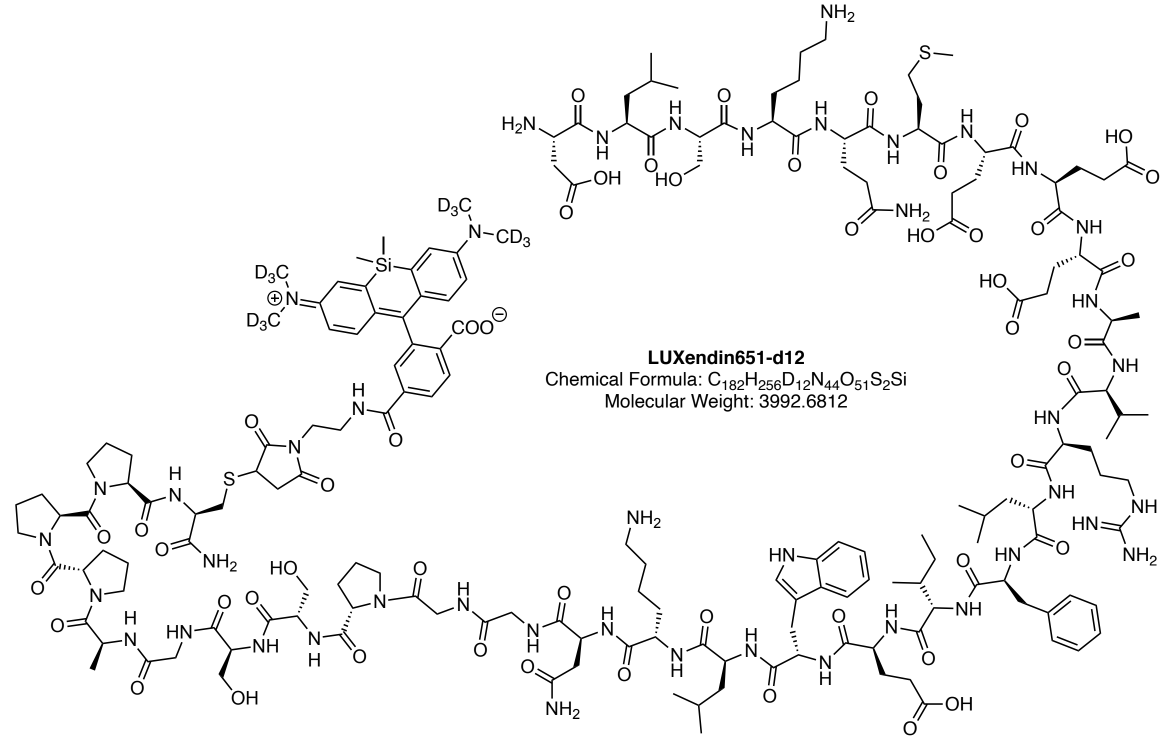

LUXendin651-d12 was prepared as described previously by using Mal-SiR-d_{12} instead of Mal-SiR.[3] Briefly, 400 nmol Ex4(9-39)_S39C obtained from solid-phase peptide synthesis[3] was incubated with 500 nmol SiR-d12-Mal in PBS and allowed to incubate at room temperature o.n. before the reaction was submitted to RP-HPLC to obtain 75 nmol of LUXendin651-d12 in 19% yield. LCMS trace is shown in Supporting Figure 2.

MS (ESI): calc. for $C_{182}H_{259}D_{12}N_{44}O_{51}S_2Si$ $[M+3H]^{3+}$: 1331.3315, found: 1331.9871.

2.11. 7-(Dimethylamino)-4-methyl-2*H*-chromen-2-one (Coumarin 461)

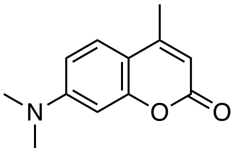

Preparative RP-HPLC provided the desired compound as a faint yellow powder after lyophilization.

^{1}H NMR (600 MHz, DMSO-d_6): δ [ppm] = 7.52 (d, J = 8.9 Hz, 1H), 6.73 (dd, J = 8.9, 2.5 Hz, 1H), 6.53 (d, J = 2.5 Hz, 1H), 5.96 (d, J = 0.8 Hz, 1H), 3.02 (s, 3H), 2.34 (d, J = 0.8 Hz, 3H).
^{13}C NMR (150 MHz, DMSO-d_6): δ [ppm] = 161.1, 155.7, 154.0, 153.3, 126.4, 109.4, 109.2, 108.6, 97.9, 40.2, 18.4.
HRMS (ESI): calc. for $C_{12}H_{14}NO_2$ $[M+H]^+$: 204.1019, found: 204.1018.

2.12. 4-Methyl-7-((methyl-d3)amino)-2*H*-chromen-2-one (6)

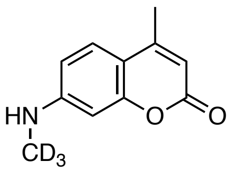

A round bottom flask was charged with 65.0 mg (371 µmol, 1.0 equiv.) of 7-amino-4-methyl-2*H*-chromen-2-one (**5**) and dissolved in 3 mL of MeCN before 102 mg (742.0 µmol, 2.0 equiv.) of K_2CO_3 and 135 mg (928 µmol, 57.8 µL, 2.5 equiv.) iodomethane-d_3 were added. The reaction mixture was heated to 80 °C for 3 h before it was cooled to room temperature and all volatiles were removed *in vacuo*. HPLC (MeCN:H_2O+0.1% TFA = 10:90 to 90:10 over 60 minutes) provided 12.5 mg (65.1 µmol) of the desired compound as a beige powdered side product in 18% yield. The d6 congener was isolated in 27% yield (*vide infra*).

^{1}H NMR (600 MHz, DMSO-d_6): δ [ppm] = 7.44 (d, J = 8.7 Hz, 1H), 6.63 (s, 1H), 6.58 (dd, J = 8.7, 2.3 Hz, 1H), 6.35 (d, J = 2.3 Hz, 1H), 5.91 (d, J = 0.9 Hz, 1H), 2.31 (d, J = 0.8 Hz, 3H).
^{13}C NMR (150 MHz, DMSO-d_6): δ [ppm] = 161.2, 156.2, 154.2, 153.9, 126.3, 110.5, 109.1, 107.8, 96.3, 18.5.
HRMS (ESI): calc. for $C_{11}H_9D_3NO_2$ $[M+H]^+$: 193.1051, found: 193.1051.

2.13. 4-Methyl-7-(methyl(methyl-d3)amino)-2*H*-chromen-2-one (Coumarin 461-d3)

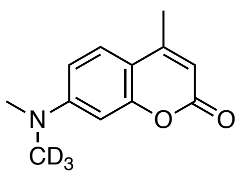

A round bottom flask was charged with 12.0 mg (62.5 µmol, 1.0 equiv.) of 4-Methyl-7-((methyl-d3)amino)-2*H*-chromen-2-one (**6**) and dissolved in 3 mL of MeCN before 100 mg (725.0 µmol, 11.6 equiv.) of K_2CO_3 and 135 mg (2.4 mmol, 150 µL, 38 equiv.) iodomethane were added. The reaction mixture was heated to 80 °C for 12 h before it was cooled to room temperature and all volatiles were removed *in vacuo*. HPLC (MeCN:H_2O+0.1% TFA = 10:90 to 90:10 over 60 minutes) provided 10.0 mg (48.3 µmol) of the desired compound as a beige powdered side product in 77% yield.

^{1}H NMR (600 MHz, DMSO-d_6): δ [ppm] = 7.52 (d, J = 8.9 Hz, 1H), 6.72 (dd, J = 8.9, 2.6 Hz, 1H), 6.53 (d, J = 2.6 Hz, 1H), 5.96 (d, J = 1.1 Hz, 1H), 3.01 (s, 3H), 2.33 (d, J = 1.1 Hz, 3H).
^{13}C NMR (150 MHz, DMSO-d_6): δ [ppm] = 161.2, 155.7, 154.1, 153.4, 126.4, 109.4, 109.2, 108.6, 97.8, 18.4. One carbon masked by DMSO-d_6.
HRMS (ESI): calc. for $C_{12}H_{11}D_3NO_2$ $[M+H]^+$: 207.1207, found: 207.1205.

2.14. 7-(Bis(methyl-d3)amino)-4-methyl-2*H*-chromen-2-one (Coumarin 461-d6)

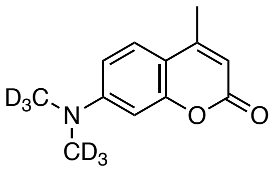

A round bottom flask was charged with 65.0 mg (371 µmol, 1.0 equiv.) of 7-amino-4-methyl-2*H*-chromen-2-one (**5**) and dissolved in 3 mL of MeCN before 102 mg (742.0 µmol, 2.0 equiv.) of K_2CO_3 and 135 mg (928 µmol, 57.8 µL2.5 equiv.) iodomethane-d_3 were added. The reaction mixture was heated to 80 °C for 3 h before it was cooled to room temperature and all volatiles were removed *in vacuo*. HPLC (MeCN:H_2O+0.1% TFA = 10:90 to 90:10 over 60 minutes) provided 21.2 mg (101.4 µmol) of the desired compound as a beige powder in 27% yield.

^{1}H NMR (600 MHz, DMSO-d_6): δ [ppm] = 7.52 (d, J = 8.9 Hz, 1H), 6.72 (dd, J = 8.9, 2.6 Hz, 1H), 6.53 (d, J = 2.5 Hz, 1H), 5.96 (d, J = 1.1 Hz, 1H), 2.33 (d, J = 1.1 Hz, 3H).
^{13}C NMR (150 MHz, DMSO-d_6): δ [ppm] = 161.2, 155.7, 154.0, 153.4, 126.3, 109.4, 108.5, 97.8, 18.4.
HRMS (ESI): calc. for $C_{12}H_8D_6NO_2$ $[M+H]^+$: 210.1394, found: 210.1396.

2.15. *N*,*N*-Dimethyl-7-nitrobenzo[*c*][1,2,5]oxadiazol-4-amine (NBD)

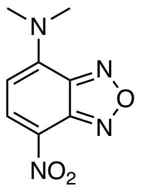

A round bottom flask was charged with 30.0 mg (150.3 µmol, 1.0 equiv.) of 4-chloro-7-nitro-2,1,3-benzoxadiazole (**7**) and dissolved in 3 mL of anhydrous EtOH before 24.3 mg (301 µmol, 2.0 equiv.) dimethylamine hydrochloride and 60 µL triethylamine (431 µmol, 3.0 equiv.) were added. The reaction mixture was stirred for 4 h before all volatiles were removed *in vacuo*. HPLC (MeCN:H_2O+0.1% TFA = 10:90 to 90:10 over 60 minutes) provided 18.6 mg (89.4 µmol) of the desired compound as a bright orange powder in 59% yield. Crystals suitable for X-ray crystallography were obtained as fine needles by allowing a 5 mg/mL solution in DMSO stand open to the atmosphere for one week.

^{1}H NMR (600 MHz, DMSO-d_6): δ [ppm] = 8.46 (d, J = 9.2 Hz, 1H), 6.36 (d, J = 9.2 Hz, 1H), 3.60 (br s, 6H).
^{13}C NMR (150 MHz, DMSO-d_6): δ [ppm] =146.6, 144.9, 144.8, 136.2, 119.7, 102.2, 43.5 (br).
HRMS (ESI): calc. for $C_8H_9N_4O_3$ $[M+H]^+$: 209.0669, found: 209.0673.

2.16. *N*,*N*-Bis(methyl-d3)-7-nitrobenzo[*c*][1,2,5]oxadiazol-4-amine (NBD-d6)

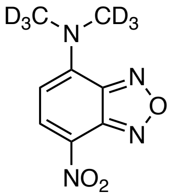

A round bottom flask was charged with 30.0 mg (150.3 µmol, 1.0 equiv.) of 4-chloro-7-nitro-2,1,3-benzoxadiazole (**7**) and dissolved in 3 mL of anhydrous EtOH before 26.3 mg (301 µmol, 2.0 equiv.) dimethyl-d6-amine hydrochloride and 60 µL triethylamine (431 µmol, 3.0 equiv.) were added. The reaction mixture was stirred for 4 h before all volatiles were removed *in vacuo*. HPLC (MeCN:H_2O+0.1% TFA = 10:90 to 90:10 over 60 minutes) provided 29.2 mg (136.4 µmol) of the desired compound as a bright orange powder in 91% yield. Crystals suitable for X-ray crystallography were obtained as fine needles by allowing a 5 mg/mL solution in DMSO stand open to the atmosphere for one week.

^{1}H NMR (600 MHz, DMSO-d_6): δ [ppm] = 8.46 (d, J = 9.2 Hz, 1H), 6.36 (d, J = 9.2 Hz, 1H).
^{13}C NMR (150 MHz, DMSO-d_6): δ [ppm] = 147.2, 145.3, 145.3, 136.7, 120.2, 102.6.
HRMS (ESI): calc. for $C_8H_3D_6N_4O_3$ $[M+H]^+$: 215.1046, found: 215.1046.

2.17. 3,7-Bis(dimethylamino)phenothiazin-5-ium (Methylene Blue)

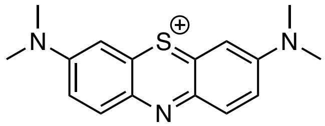

Preparative RP-HPLC provided the desired compound as a blue powder after lyophilization.

^{1}H NMR (600 MHz, DMSO-d_6): δ [ppm] = 7.92 (d, J = 9.2 Hz, 2H), 7.51–7.48 (m, 4H), 3.37 (s, 12H).
^{13}C NMR (150 MHz, DMSO-d_6): δ [ppm] = 153.9, 137.8, 135.0, 133.5, 119.0, 106.8, 41.0.
HRMS (ESI): calc. for $C_{16}H_{18}N_3S$ $[M]^+$: 296.1969, found: 296.1972.

2.18. 3,7-Bis(bis(methyl-d3)amino)phenothiazin-5-ium (Methylene Blue-d12)

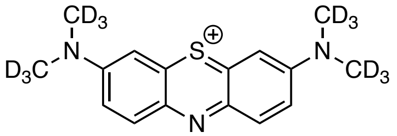

A round bottom flask was charged with 20 mg (69.6 µmol, 1.0 equiv.) of thionine acetate (**8**) and dissolved in 5 mL of MeCN before 96 mg (696 µmol, 10.0 equiv.) of K_2CO_3 and 101 mg (696 µmol, 44.3 µL, 10.0 equiv.) iodomethane-d_3 were added. The reaction mixture was heated to 80 °C for 3 h before it was cooled to room temperature and all volatiles were removed *in vacuo*. Preparative RP-HPLC provided 14 mg (34.2 µmol as TFA salt) of the desired compound as a blue powder after lyophilization in 49% yield.

^{1}H NMR (600 MHz, DMSO-d_6): δ [ppm] = 7.86 (d, J = 9.0 Hz, 2H), 7.45–7.42 (m, 4H).
^{13}C NMR (150 MHz, DMSO-d_6): δ [ppm] = 154.0, 137.9, 135.0, 133.6, 119.0, 106.7.
HRMS (ESI): calc. for $C_{16}H_6D_{12}N_3S$ $[M]^+$: 284.1216, found: 284.1216.

3. NMR spectra

3.1. 2-(6-(Bis(methyl-d$_3$)amino)-3-(bis(methyl-d$_3$)iminio)-3*H*-xanthen-9-yl)-4-carboxybenzoate (2)

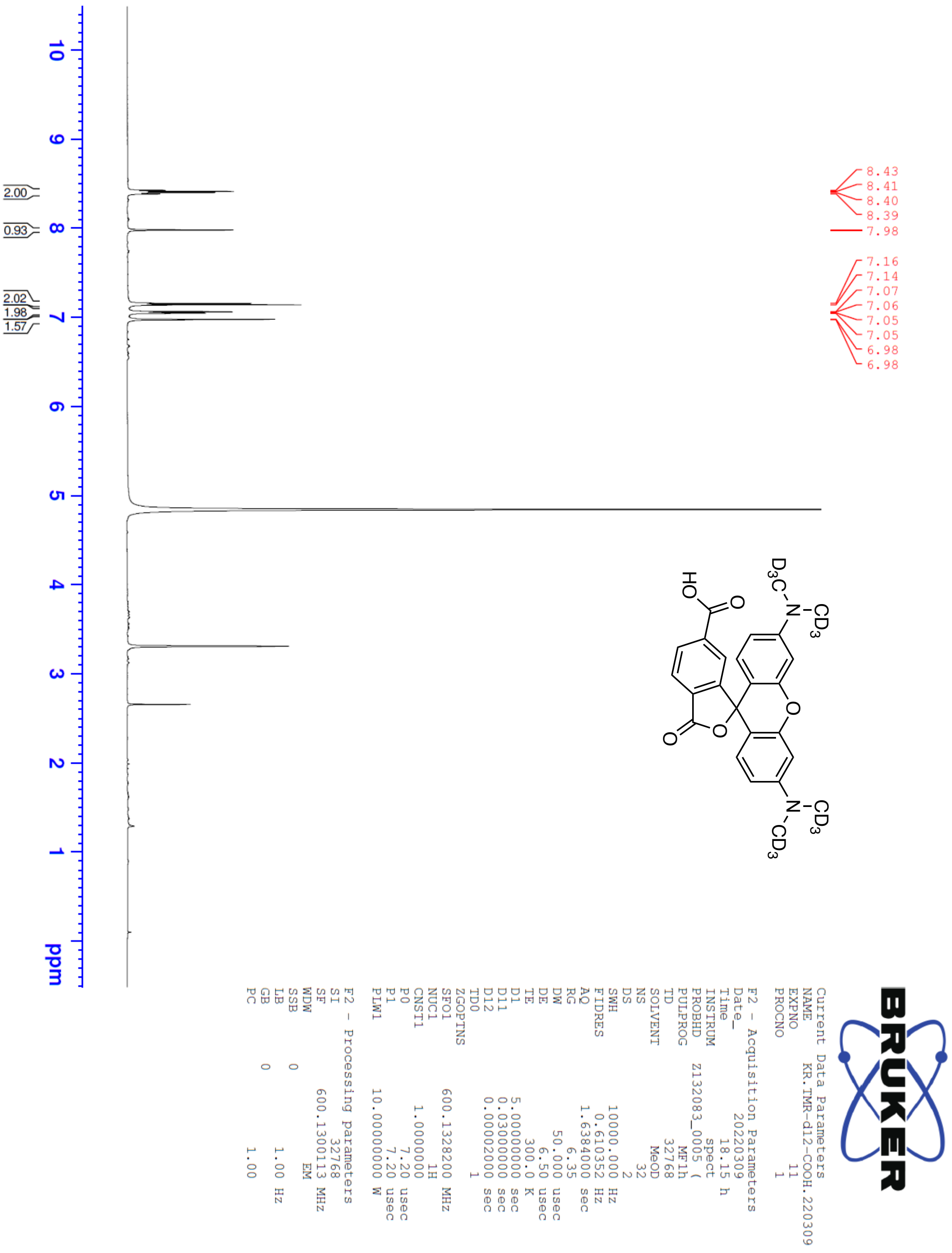

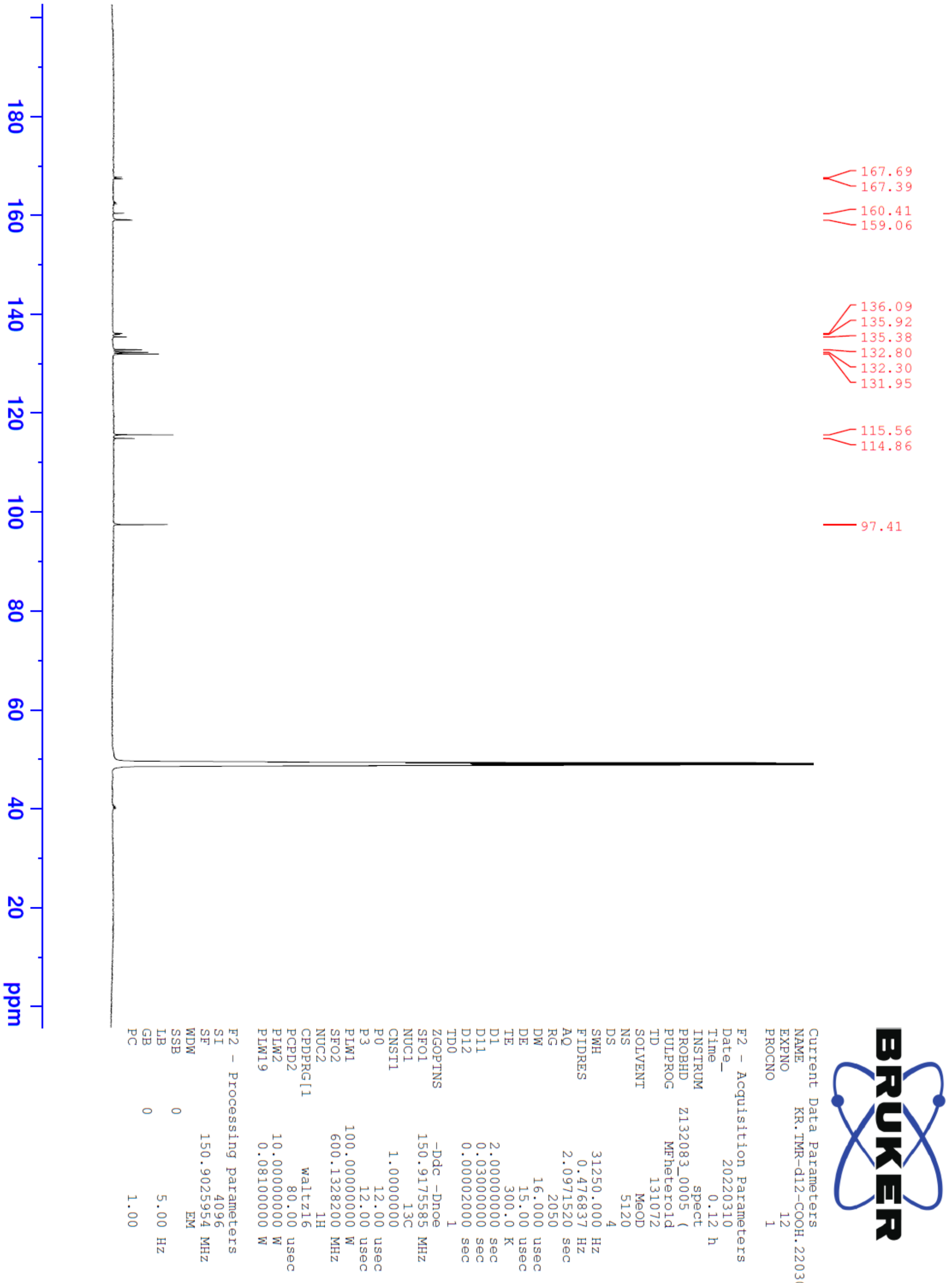
167.69
167.39
160.41
159.06
136.09
135.92
135.38
132.80
132.30
131.95
115.56
114.86
97.41
180
160
140
120
100
80
60
40
20
ppm
BRUKER
Current Data Parameters
NAME KR.TMR-d12-COOH.220309
EXPNO 12
PROCNO 1
F2 - Acquisition Parameters
Date_ 20220310
Time 0.12 h
INSTRUM spect
PROBHD Z132083_0005 (
PULPROG MFhetero1d
TD 131072
SOLVENT MeOD
NS 5120
DS 4
SWH 31250.000 Hz
FIDRES 0.476837 Hz
AQ 2.0971520 sec
RG 2050
DW 16.000 usec
DE 15.00 usec
TE 300.0 K
D1 2.00000000 sec
D11 0.03000000 sec
D12 0.00002000 sec
TD0 1
ZGOPTNS -Ddc -Dnoe
SFO1 150.9175585 MHz
NUC1 13C
CNST1 1.0000000
P0 12.00 usec
P3 12.00 usec
PLW1 100.00000000 W
SFO2 600.1328200 MHz
NUC2 1H
CPDPRG[1 waltz16
PCPD2 80.00 usec
PLW2 10.00000000 W
PLW19 0.08100000 W
F2 - Processing parameters
SI 4096
SF 150.9025954 MHz
WDW EM
SSB 0
LB 5.00 Hz
GB 0
PC 1.00

3.2. 4-((4-(((2-Amino-9*H*-purin-6-yl)oxy)methyl)benzyl)carbamoyl)-2-(6-(bis(methyl-d$_3$)amino)-3-(bis(methyl-d$_3$)iminio)-3*H*-xanthen-9-yl)benzoate (BG-TMR-d12)

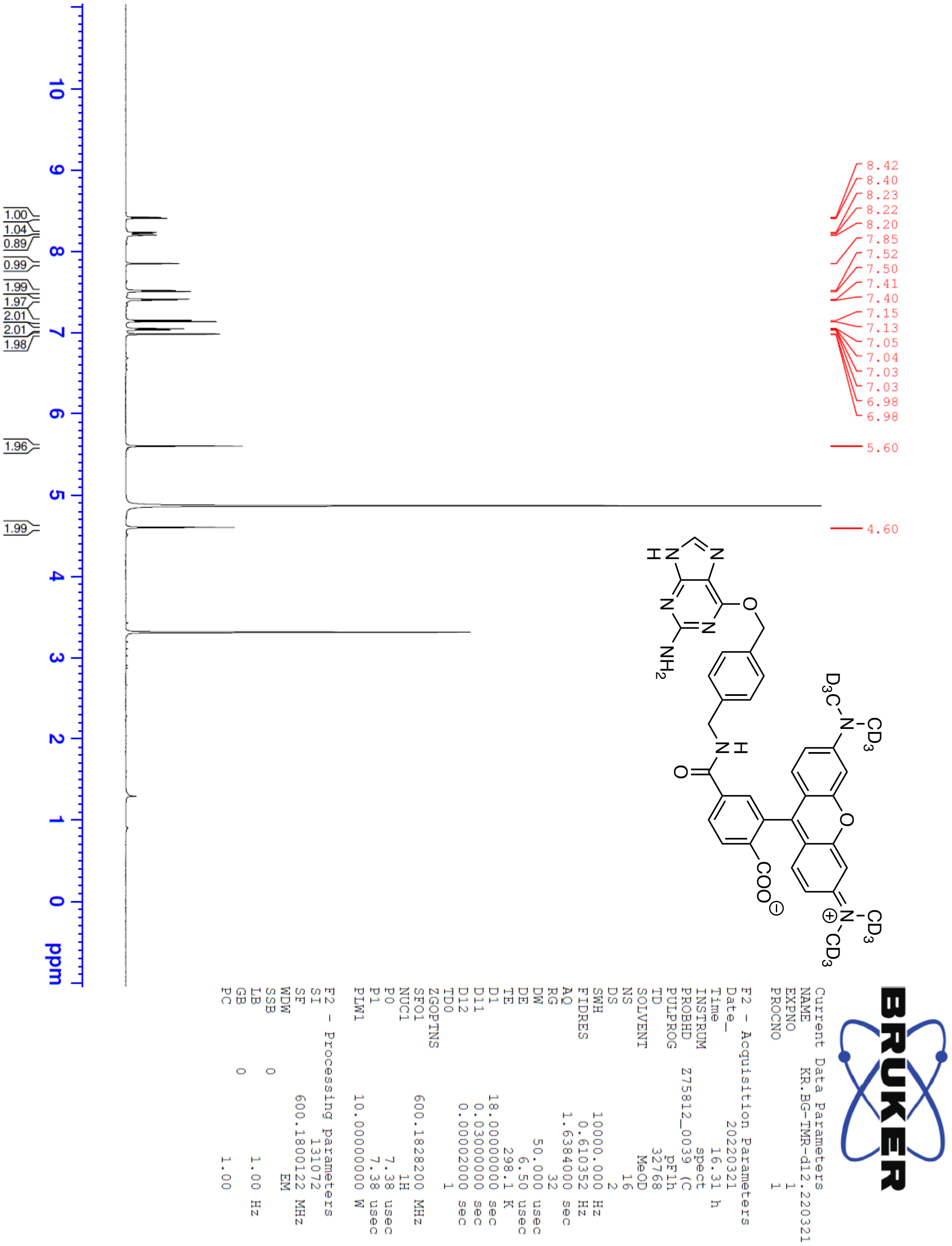

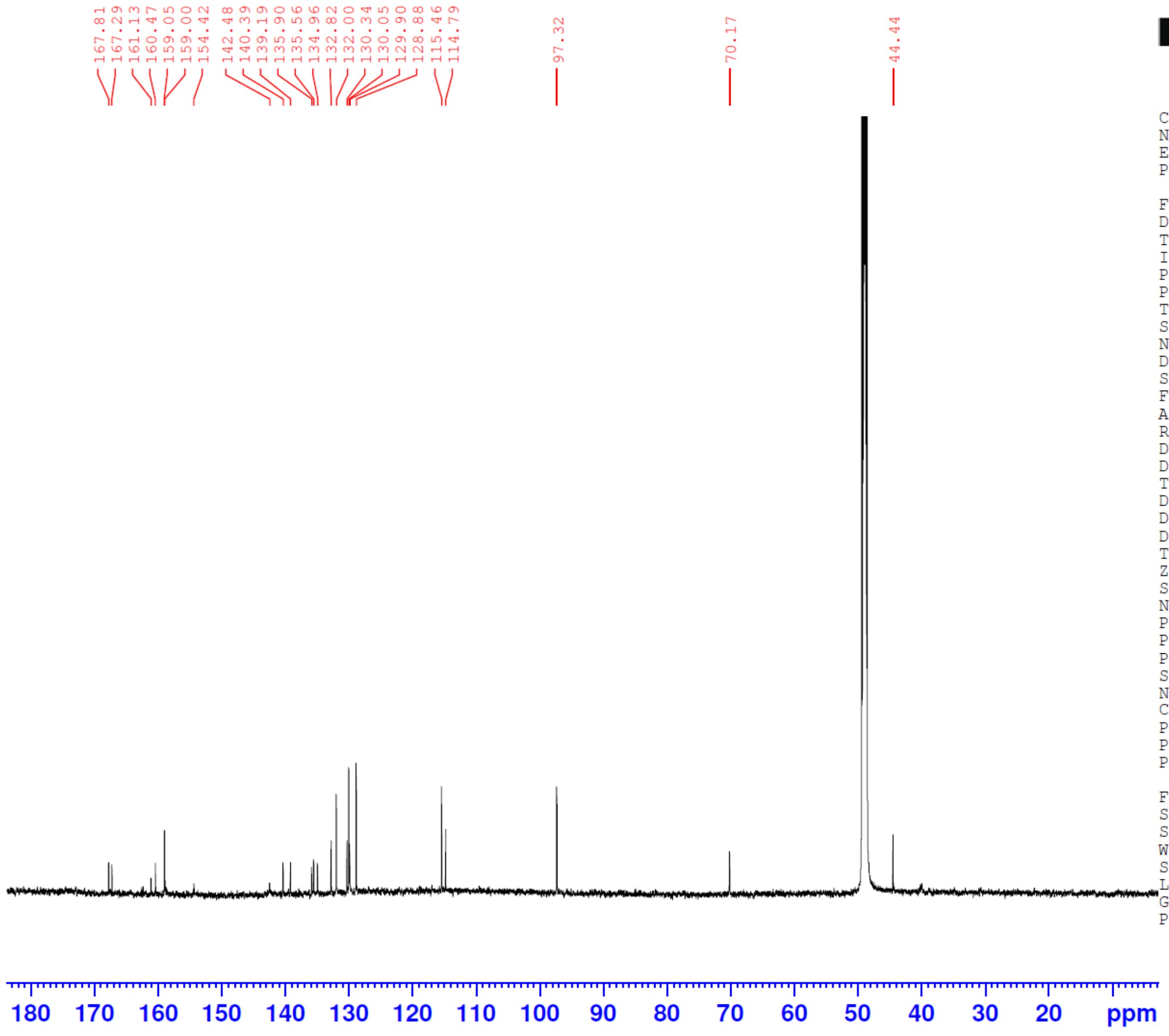


```
Current Data Parameters
NAME      KR.BG-TMR-d12.220321
EXPNO                   2
PROCNO                  1

F2 - Acquisition Parameters
Date_            20220321
Time                18.55 h
INSTRUM             spect
PROBHD   Z75812_0039 (C
PULPROG        MFhetero1d
TD                 131072
SOLVENT              MeOD
NS                   2048
DS                      2
SWH             31250.000 Hz
FIDRES           0.476837 Hz
AQ              2.0971520 sec
RG                    256
DW                 16.000 usec
DE                  20.00 usec
TE                  298.1 K
D1             2.00000000 sec
D11            0.03000000 sec
D12            0.00002000 sec
TD0                     1
ZGOPTNS        -Ddc -Dnoe
SFO1          150.9301310 MHz
NUC1                  13C
P0                  11.43 usec
P3                  11.43 usec
PLW1         120.23000336 W
SFO2          600.1828200 MHz
NUC2                   1H
CPDPRG[2          waltz16
PCPD2               80.00 usec
PLW2          10.00000000 W
PLW19          0.07620790 W

F2 - Processing parameters
SI                 131072
SF            150.9151787 MHz
WDW                    EM
SSB      0
LB                   3.00 Hz
GB       0
PC                   1.40
```

3.3. 2-(6-(Bis(methyl-d$_3$)amino)-3-(bis(methyl-d$_3$)iminio)-3*H*-xanthen-9-yl)-4-((2-(2-((6-chlorohexyl)oxy)ethoxy)ethyl)carbamoyl)benzoate (CA-TMR-d12)

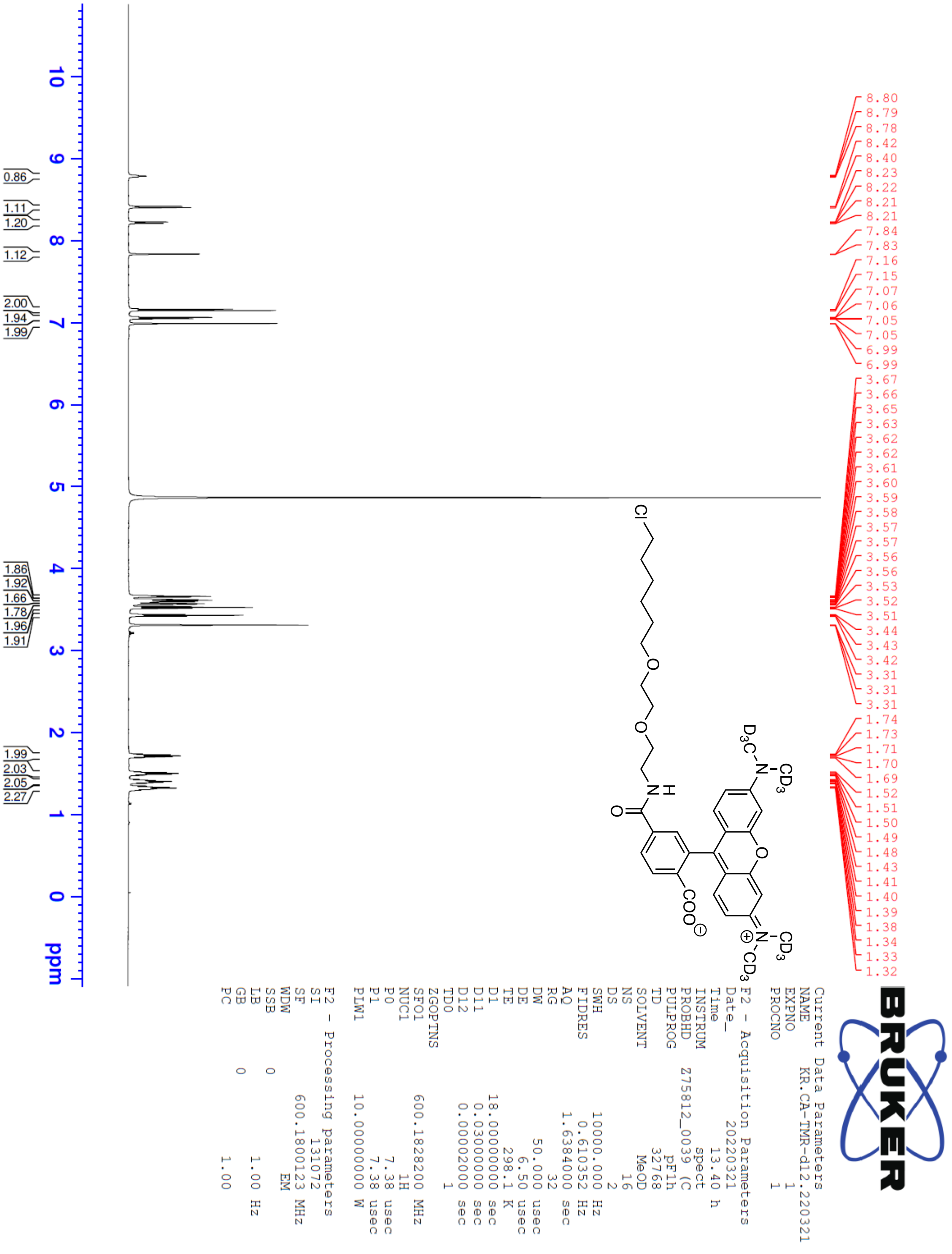

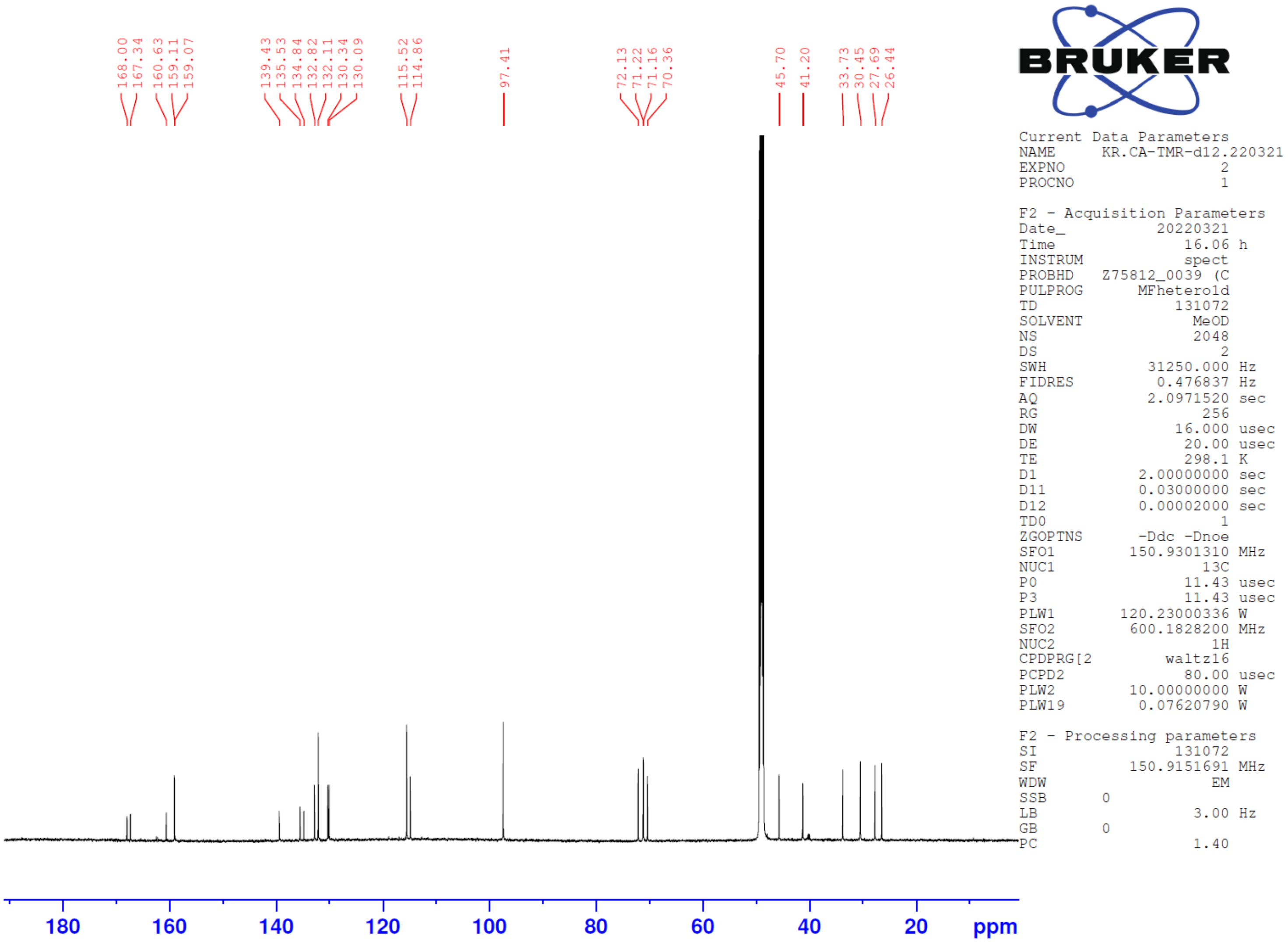
168.00
167.34
160.63
159.11
159.07
139.43
135.53
134.84
132.82
132.11
130.34
130.09
115.52
114.86
97.41
72.13
71.22
71.16
70.36
45.70
41.20
33.73
30.45
27.69
26.44
180
160
140
120
100
80
60
40
20
ppm
BRUKER
Current Data Parameters
NAME KR.CA-TMR-d12.220321
EXPNO 2
PROCNO 1
F2 - Acquisition Parameters
Date_ 20220321
Time 16.06 h
INSTRUM spect
PROBHD Z75812_0039 (C
PULPROG MFhetero1d
TD 131072
SOLVENT MeOD
NS 2048
DS 2
SWH 31250.000 Hz
FIDRES 0.476837 Hz
AQ 2.0971520 sec
RG 256
DW 16.000 usec
DE 20.00 usec
TE 298.1 K
D1 2.00000000 sec
D11 0.03000000 sec
D12 0.00002000 sec
TD0 1
ZGOPTNS -Ddc -Dnoe
SFO1 150.9301310 MHz
NUC1 13C
P0 11.43 usec
P3 11.43 usec
PLW1 120.23000336 W
SFO2 600.1828200 MHz
NUC2 1H
CPDPRG[2 waltz16
PCPD2 80.00 usec
PLW2 10.00000000 W
PLW19 0.07620790 W
F2 - Processing parameters
SI 131072
SF 150.9151691 MHz
WDW EM
SSB 0
LB 3.00 Hz
GB 0
PC 1.40

3.4. 3,7-Bis(bis(methyl-d$_3$)amino)-5,5-dimethyl-3'-oxo-3'*H*,5*H*-spiro[dibenzo[*b*,*e*]siline-10,1'-isobenzofuran]-6'-carboxylic acid (4)

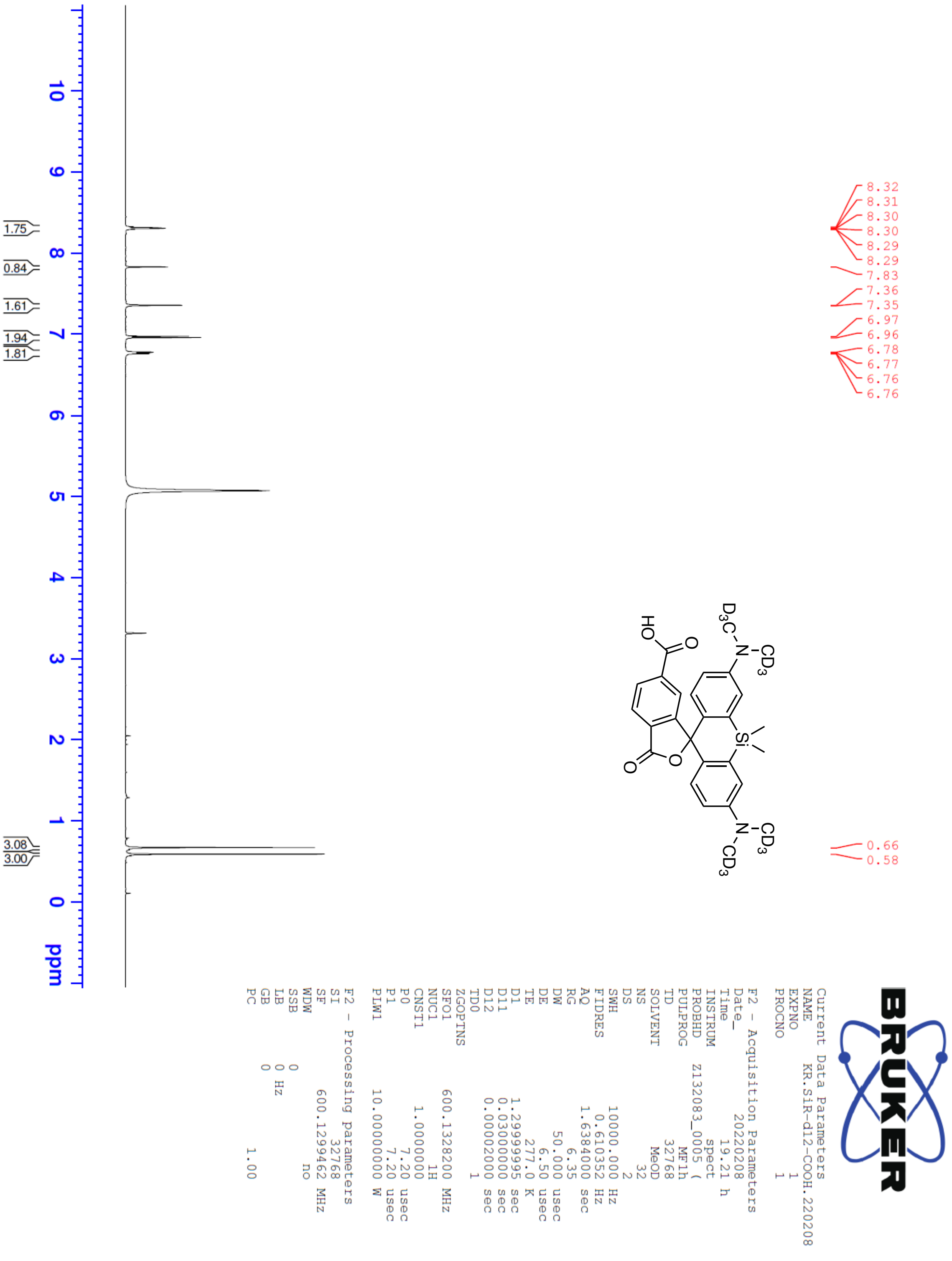

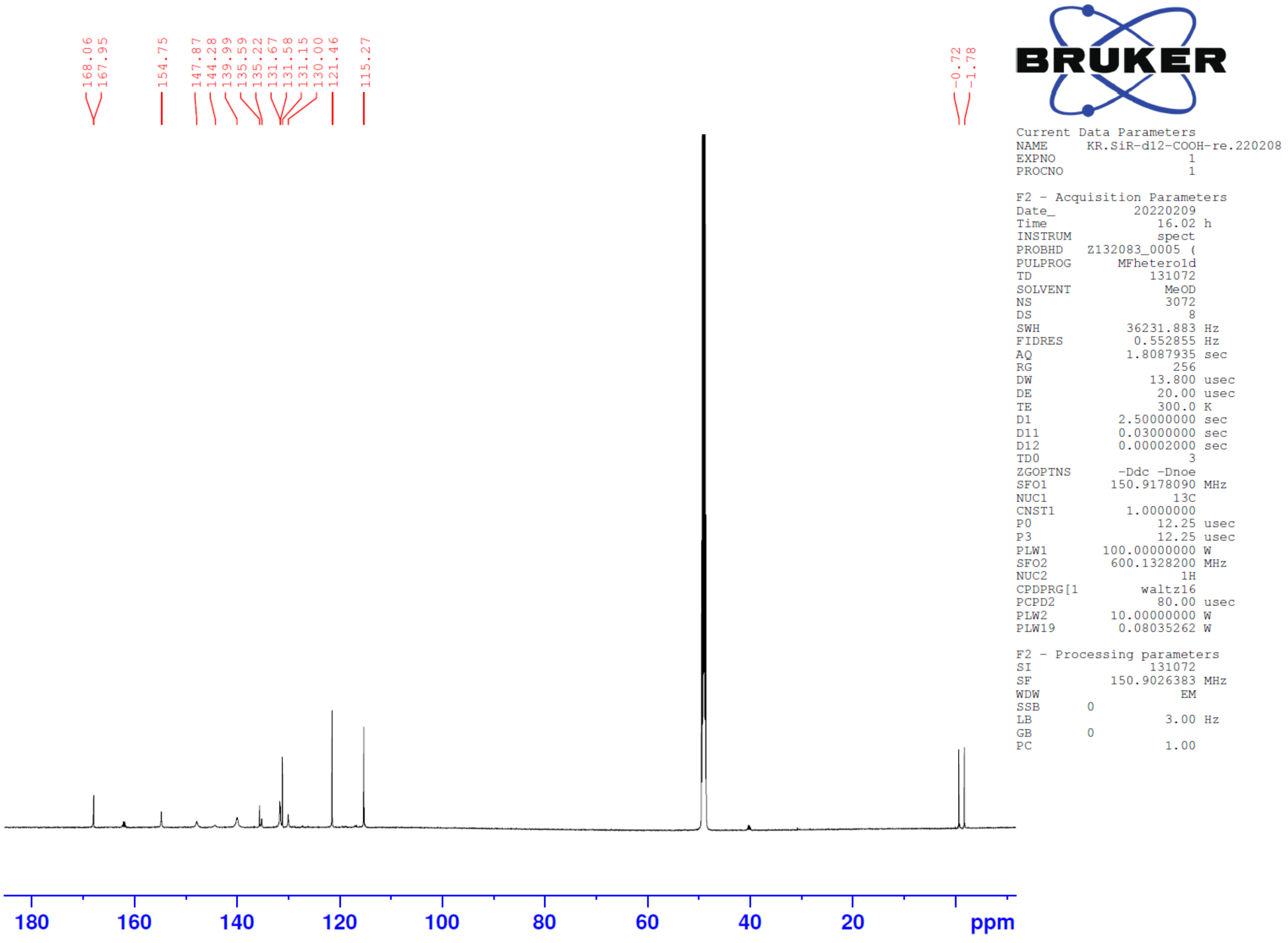
168.06
167.95
154.75
147.87
144.28
139.99
135.59
135.22
131.67
131.58
131.15
130.00
121.46
115.27
-0.72
-1.78
180
160
140
120
100
80
60
40
20
ppm
Current Data Parameters
NAME KR.SiR-d12-COOH-re.220208
EXPNO 1
PROCNO 1
F2 - Acquisition Parameters
Date_ 20220209
Time 16.02 h
INSTRUM spect
PROBHD Z132083_0005 (
PULPROG MFhetero1d
TD 131072
SOLVENT MeOD
NS 3072
DS 8
SWH 36231.883 Hz
FIDRES 0.552855 Hz
AQ 1.8087935 sec
RG 256
DW 13.800 usec
DE 20.00 usec
TE 300.0 K
D1 2.50000000 sec
D11 0.03000000 sec
D12 0.00002000 sec
TD0 3
ZGOPTNS -Ddc -Dnoe
SFO1 150.9178090 MHz
NUC1 13C
CNST1 1.0000000
P0 12.25 usec
P3 12.25 usec
PLW1 100.00000000 W
SFO2 600.1328200 MHz
NUC2 1H
CPDPRG[1 waltz16
PCPD2 80.00 usec
PLW2 10.00000000 W
PLW19 0.08035262 W
F2 - Processing parameters
SI 131072
SF 150.9026383 MHz
WDW EM
SSB 0
LB 3.00 Hz
GB 0
PC 1.00

3.5. 4-((4-(((2-Amino-9*H*-purin-6-yl)oxy)methyl)benzyl)carbamoyl)-2-(7-(bis(methyl-d_3)amino)-3-(bis(methyl-d_3)iminio)-5,5-dimethyl-3,5-dihydrodibenzo[*b,e*]silin-10-yl)benzoate (BG-SiR-d12)

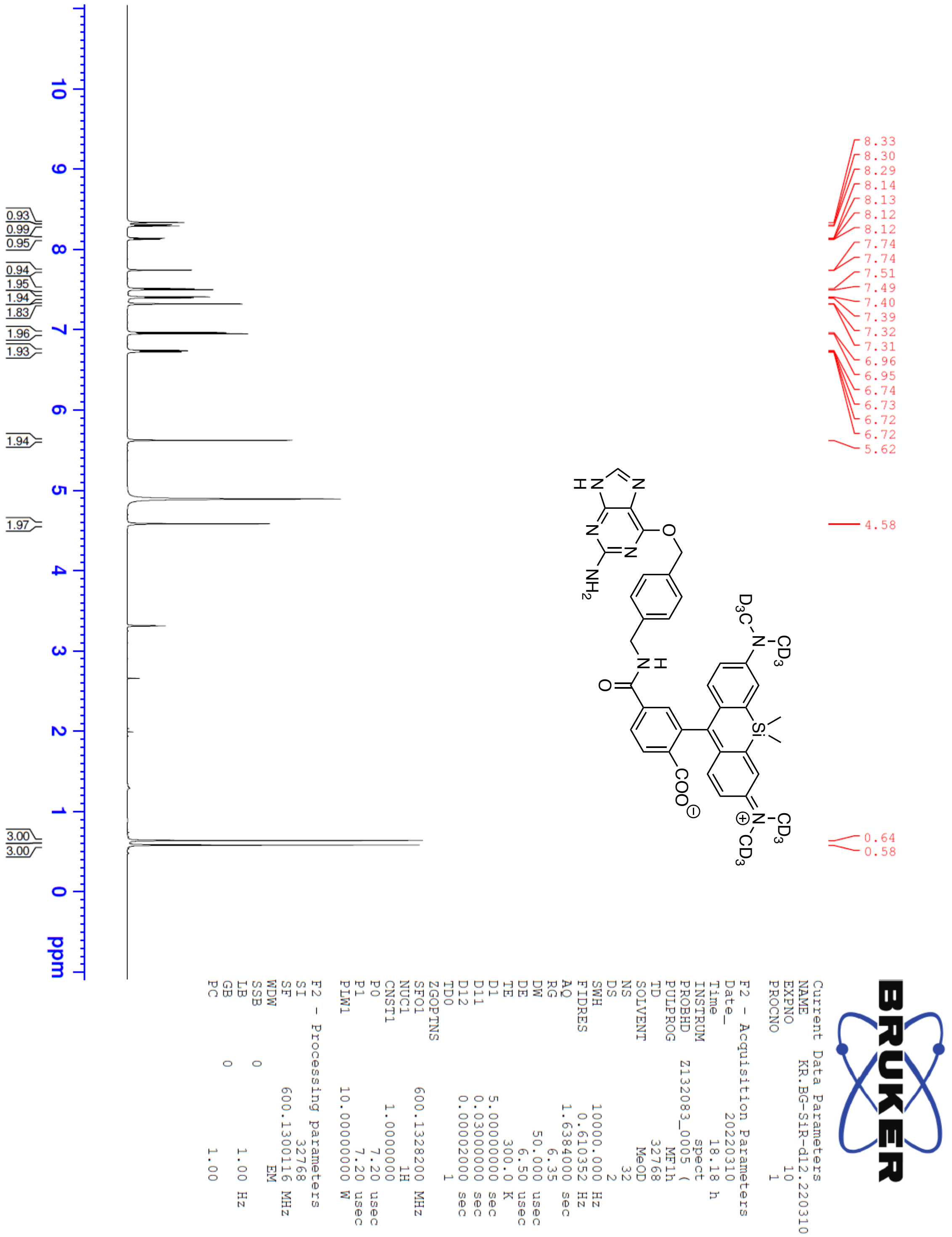

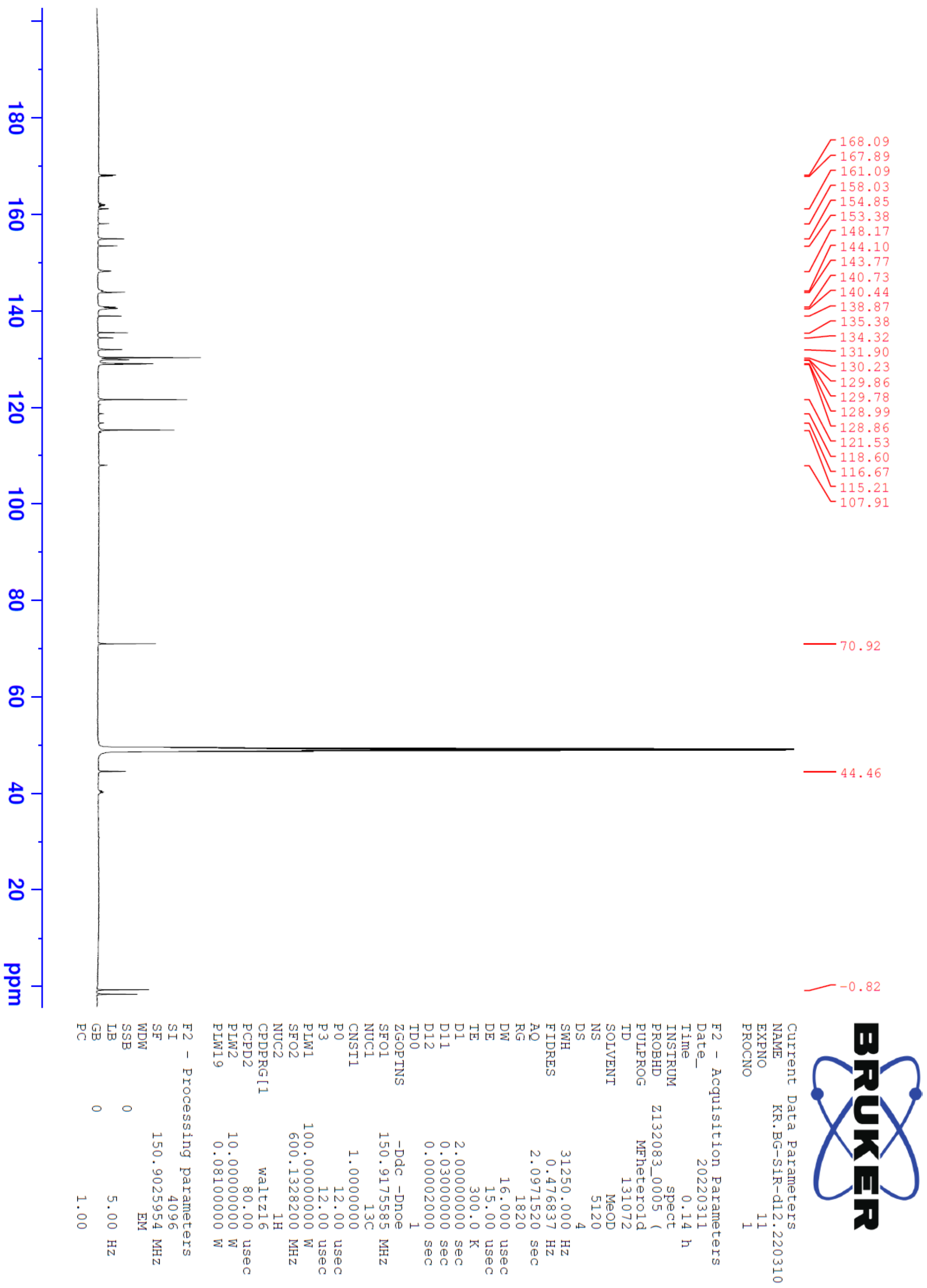
168.09
167.89
161.09
158.03
154.85
153.38
148.17
144.10
143.77
140.73
140.44
138.87
135.38
134.32
131.90
130.23
129.86
129.78
128.99
128.86
121.53
118.60
116.67
115.21
107.91
70.92
44.46
-0.82
180
160
140
120
100
80
60
40
20
ppm
BRUKER
Current Data Parameters
NAME KR.BG-SiR-d12.220310
EXPNO 11
PROCNO 1
F2 - Acquisition Parameters
Date_ 20220311
Time 0.14 h
INSTRUM spect
PROBHD Z132083_0005 (
PULPROG MFhetero1d
TD 131072
SOLVENT MeOD
NS 5120
DS 4
SWH 31250.000 Hz
FIDRES 0.476837 Hz
AQ 2.0971520 sec
RG 1820
DW 16.000 usec
DE 15.00 usec
TE 300.0 K
D1 2.00000000 sec
D11 0.03000000 sec
D12 0.00002000 sec
TD0 1
ZGOPTNS -Ddc -Dnoe
SFO1 150.9175585 MHz
NUC1 13C
CNST1 1.000000
P0 12.00 usec
P3 12.00 usec
PLW1 100.00000000 W
SFO2 600.1328200 MHz
NUC2 1H
CPDPRG[1 waltz16
PCPD2 80.00 usec
PLW2 10.00000000 W
PLW19 0.08100000 W
F2 - Processing parameters
SI 4096
SF 150.9025954 MHz
WDW EM
SSB 0
LB 5.00 Hz
GB 0
PC 1.00

3.6. 2-(7-(Bis(methyl-d_3)amino)-3-(bis(methyl-d_3)iminio)-5,5-dimethyl-3,5-dihydrodibenzo[*b,e*]silin-10-yl)-4-((2-(2-((6-chlorohexyl)oxy)ethoxy)ethyl)carbamoyl)benzoate (CA-SiR-d12)

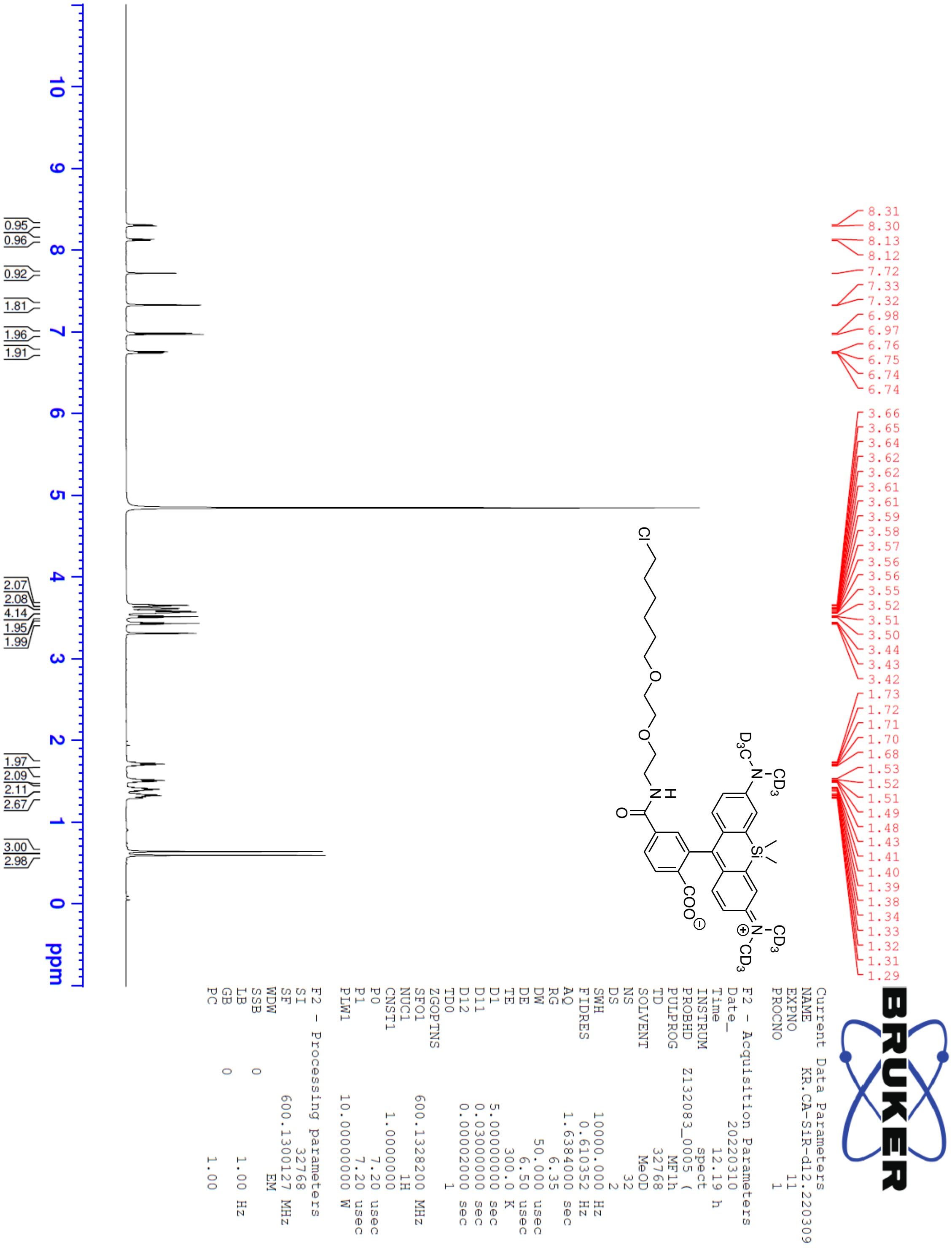

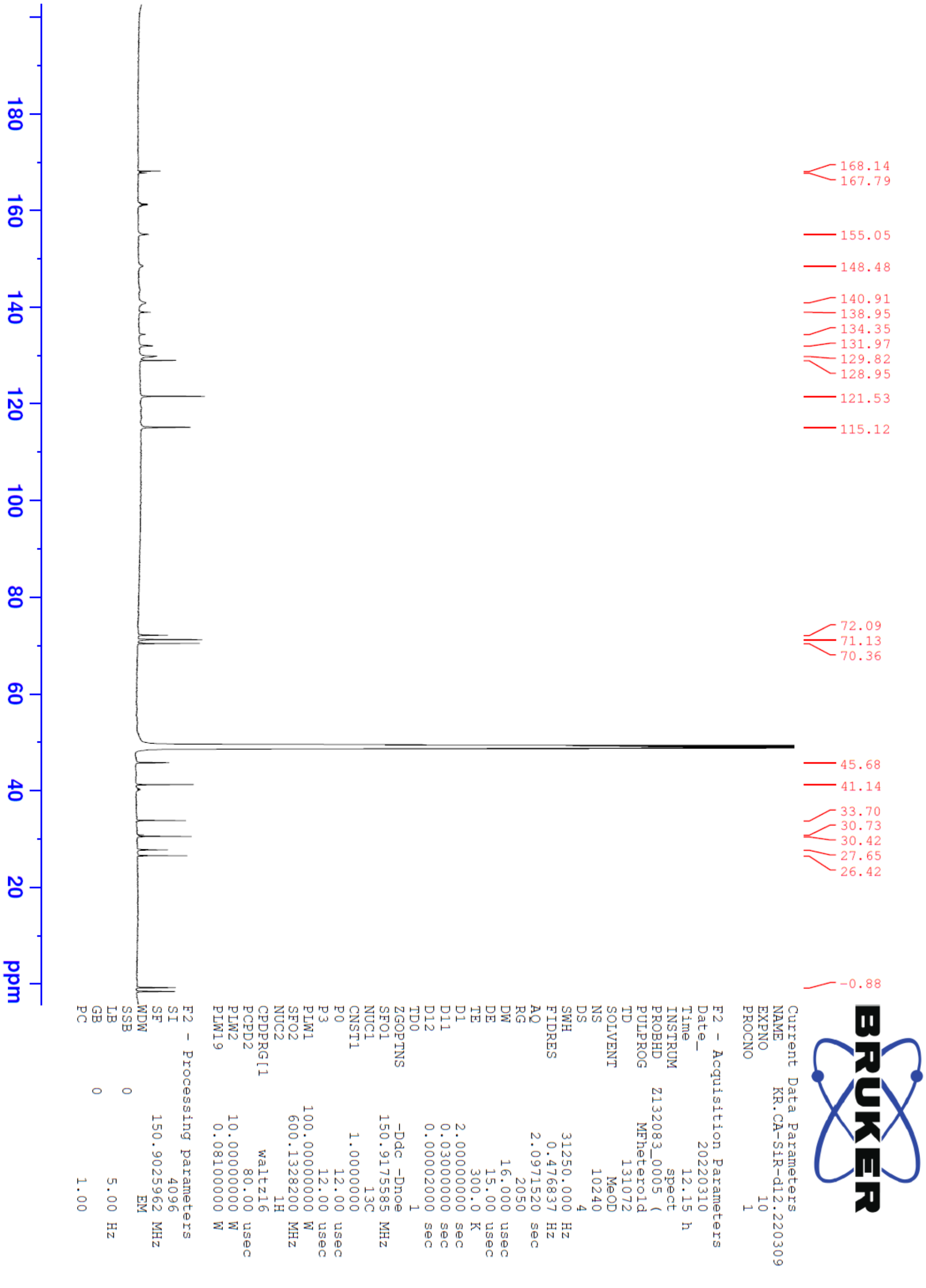
168.14
167.79
155.05
148.48
140.91
138.95
134.35
131.97
129.82
128.95
121.53
115.12
72.09
71.13
70.36
45.68
41.14
33.70
30.73
30.42
27.65
26.42
-0.88
180
160
140
120
100
80
60
40
20
ppm
BRUKER
Current Data Parameters
NAME KR.CA-SiR-d12.220309
EXPNO 10
PROCNO 1
F2 - Acquisition Parameters
Date_ 20220310
Time 12.15 h
INSTRUM spect
PROBHD Z132083_0005 (
PULPROG MFhetero1d
TD 131072
SOLVENT MeOD
NS 10240
DS 4
SWH 31250.000 Hz
FIDRES 0.476837 Hz
AQ 2.0971520 sec
RG 2050
DW 16.000 usec
DE 15.00 usec
TE 300.0 K
D1 2.00000000 sec
D11 0.03000000 sec
D12 0.00002000 sec
TD0 1
ZGOPTNS -Ddc -Dnoe
SFO1 150.9175585 MHz
NUC1 13C
CNST1 1.0000000
P0 12.00 usec
P3 12.00 usec
PLW1 100.00000000 W
SFO2 600.1328200 MHz
NUC2 1H
CPDPRG[1 waltz16
PCPD2 80.00 usec
PLW2 10.00000000 W
PLW19 0.08100000 W
F2 - Processing parameters
SI 4096
SF 150.9025962 MHz
WDW EM
SSB 0
LB 5.00 Hz
GB 0
PC 1.00

3.7. 2-(6-(Bis(methyl-d$_3$)amino)-3-(bis(methyl-d$_3$)iminio)-3*H*-xanthen-9-yl)-4-((2-(2,5-dioxo-2,5-dihydro-1*H*-pyrrol-1-yl)ethyl)carbamoyl)benzoate (Mal-TMR-d12)

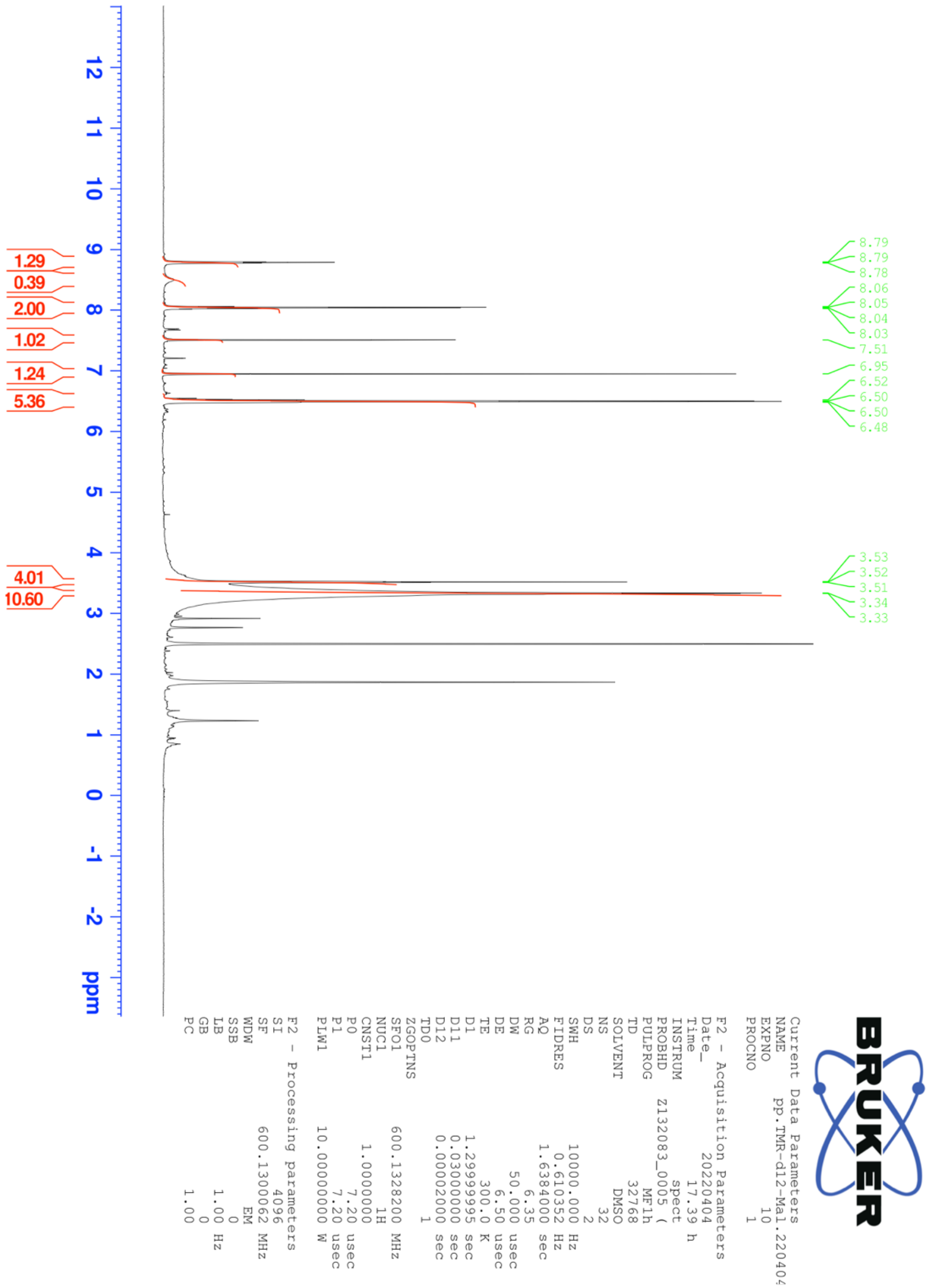

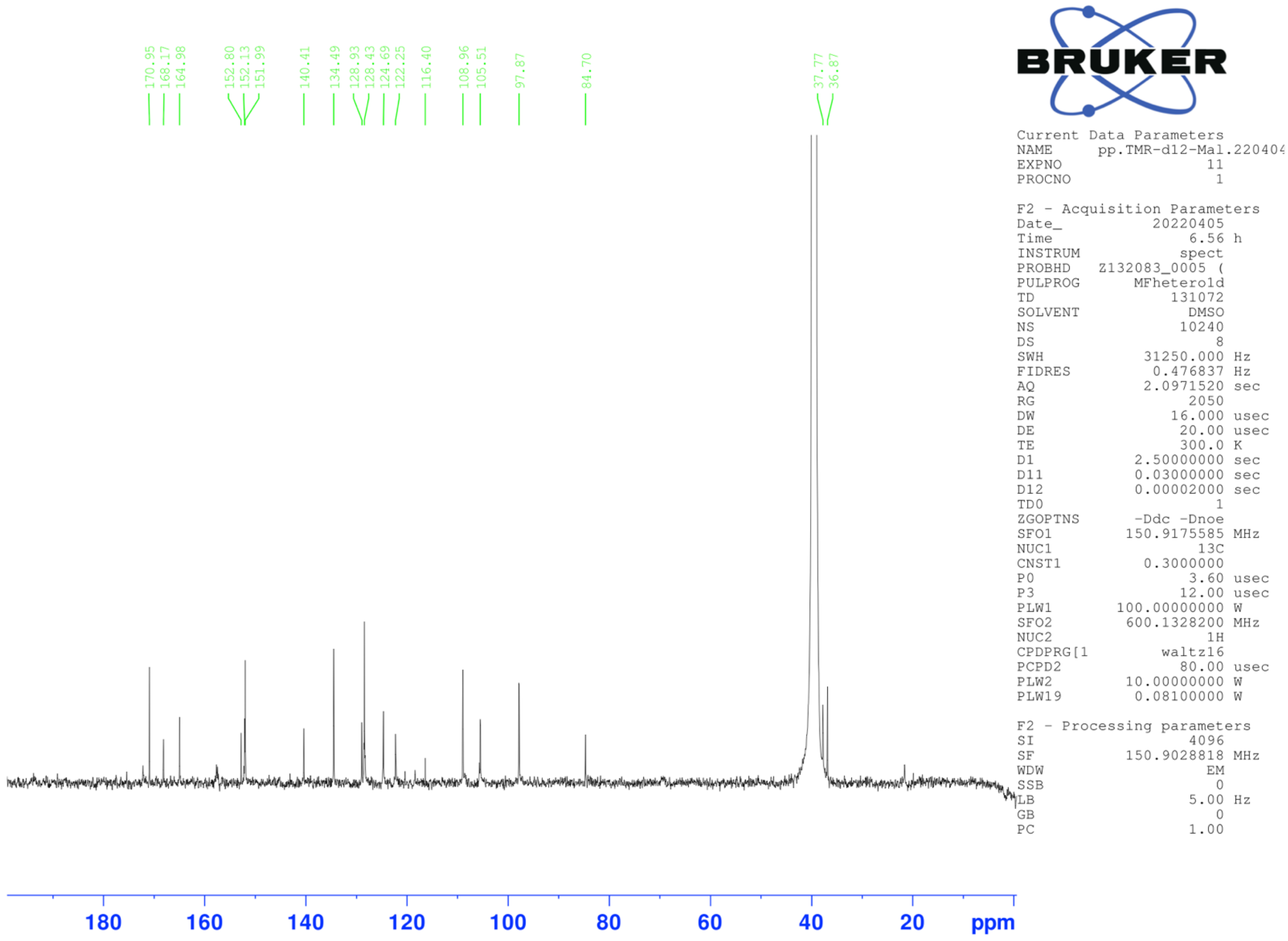
170.95
168.17
164.98
152.80
152.13
151.99
140.41
134.49
128.93
128.43
124.69
122.25
116.40
108.96
105.51
97.87
84.70
37.77
36.87
180
160
140
120
100
80
60
40
20
ppm
BRUKER
Current Data Parameters
NAME pp.TMR-d12-Mal.220404
EXPNO 11
PROCNO 1
F2 - Acquisition Parameters
Date_ 20220405
Time 6.56 h
INSTRUM spect
PROBHD Z132083_0005 (
PULPROG MFhetero1d
TD 131072
SOLVENT DMSO
NS 10240
DS 8
SWH 31250.000 Hz
FIDRES 0.476837 Hz
AQ 2.0971520 sec
RG 2050
DW 16.000 usec
DE 20.00 usec
TE 300.0 K
D1 2.50000000 sec
D11 0.03000000 sec
D12 0.00002000 sec
TD0 1
ZGOPTNS -Ddc -Dnoe
SFO1 150.9175585 MHz
NUC1 13C
CNST1 0.3000000
P0 3.60 usec
P3 12.00 usec
PLW1 100.00000000 W
SFO2 600.1328200 MHz
NUC2 1H
CPDPRG[1 waltz16
PCPD2 80.00 usec
PLW2 10.00000000 W
PLW19 0.08100000 W
F2 - Processing parameters
SI 4096
SF 150.9028818 MHz
WDW EM
SSB 0
LB 5.00 Hz
GB 0
PC 1.00

3.8. 2-(7-(bis(methyl-d3)amino)-3-(bis(methyl-d3)iminio)-5,5-dimethyl-3,5-dihydrodibenzo[b,e]silin-10-yl)-4-((2-(2,5-dioxo-2,5-dihydro-1H-pyrrol-1-yl)ethyl)carbamoyl)benzoate (Mal-SiR-d12)

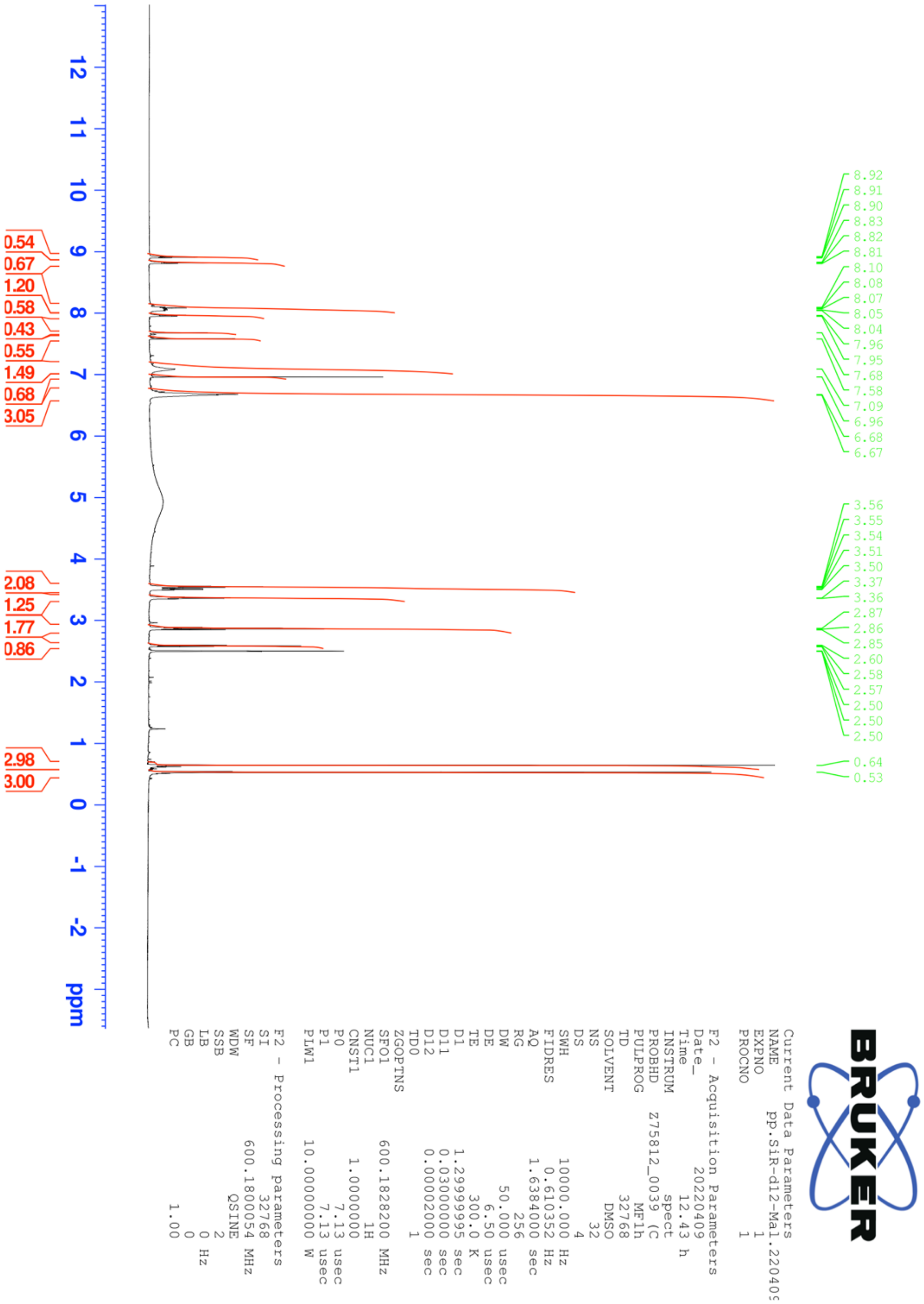

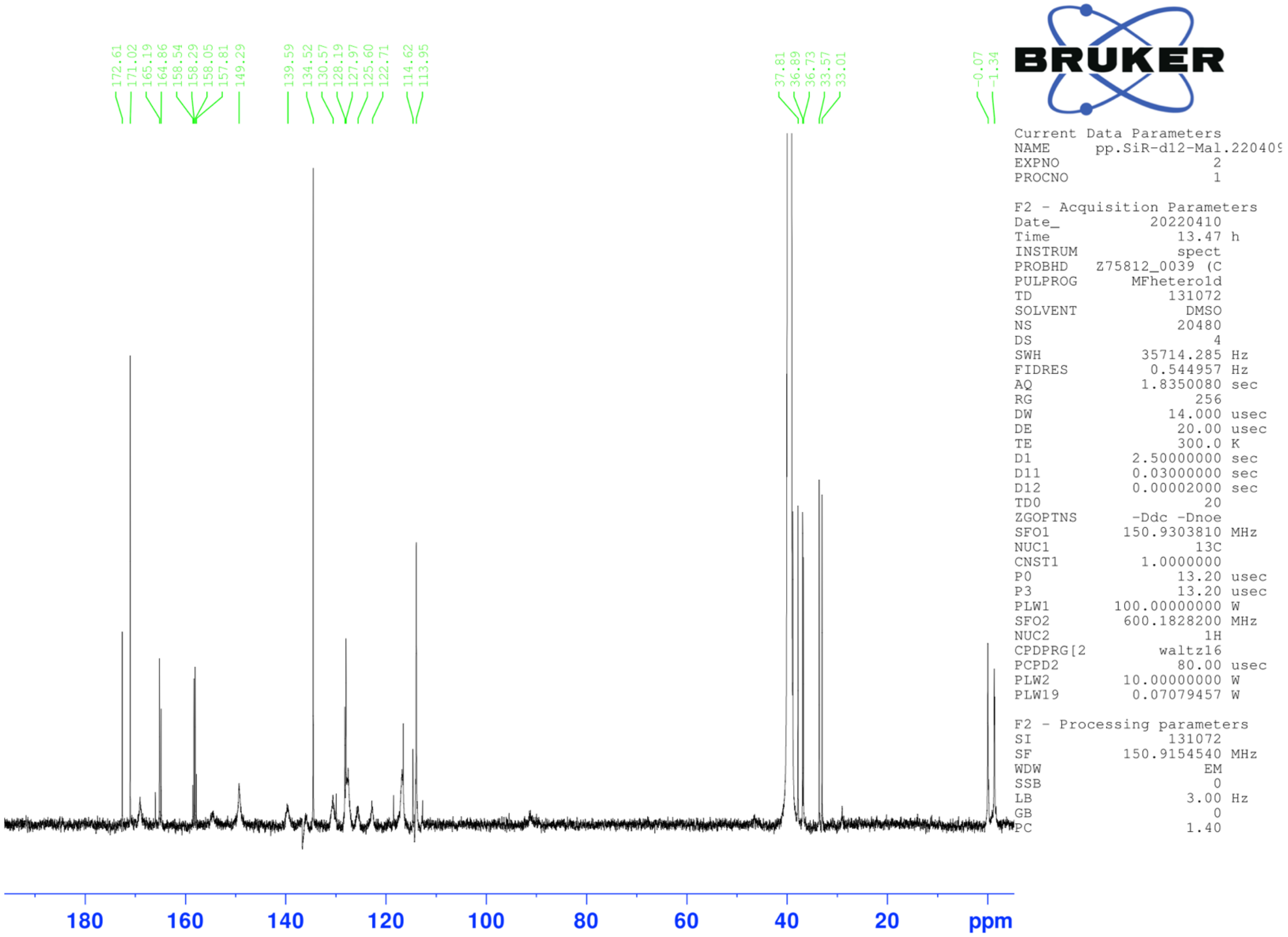

172.61
171.02
165.19
164.86
158.54
158.29
158.05
157.81
149.29
139.59
134.52
130.57
128.19
127.97
125.60
122.71
114.62
113.95
37.81
36.89
36.73
33.57
33.01
-0.07
-1.34
BRUKER
Current Data Parameters
NAME pp.SiR-d12-Mal.22040
EXPNO 2
PROCNO 1
F2 - Acquisition Parameters
Date_ 20220410
Time 13.47 h
INSTRUM spect
PROBHD Z75812_0039 (C
PULPROG MFhetero1d
TD 131072
SOLVENT DMSO
NS 20480
DS 4
SWH 35714.285 Hz
FIDRES 0.544957 Hz
AQ 1.8350080 sec
RG 256
DW 14.000 usec
DE 20.00 usec
TE 300.0 K
D1 2.50000000 sec
D11 0.03000000 sec
D12 0.00002000 sec
TD0 20
ZGOPTNS -Ddc -Dnoe
SFO1 150.9303810 MHz
NUC1 13C
CNST1 1.0000000
P0 13.20 usec
P3 13.20 usec
PLW1 100.00000000 W
SFO2 600.1828200 MHz
NUC2 1H
CPDPRG[2 waltz16
PCPD2 80.00 usec
PLW2 10.00000000 W
PLW19 0.07079457 W
F2 - Processing parameters
SI 131072
SF 150.9154540 MHz
WDW EM
SSB 0
LB 3.00 Hz
GB 0
PC 1.40
180
160
140
120
100
80
60
40
20
ppm

3.9. 7-(Dimethylamino)-4-methyl-2*H*-chromen-2-one (Coumarin 461)

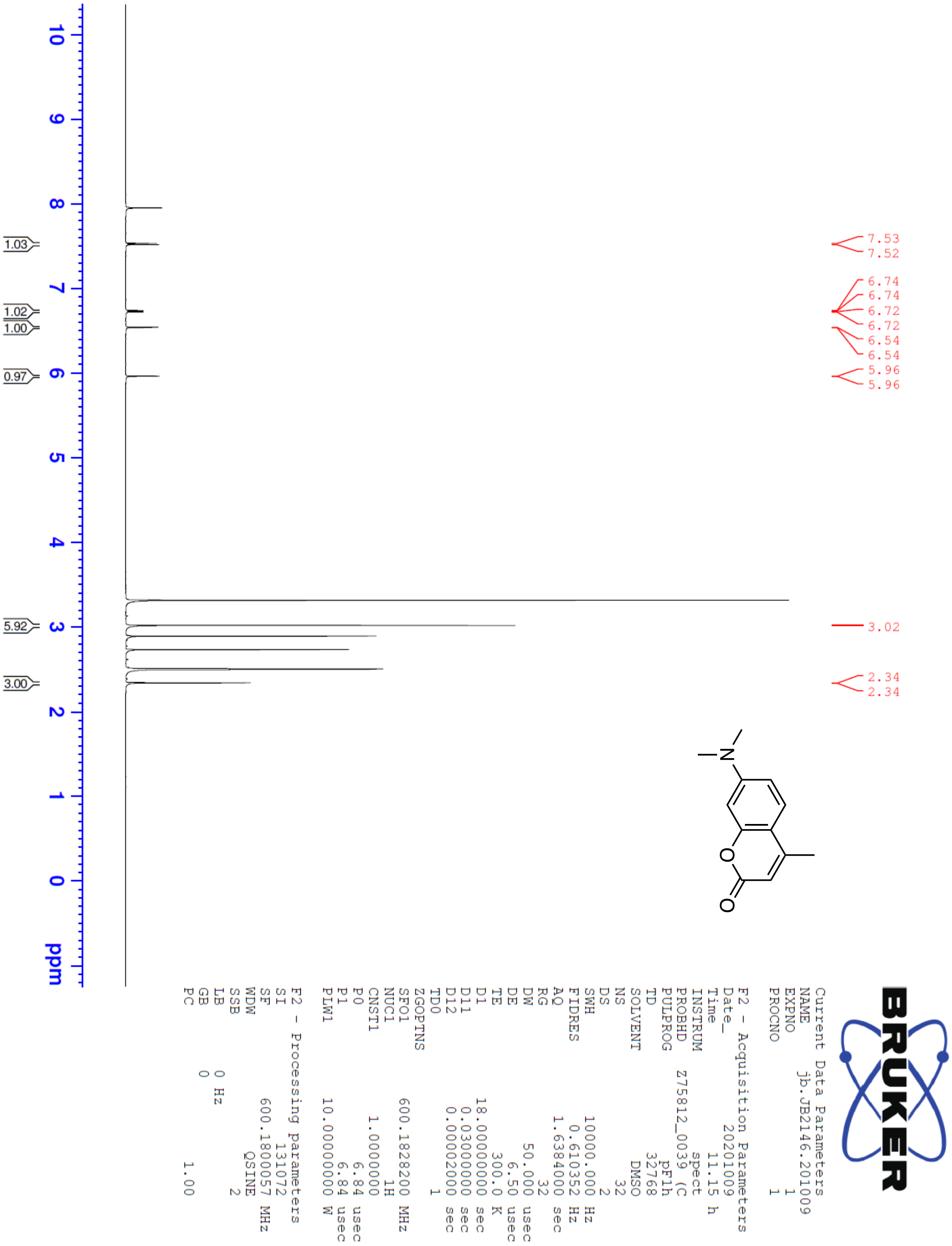

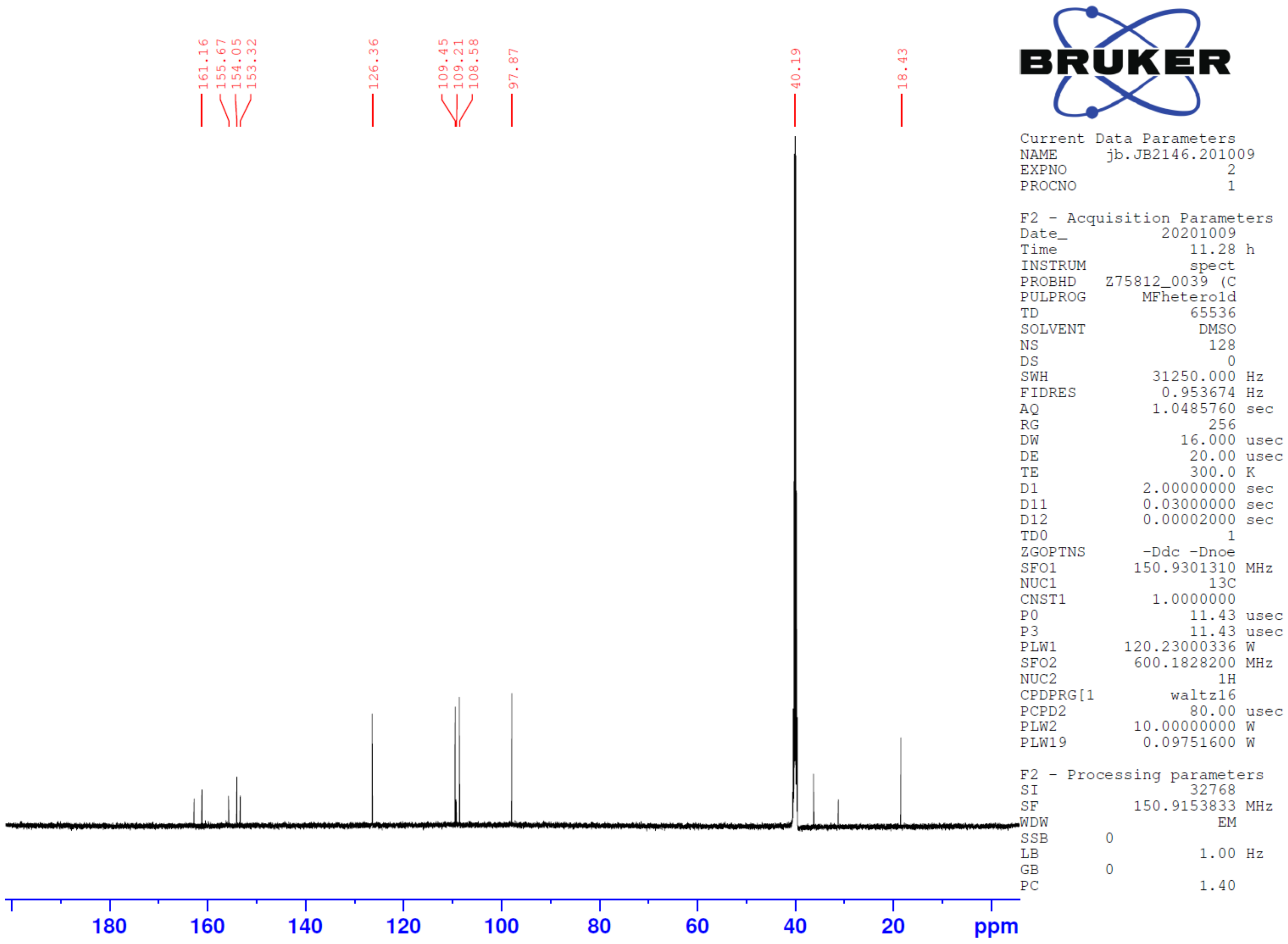
161.16
155.67
154.05
153.32
126.36
109.45
109.21
108.58
97.87
40.19
18.43
BRUKER
Current Data Parameters
NAME jb.JB2146.201009
EXPNO 2
PROCNO 1
F2 - Acquisition Parameters
Date_ 20201009
Time 11.28 h
INSTRUM spect
PROBHD Z75812_0039 (C
PULPROG MFhetero1d
TD 65536
SOLVENT DMSO
NS 128
DS 0
SWH 31250.000 Hz
FIDRES 0.953674 Hz
AQ 1.0485760 sec
RG 256
DW 16.000 usec
DE 20.00 usec
TE 300.0 K
D1 2.00000000 sec
D11 0.03000000 sec
D12 0.00002000 sec
TD0 1
ZGOPTNS -Ddc -Dnoe
SFO1 150.9301310 MHz
NUC1 13C
CNST1 1.0000000
P0 11.43 usec
P3 11.43 usec
PLW1 120.23000336 W
SFO2 600.1828200 MHz
NUC2 1H
CPDPRG[1 waltz16
PCPD2 80.00 usec
PLW2 10.00000000 W
PLW19 0.09751600 W
F2 - Processing parameters
SI 32768
SF 150.9153833 MHz
WDW EM
SSB 0
LB 1.00 Hz
GB 0
PC 1.40
180
160
140
120
100
80
60
40
20
ppm

3.10. 4-Methyl-7-((methyl-d3)amino)-2*H*-chromen-2-one (6)

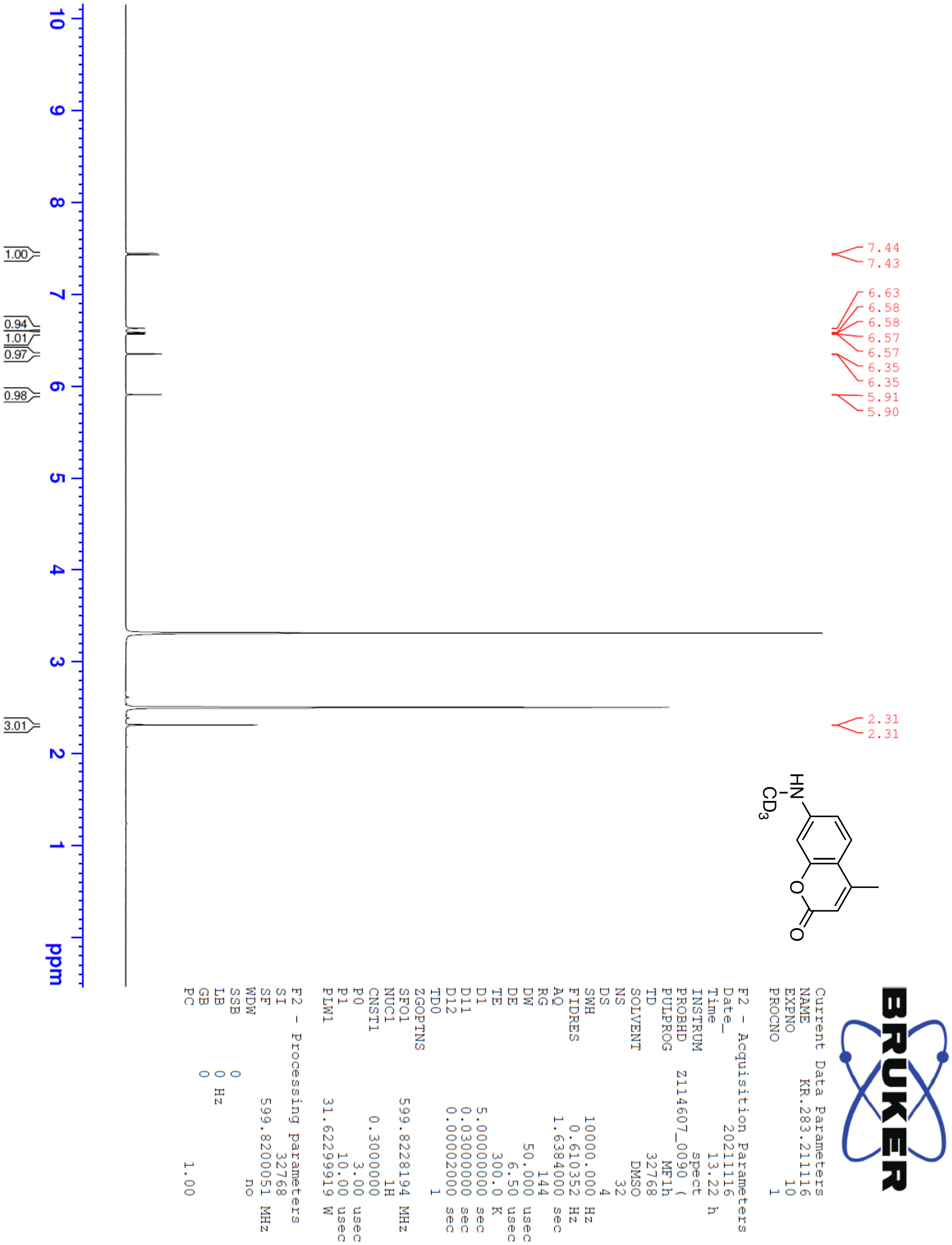

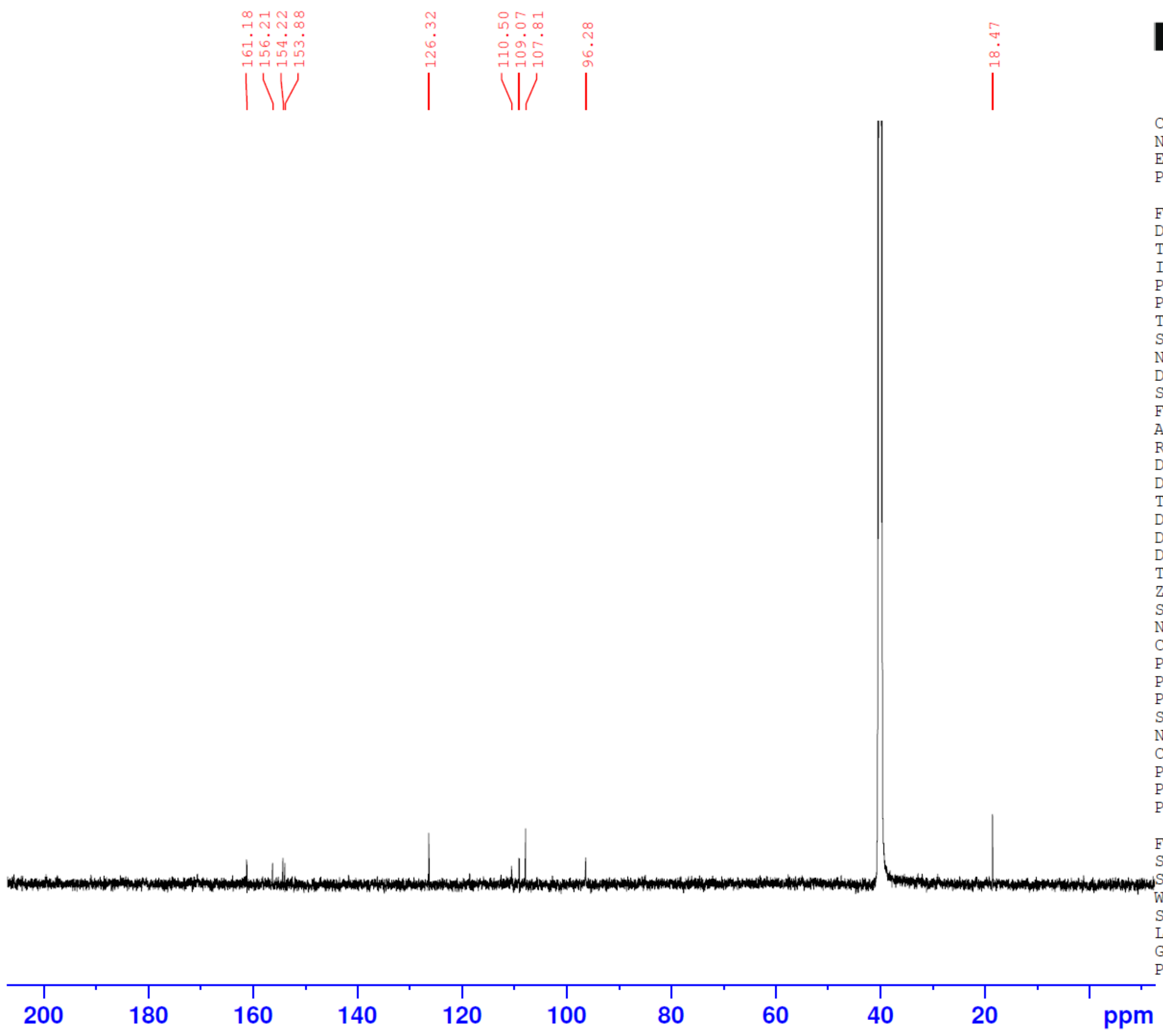

Current Data Parameters

NAME	KR.283.211116	
EXPNO	11	
PROCNO	1	

F2 - Acquisition Parameters

Date_	20211116	
Time	23.57	h
INSTRUM	spect	
PROBHD	Z114607_0090 (	
PULPROG	MFhetero1d	
TD	131072	
SOLVENT	DMSO	
NS	4096	
DS	4	
SWH	45454.547	Hz
FIDRES	0.693581	Hz
AQ	1.4417920	sec
RG	2050	
DW	11.000	usec
DE	6.50	usec
TE	300.0	K
D1	2.50000000	sec
D11	0.03000000	sec
D12	0.00002000	sec
TD0	1	
ZGOPTNS	-Ddc -Dnoe	
SFO1	150.8398557	MHz
NUC1	13C	
CNST1	0.3000000	
P0	2.58	usec
P3	8.60	usec
PLW1	158.49000549	W
SFO2	599.8228182	MHz
NUC2	1H	
CPDPRG[1	waltz16	
PCPD2	80.00	usec
PLW2	31.62299919	W
PLW19	0.49410999	W

F2 - Processing parameters

SI	131072	
SF	150.8248634	MHz
WDW	EM	
SSB	0	
LB	3.00	Hz
GB	0	
PC	1.00	

3.11. 4-Methyl-7-(methyl(methyl-d3)amino)-2*H*-chromen-2-one (Coumarin 461-d3)

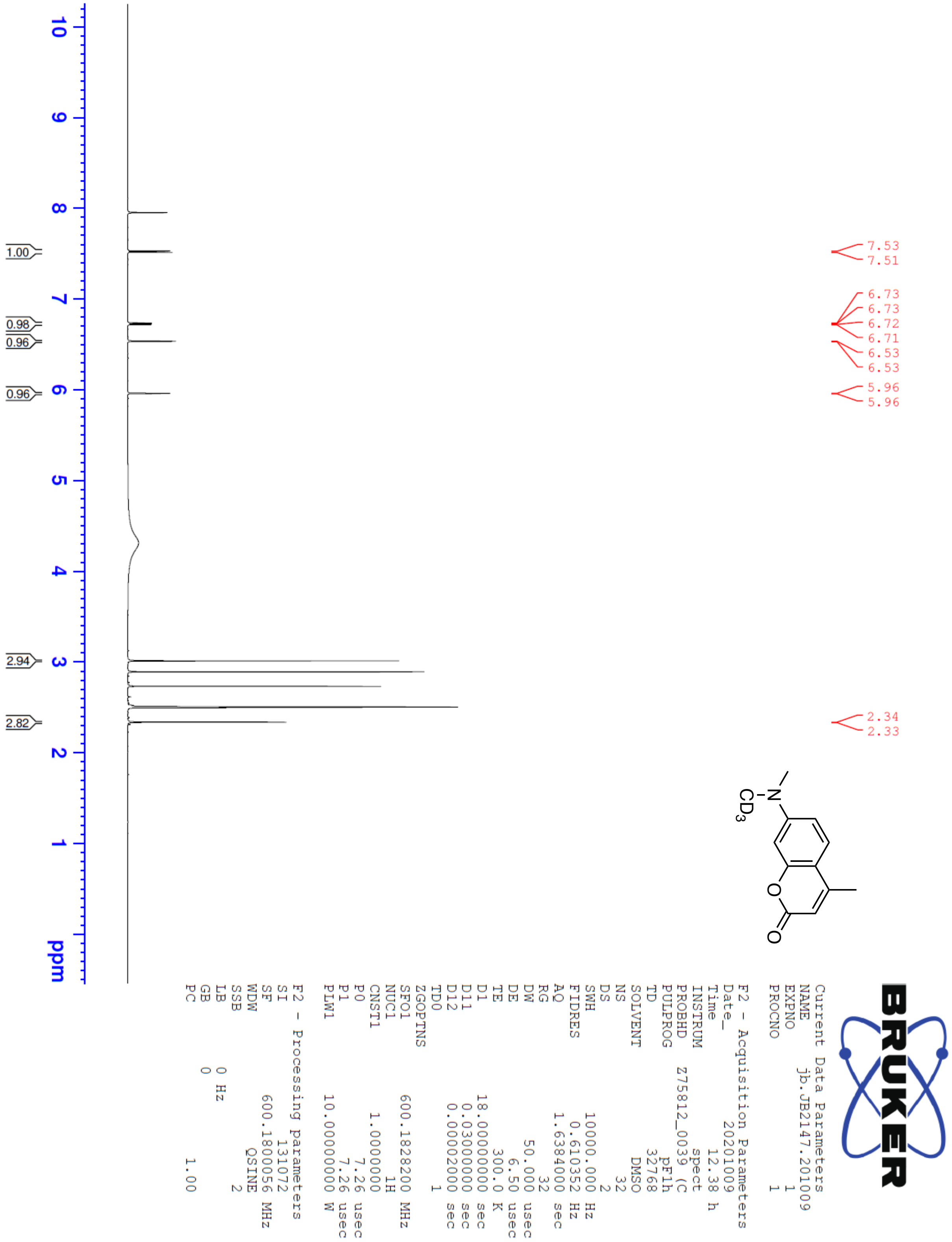

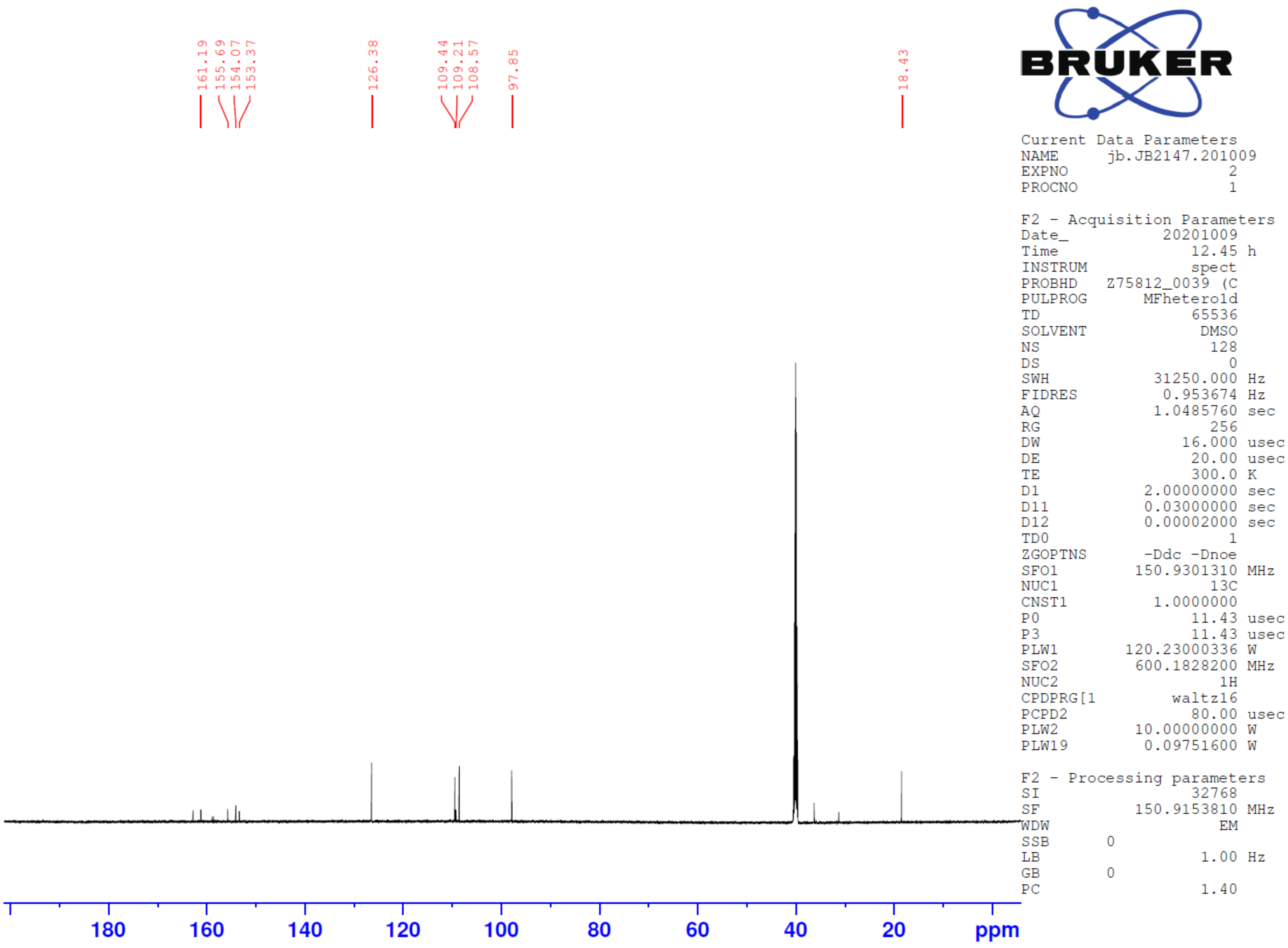
161.19
155.69
154.07
153.37
126.38
109.44
109.21
108.57
97.85
18.43
180
160
140
120
100
80
60
40
20
ppm
BRUKER
Current Data Parameters
NAME jb.JB2147.201009
EXPNO 2
PROCNO 1
F2 - Acquisition Parameters
Date_ 20201009
Time 12.45 h
INSTRUM spect
PROBHD Z75812_0039 (C
PULPROG MFhetero1d
TD 65536
SOLVENT DMSO
NS 128
DS 0
SWH 31250.000 Hz
FIDRES 0.953674 Hz
AQ 1.0485760 sec
RG 256
DW 16.000 usec
DE 20.00 usec
TE 300.0 K
D1 2.00000000 sec
D11 0.03000000 sec
D12 0.00002000 sec
TD0 1
ZGOPTNS -Ddc -Dnoe
SFO1 150.9301310 MHz
NUC1 13C
CNST1 1.0000000
P0 11.43 usec
P3 11.43 usec
PLW1 120.23000336 W
SFO2 600.1828200 MHz
NUC2 1H
CPDPRG[1 waltz16
PCPD2 80.00 usec
PLW2 10.00000000 W
PLW19 0.09751600 W
F2 - Processing parameters
SI 32768
SF 150.9153810 MHz
WDW EM
SSB 0
LB 1.00 Hz
GB 0
PC 1.40

3.12. 7-(Bis(methyl-d3)amino)-4-methyl-2*H*-chromen-2-one (Coumarin 461-d6)

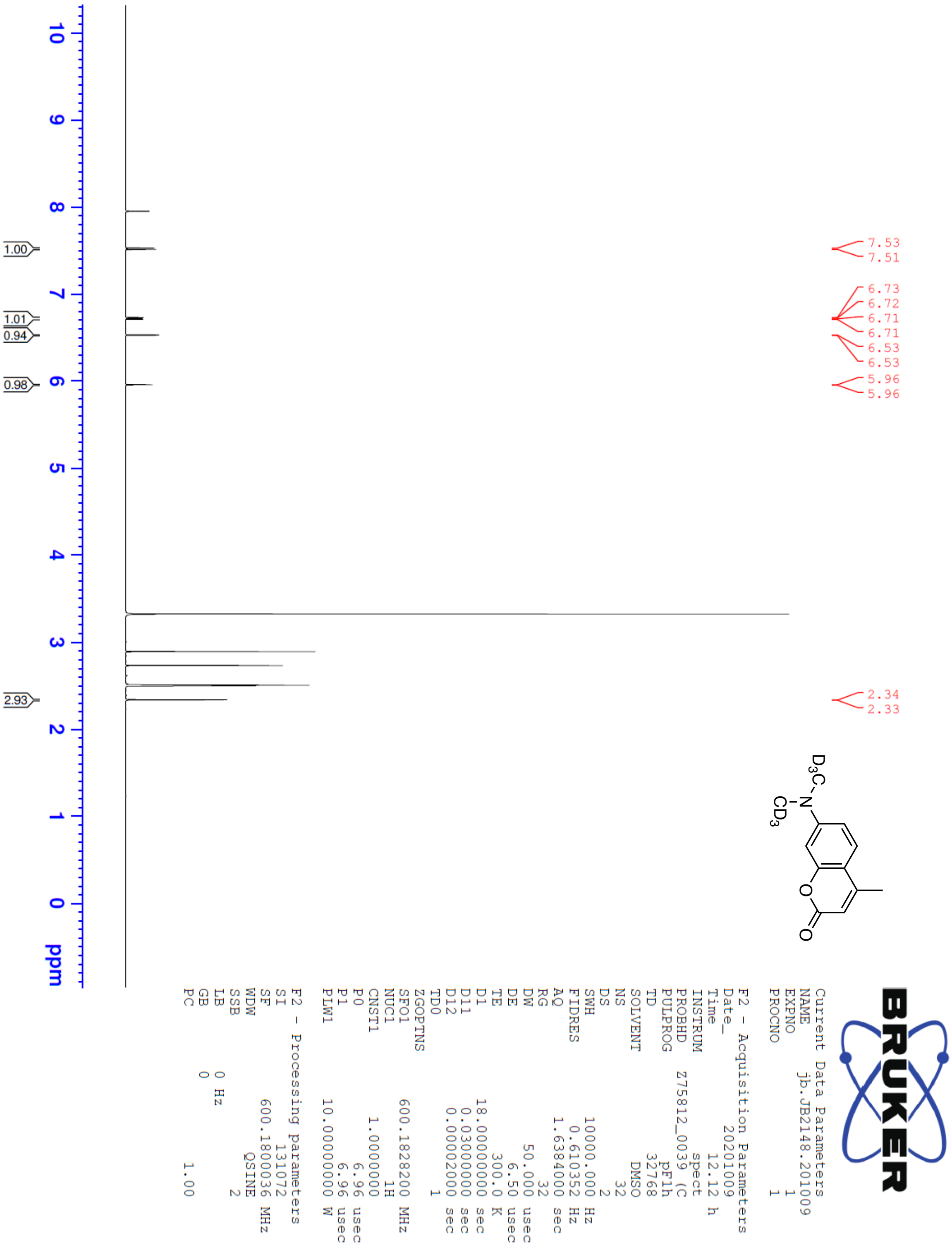

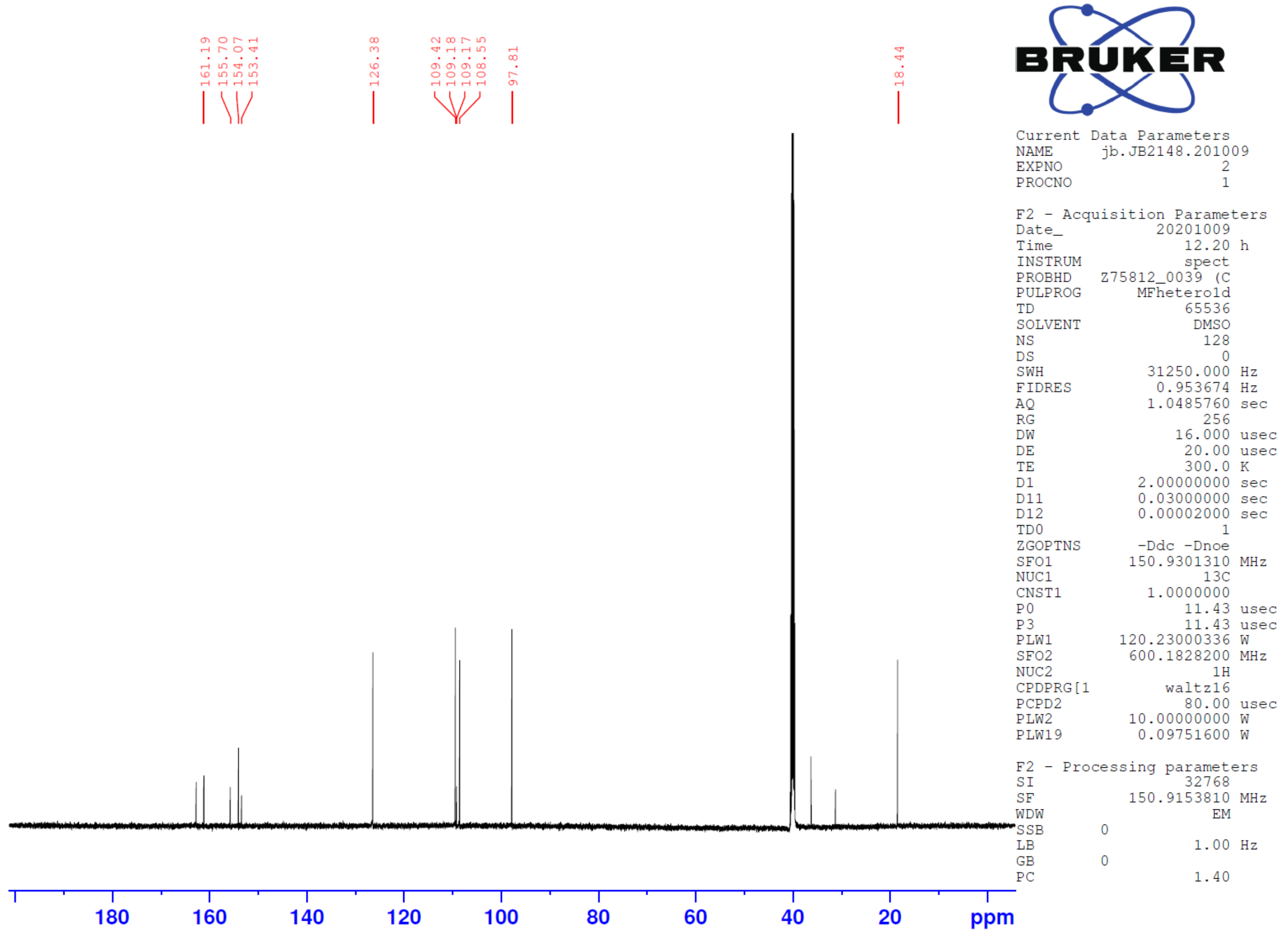
161.19
155.70
154.07
153.41
126.38
109.42
109.18
109.17
108.55
97.81
18.44
180
160
140
120
100
80
60
40
20
ppm
Current Data Parameters
NAME jb.JB2148.201009
EXPNO 2
PROCNO 1
F2 - Acquisition Parameters
Date_ 20201009
Time 12.20 h
INSTRUM spect
PROBHD Z75812_0039 (C
PULPROG MFhetero1d
TD 65536
SOLVENT DMSO
NS 128
DS 0
SWH 31250.000 Hz
FIDRES 0.953674 Hz
AQ 1.0485760 sec
RG 256
DW 16.000 usec
DE 20.00 usec
TE 300.0 K
D1 2.00000000 sec
D11 0.03000000 sec
D12 0.00002000 sec
TD0 1
ZGOPTNS -Ddc -Dnoe
SFO1 150.9301310 MHz
NUC1 13C
CNST1 1.0000000
P0 11.43 usec
P3 11.43 usec
PLW1 120.23000336 W
SFO2 600.1828200 MHz
NUC2 1H
CPDPRG[1 waltz16
PCPD2 80.00 usec
PLW2 10.00000000 W
PLW19 0.09751600 W
F2 - Processing parameters
SI 32768
SF 150.9153810 MHz
WDW EM
SSB 0
LB 1.00 Hz
GB 0
PC 1.40

3.13. *N*,*N*-Dimethyl-7-nitrobenzo[*c*][1,2,5]oxadiazol-4-amine (NBD)

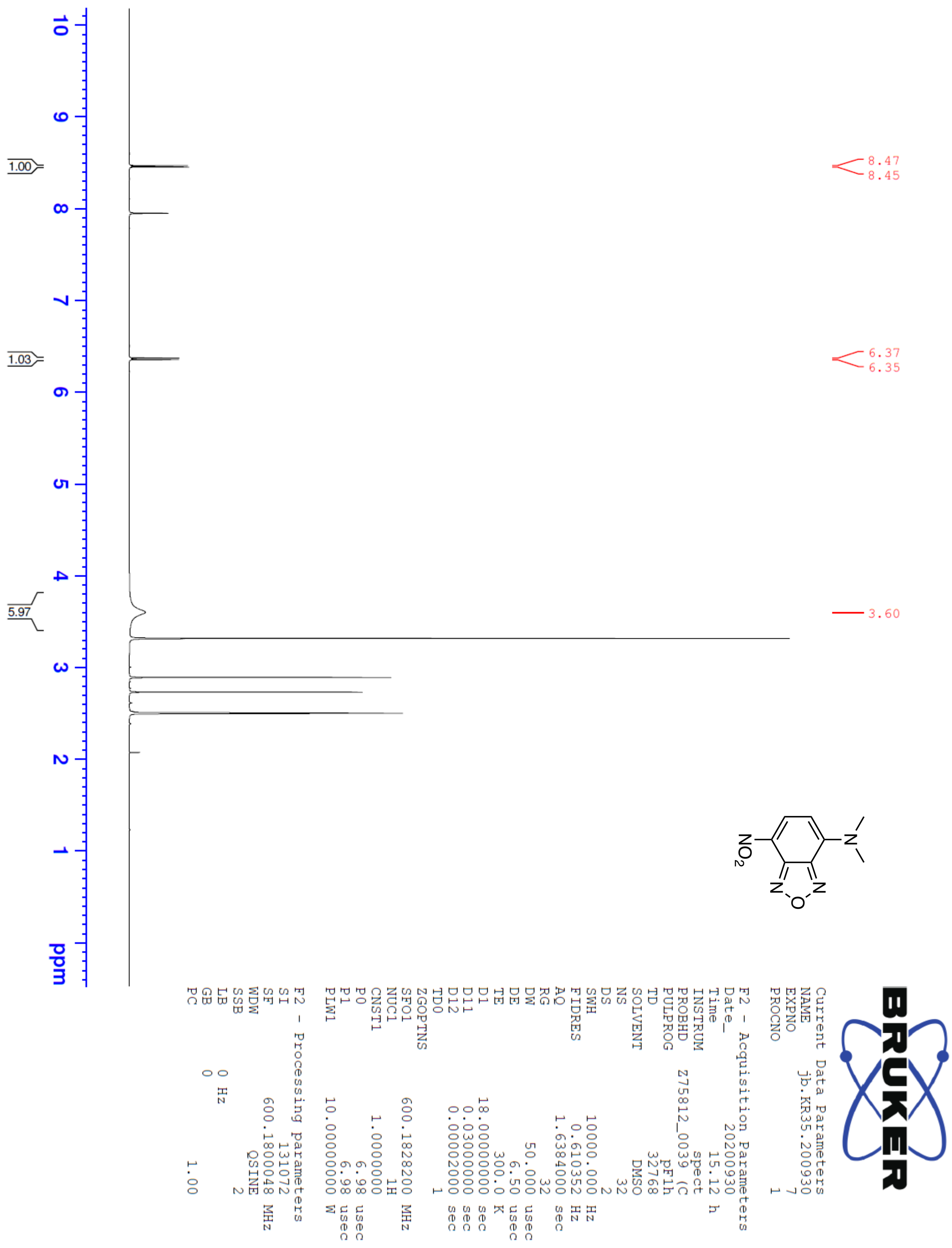

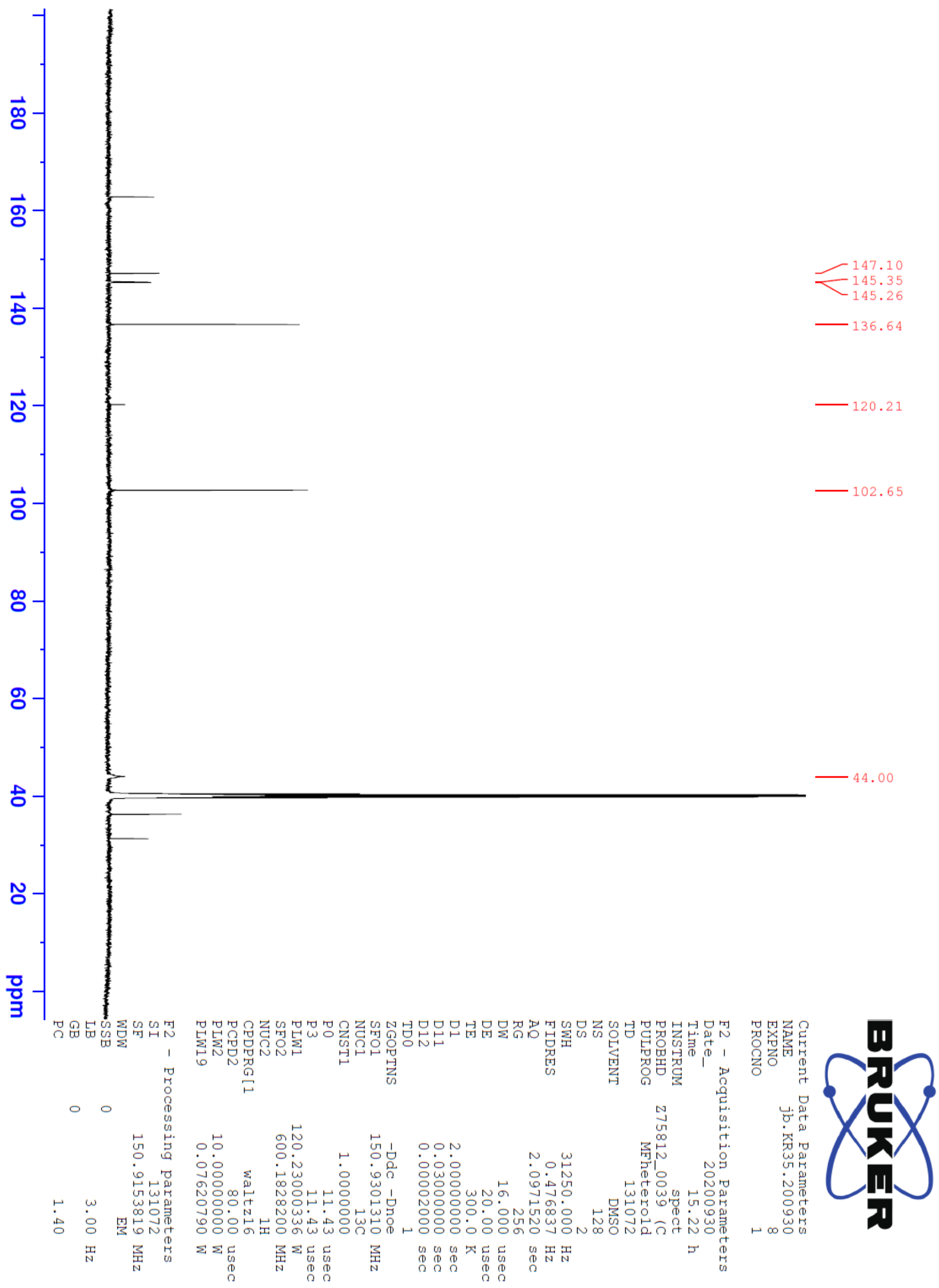
147.10
145.35
145.26
136.64
120.21
102.65
44.00
180
160
140
120
100
80
60
40
20
ppm
BRUKER
Current Data Parameters
NAME jb.KR35.200930
EXPNO 8
PROCNO 1
F2 - Acquisition Parameters
Date_ 20200930
Time 15.22 h
INSTRUM spect
PROBHD Z75812_0039 (C
PULPROG MFhetero1d
TD 131072
SOLVENT DMSO
NS 128
DS 2
SWH 31250.000 Hz
FIDRES 0.476837 Hz
AQ 2.0971520 sec
RG 256
DW 16.000 usec
DE 20.00 usec
TE 300.0 K
D1 2.00000000 sec
D11 0.03000000 sec
D12 0.00002000 sec
TD0 1
ZGOPTNS -Ddc -Dnoe
SFO1 150.9301310 MHz
NUC1 13C
CNST1 1.0000000
P0 11.43 usec
P3 11.43 usec
PLW1 120.23000336 W
SFO2 600.1828200 MHz
NUC2 1H
CPDPRG[1 waltz16
PCPD2 80.00 usec
PLW2 10.00000000 W
PLW19 0.07620790 W
F2 - Processing parameters
SI 131072
SF 150.9153819 MHz
WDW EM
SSB 0
LB 3.00 Hz
GB 0
PC 1.40

3.14. *N*,*N*-Bis(methyl-d3)-7-nitrobenzo[*c*][1,2,5]oxadiazol-4-amine (NBD-d6)

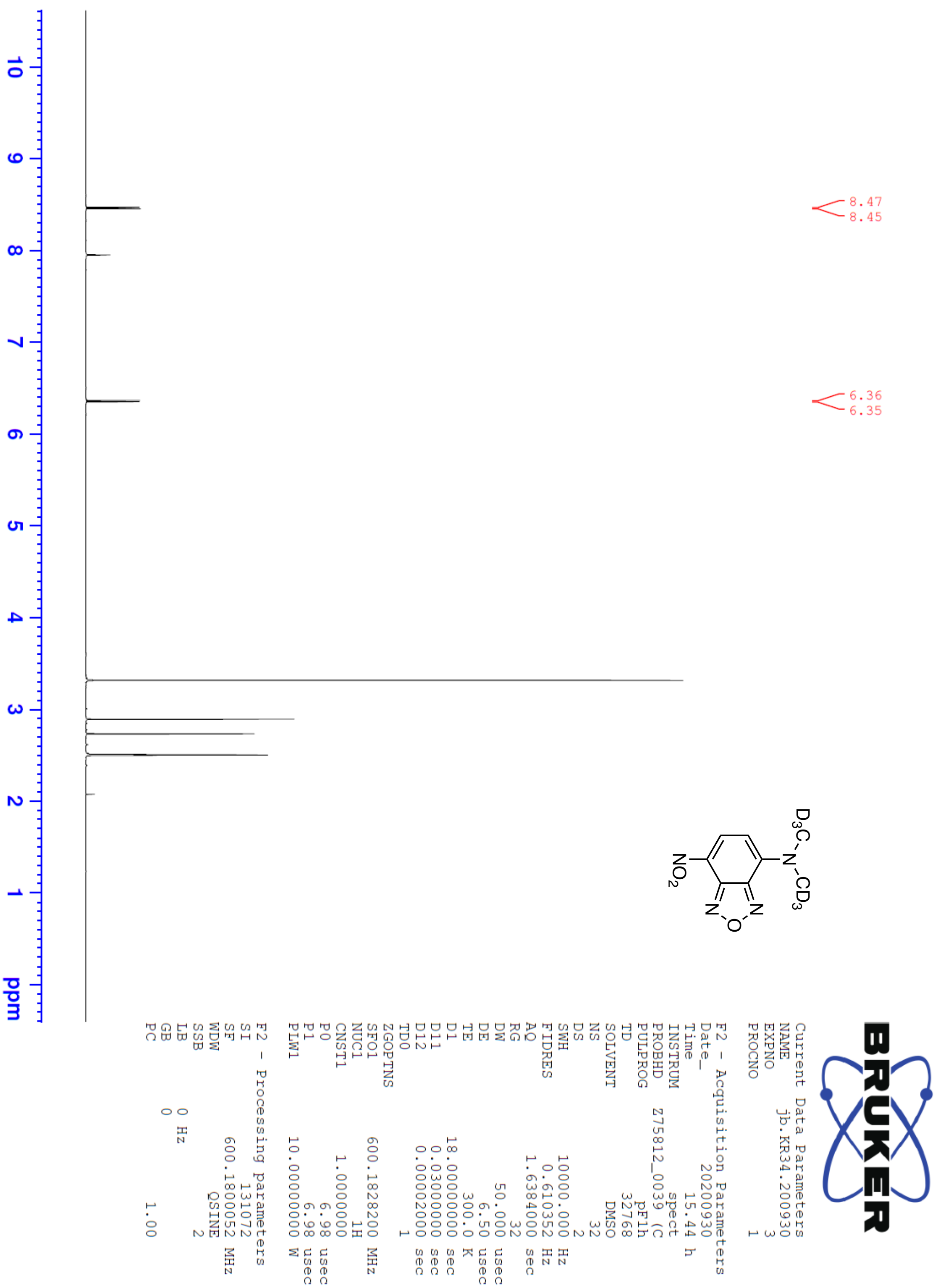

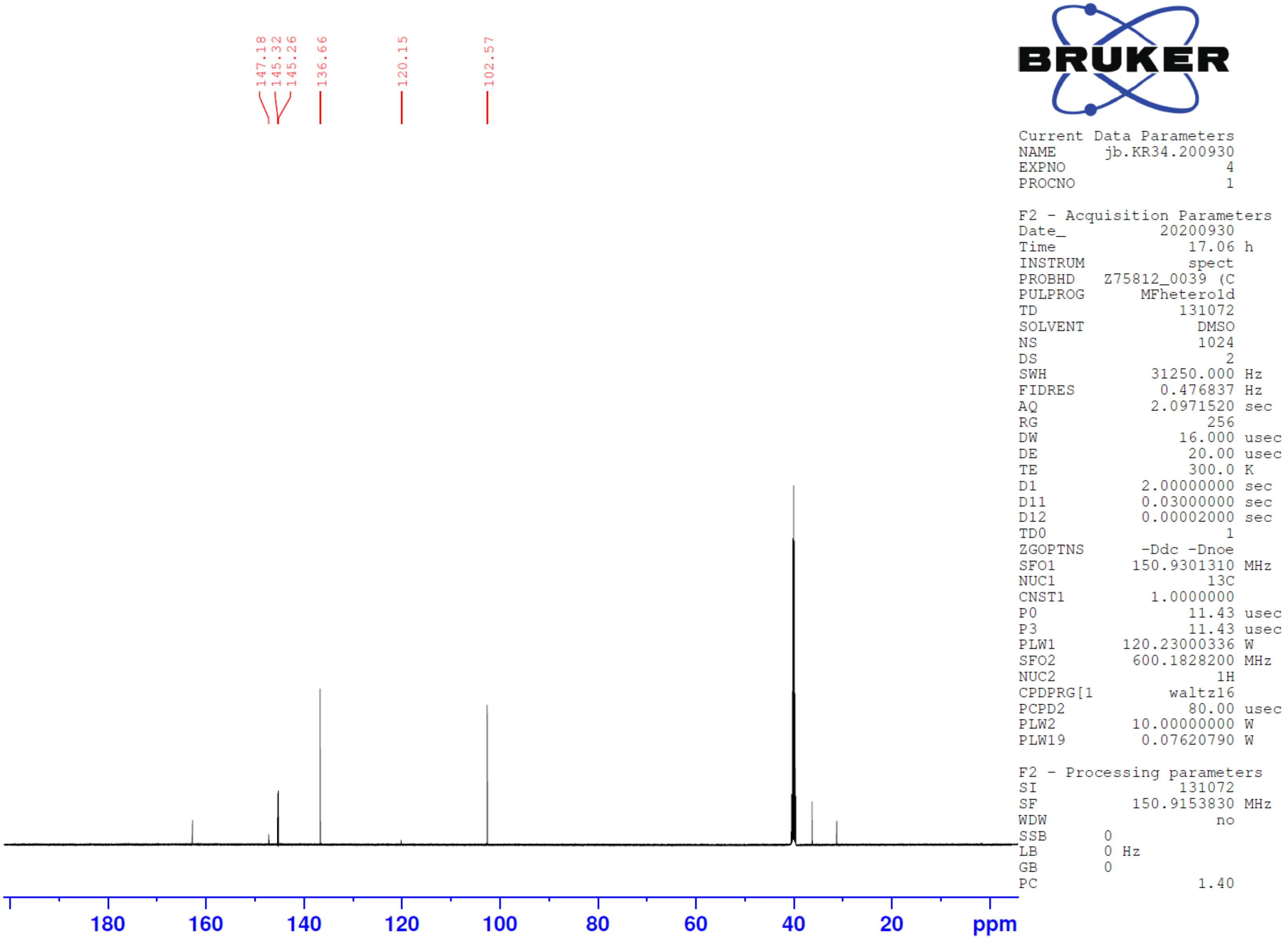
147.18
145.32
145.26
136.66
120.15
102.57
BRUKER
Current Data Parameters
NAME jb.KR34.200930
EXPNO 4
PROCNO 1
F2 - Acquisition Parameters
Date_ 20200930
Time 17.06 h
INSTRUM spect
PROBHD Z75812_0039 (C
PULPROG MFhetero1d
TD 131072
SOLVENT DMSO
NS 1024
DS 2
SWH 31250.000 Hz
FIDRES 0.476837 Hz
AQ 2.0971520 sec
RG 256
DW 16.000 usec
DE 20.00 usec
TE 300.0 K
D1 2.00000000 sec
D11 0.03000000 sec
D12 0.00002000 sec
TD0 1
ZGOPTNS -Ddc -Dnoe
SFO1 150.9301310 MHz
NUC1 13C
CNST1 1.0000000
P0 11.43 usec
P3 11.43 usec
PLW1 120.23000336 W
SFO2 600.1828200 MHz
NUC2 1H
CPDPRG[1 waltz16
PCPD2 80.00 usec
PLW2 10.00000000 W
PLW19 0.07620790 W
F2 - Processing parameters
SI 131072
SF 150.9153830 MHz
WDW no
SSB 0
LB 0 Hz
GB 0
PC 1.40
180
160
140
120
100
80
60
40
20
ppm

3.15. 3,7-Bis(dimethylamino)phenothiazin-5-ium (Methylene Blue)

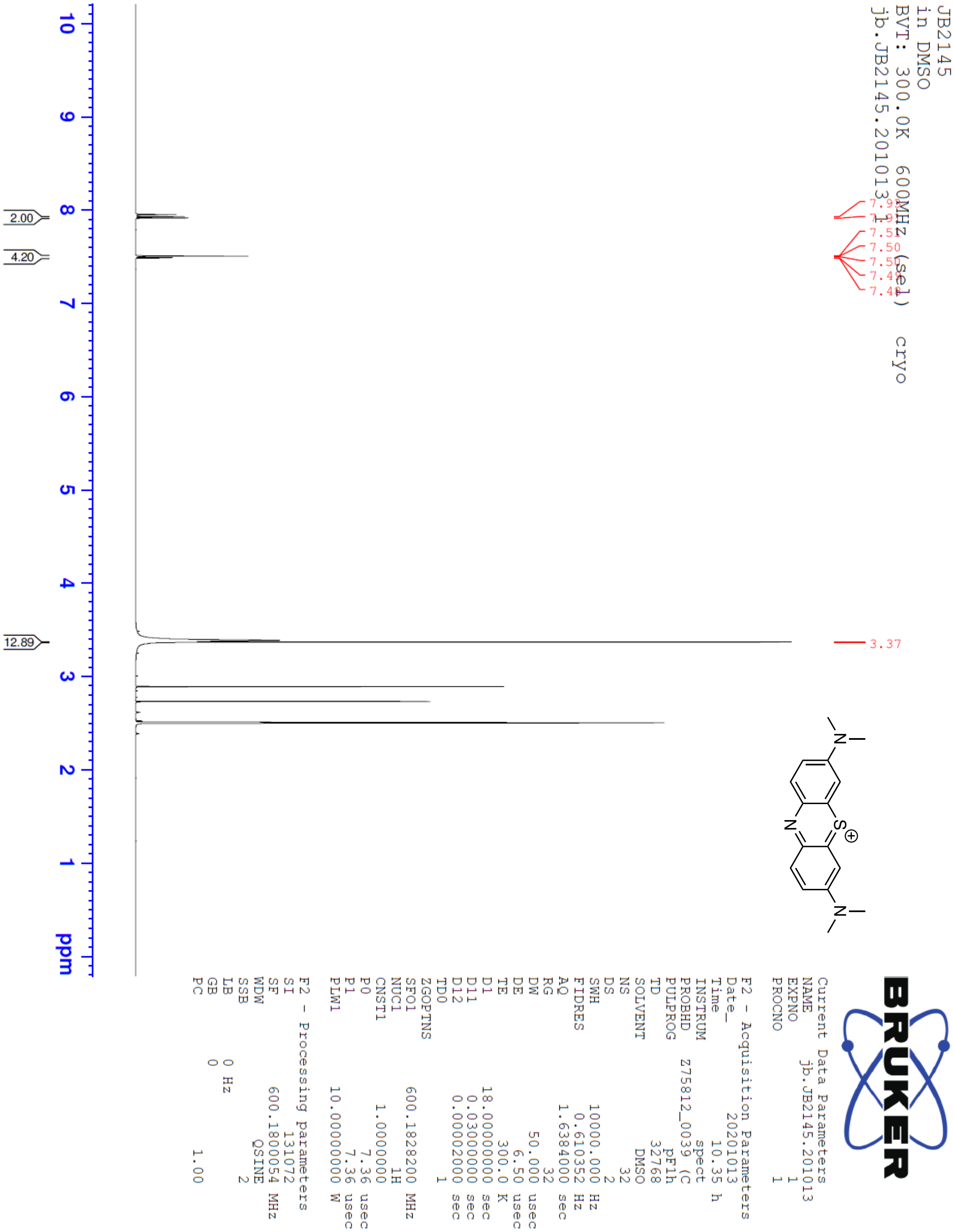

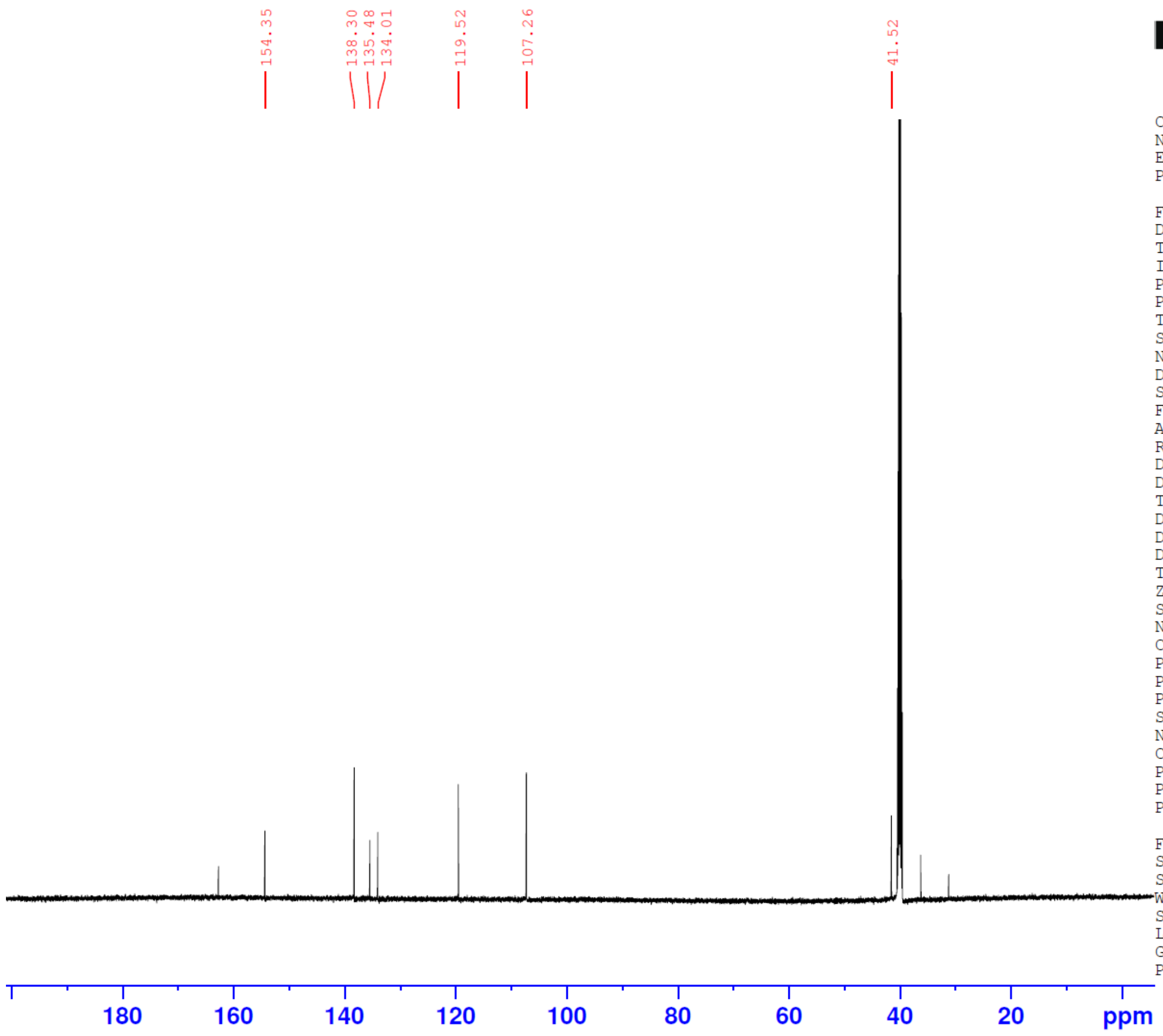

Current Data Parameters		
NAME	jb.JB2145.201013	
EXPNO	2	
PROCNO	1	
F2 - Acquisition Parameters		
Date_	20201013	
Time	10.49	h
INSTRUM	spect	
PROBHD	Z75812_0039 (C	
PULPROG	MFhetero1d	
TD	65536	
SOLVENT	DMSO	
NS	256	
DS	0	
SWH	31250.000	Hz
FIDRES	0.953674	Hz
AQ	1.0485760	sec
RG	256	
DW	16.000	usec
DE	20.00	usec
TE	300.0	K
D1	2.00000000	sec
D11	0.03000000	sec
D12	0.00002000	sec
TD0	1	
ZGOPTNS	-Ddc -Dnoe	
SFO1	150.9301310	MHz
NUC1	13C	
CNST1	1.0000000	
P0	11.43	usec
P3	11.43	usec
PLW1	120.23000336	W
SFO2	600.1828200	MHz
NUC2	1H	
CPDPRG[1	waltz16	
PCPD2	80.00	usec
PLW2	10.00000000	W
PLW19	0.09751600	W
F2 - Processing parameters		
SI	32768	
SF	150.9153833	MHz
WDW	EM	
SSB	0	
LB	1.00	Hz
GB	0	
PC	1.40	

3.16. 3,7-Bis(bis(methyl-d3)amino)phenothiazin-5-ium (Methylene Blue-d12)

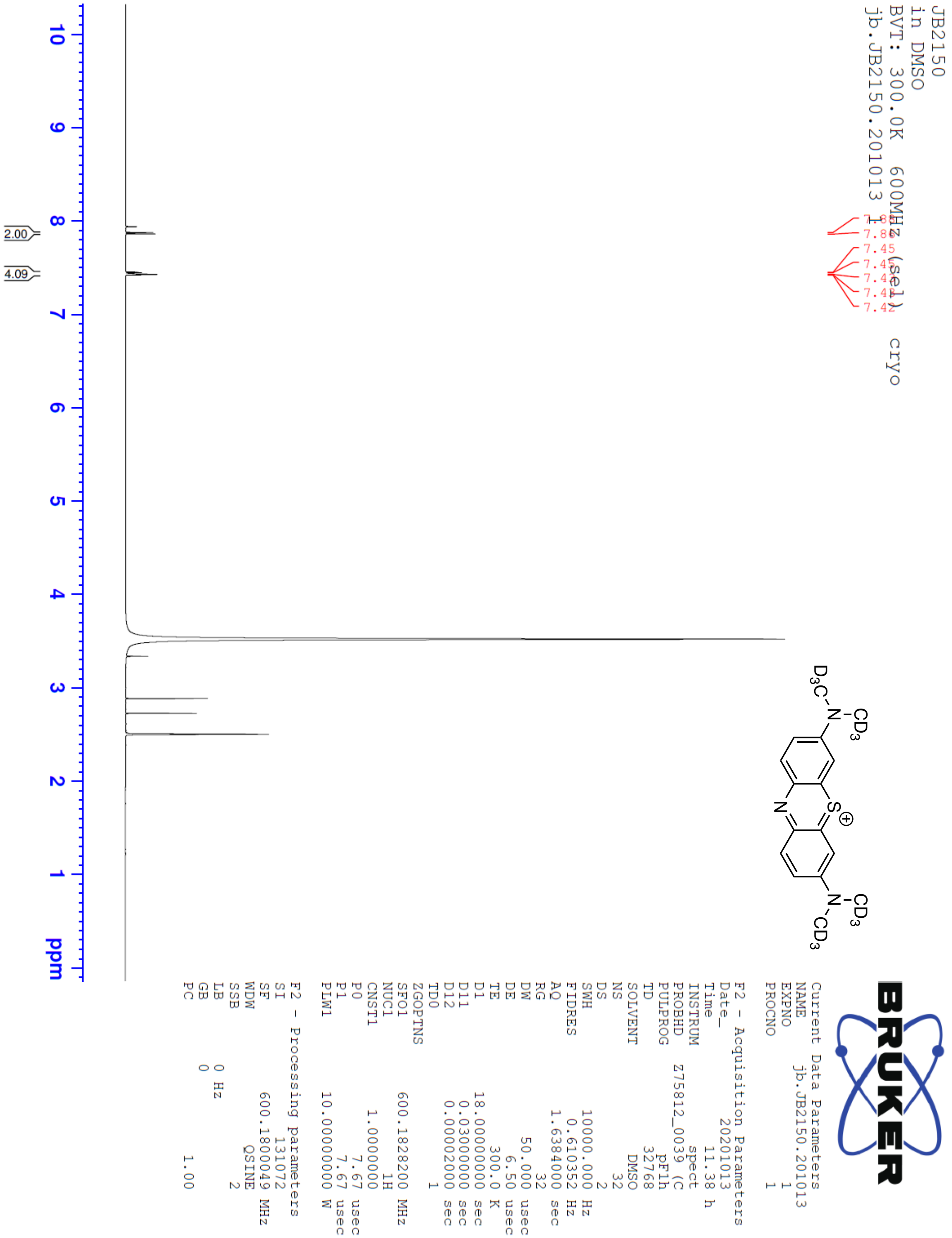

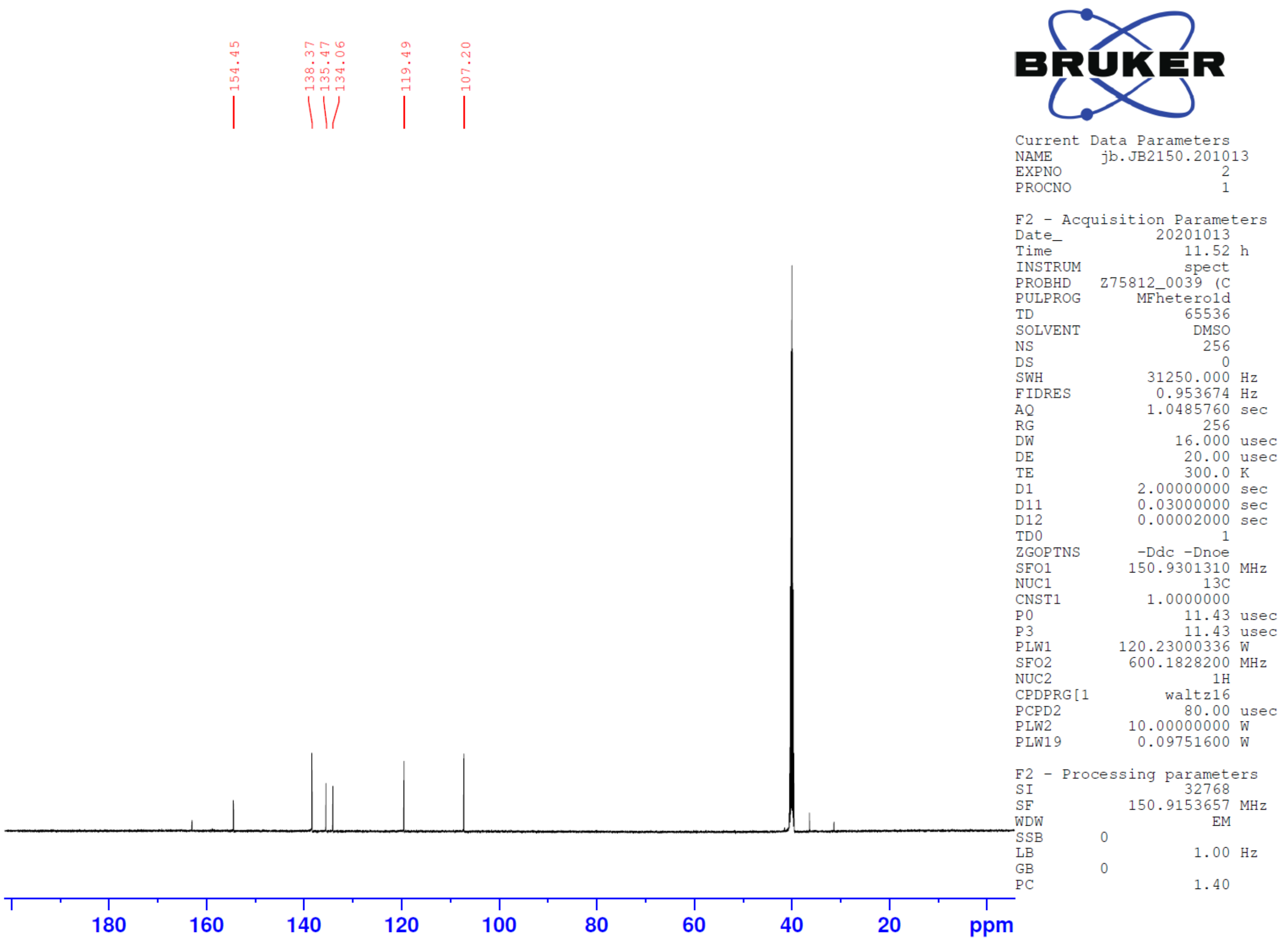
154.45
138.37
135.47
134.06
119.49
107.20
180
160
140
120
100
80
60
40
20
ppm
Current Data Parameters
NAME jb.JB2150.201013
EXPNO 2
PROCNO 1
F2 - Acquisition Parameters
Date_ 20201013
Time 11.52 h
INSTRUM spect
PROBHD Z75812_0039 (C
PULPROG MFhetero1d
TD 65536
SOLVENT DMSO
NS 256
DS 0
SWH 31250.000 Hz
FIDRES 0.953674 Hz
AQ 1.0485760 sec
RG 256
DW 16.000 usec
DE 20.00 usec
TE 300.0 K
D1 2.00000000 sec
D11 0.03000000 sec
D12 0.00002000 sec
TD0 1
ZGOPTNS -Ddc -Dnoe
SFO1 150.9301310 MHz
NUC1 13C
CNST1 1.0000000
P0 11.43 usec
P3 11.43 usec
PLW1 120.23000336 W
SFO2 600.1828200 MHz
NUC2 1H
CPDPRG[1 waltz16
PCPD2 80.00 usec
PLW2 10.00000000 W
PLW19 0.09751600 W
F2 - Processing parameters
SI 32768
SF 150.9153657 MHz
WDW EM
SSB 0
LB 1.00 Hz
GB 0
PC 1.40

4. SNAP$_f$ construct

SNAP$_f$ sequence:

```
MASWSHPQFE KGADDDDKVP HMDKDCEMKR TTLDSPLGKL ELSGCEQGLH RIIFLGKGTS
AADAVEVPAP AAVLGGPEPL MQATAWLNAY FHQPEAIEEF PVPALHHPVF QQESFTRQVL
WKLLKVVKFG EVISYSHLAA LAGNPAATAA VKTALSGNPV PILIPCHRVV QGDLDVGGYE
GGLAVKEWLL AHEGHRLGKP GLGAPGFSSI SAHHHHHHHHHH
```

Strep-Tag II, Enterokinase-site, SNAP$_f$, His-Tag

5. SNAP–Halo construct and mass spectrometry

SNAP–Halo sequence:

```
MASWSHPQFE KGADDDDKVP HMDKDCEMKR TTLDSPLGKL ELSGCEQGLH EIIFLGKGTS
AADAVEVPAP AAVLGGPEPL MQATAWLNAY FHQPEAIEEF PVPALHHPVF QQESFTRQVL
WKLLKVVKFG EVISYSHLAA LAGNPAATAA VKTALSGNPV PILIPCHRVV QGDLDVGGYE
GGLAVKEWLL AHEGHRLGKP GLGGRLEVLF QGPKAFLEGS EIGTGFPFDP HYVEVLGERM
HYVDVGPRDG TPVLFLHGNP TSSYVWRNII PHVAPTHRCI APDLIGMGKS DKPDLGYFFD
DHVRFMDAFI EALGLEEVVL VIHDWGSALG FHWAKRNPER VKGIAFMEFI RPIPTWDEWP
EFARETFQAF RTTDVGRKLI IDQNVFIEGT LPMGVVRPLT EVEMDHYREP FLNPVDREPL
WRFPNELPIA GEPANIVALV EEYMDWLHQS PVPKLLFWGT PGVLIPPAEA ARLAKSLPNC
KAVDIGPGLN LLQEDNPDLI GSEIARWLST LEISGAPGFS SISAHHHHHH HHHH*
```

Strep-Tag II, Enterokinase-site, SNAP, Precission Sequence, Halo, His-Tag

Condition	calc.	found
SNAP–Halo	59072	59069
SNAP–Halo:BG-TMR+CA-SiR	60244	60245
SNAP–Halo:BG-SiR+CA-TMR	60244	60241
SNAP–Halo:BG-TMR-d12+CA-SiR-d12	60268	60269
SNAP–Halo:BG-SiR-d12+CA-TMR-d12	60268	60267
SNAP–Halo:BG-TMR	59603	59602
SNAP–Halo:BG-SiR	59645	59642
SNAP–Halo:CA-TMR	59671	59669
SNAP–Halo:CA-SiR	59713	59711
SNAP–Halo:BG-TMR-d12	59612	59615
SNAP–Halo:BG-SiR-d12	59657	59656
SNAP–Halo:CA-TMR-d12	59683	59682
SNAP–Halo:CA-SiR-d12	59725	59724

6. Supplementary Figures

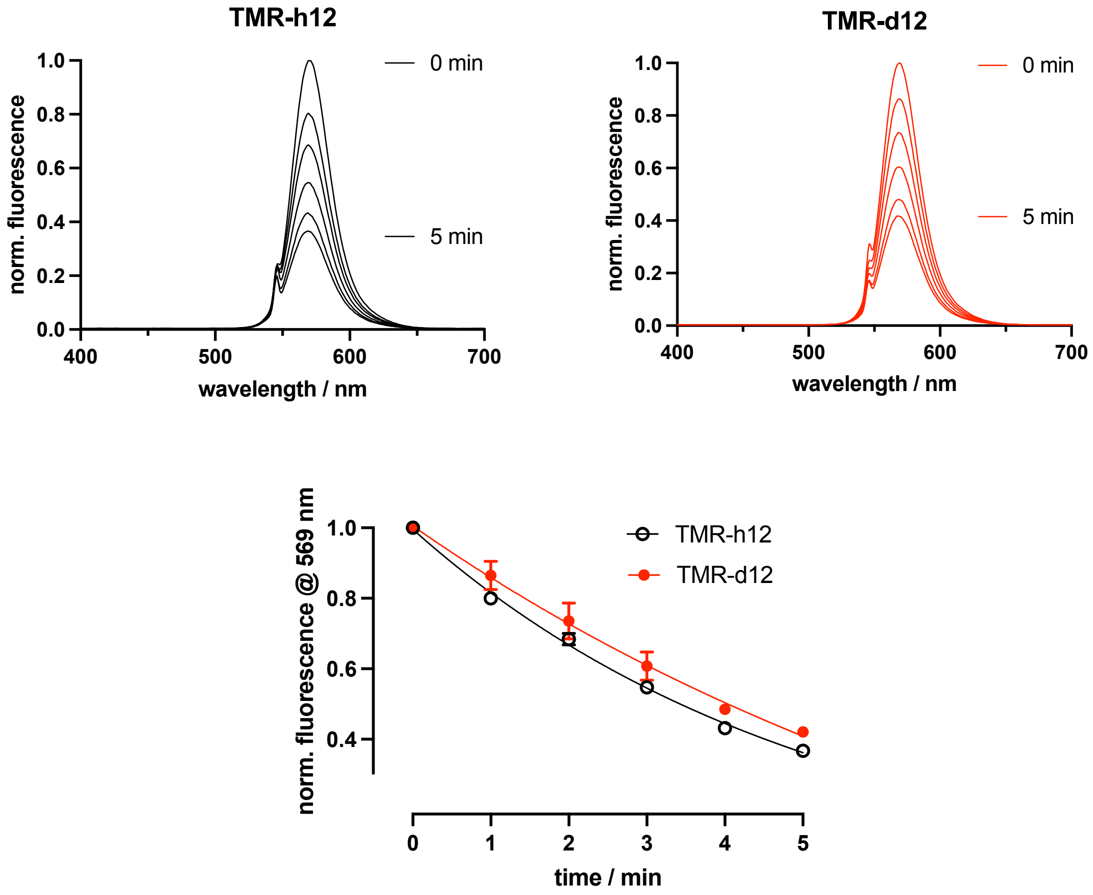

Supporting Figure 1: In vitro bleaching of TMR and TMR-d12 with white light. Following fluorescence emission after 0, 1, 2, 3, 4, and 5 minutes of irradiation (top). Normalized fluorescence at 569 nm plotted over time (bottom). Mean±S.D.

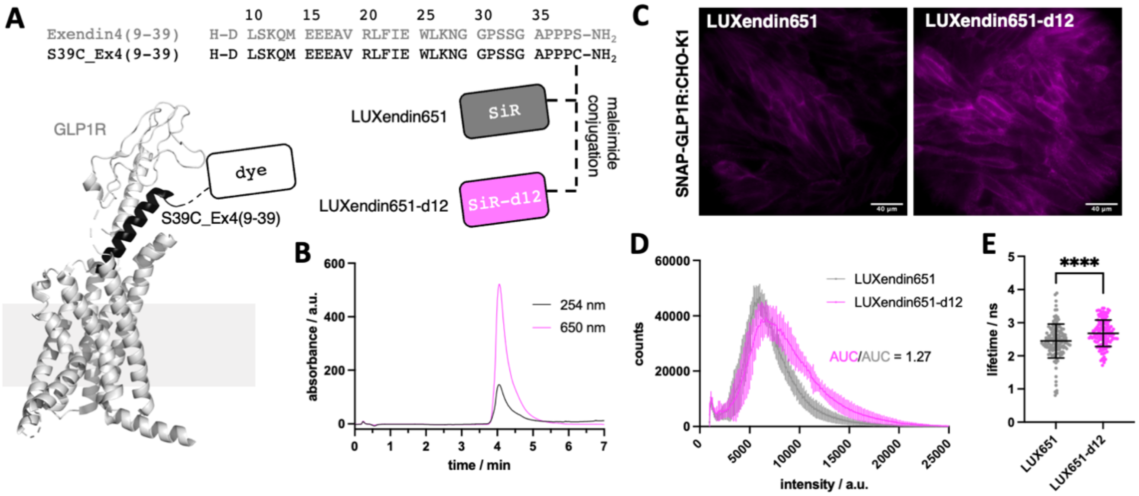

Supporting Figure 2: LUXendin651 versus LUXendin651-d12 in fixed SNAP-GLP1R:CHO-K1 cells. A) S39C_Ex4(9-39) is derived from Exendin4(9-39) with a C-terminal cysteine handle for maleimide bioconjugation wit SiR or SiR-d12, yielding LUXendin651 and LUXendin651-d12, respectively, which are high affinity and selective antagonists towards the GLP1R. B) LCMS trace of LUXendin651-d12. C) Labelling of SNAP-GLP1R in fixed CHO-K1 cells with n = 2 biological replicates. Scale bar = 40 µm. D) Pooled raw histograms from five images as represented in (C) show increased brightness of LUXendin651-d12 versus LUXendin651. E) Fluorescence lifetimes of LUXendin651-d12 versus LUXendin651 in fixed SNAP-GLP1R:CHO-K1 cells. n>200 ROIs, **** indicates statistical significance (unpaired t-test, p<E-26). Mean±SD.

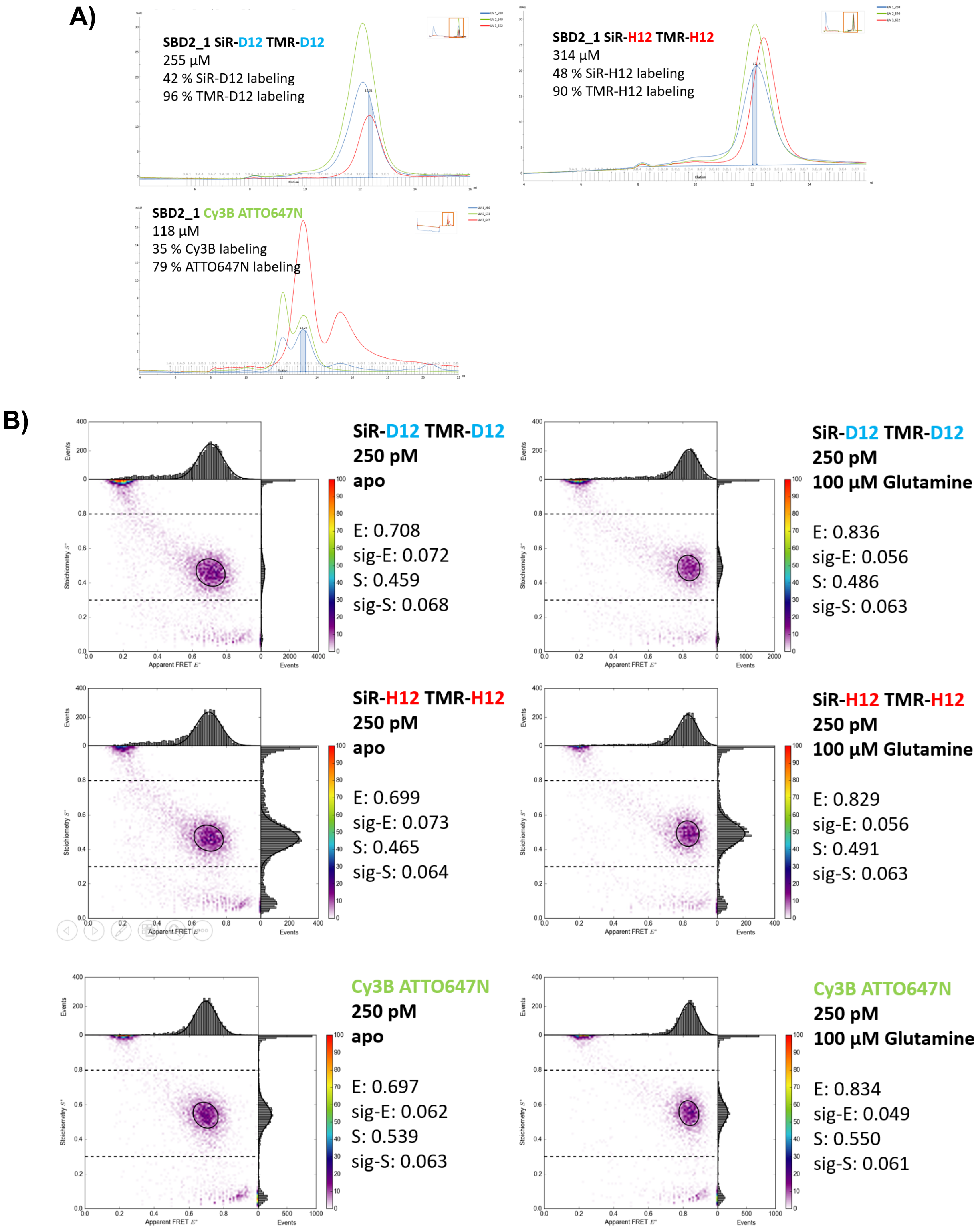

Supporting Figure 3: Summary of SEC labelling characterization (A) and smFRET data for different fluorophore pairs (B) for stochastic labelling of SBD2.

7. Supplementary Schemes

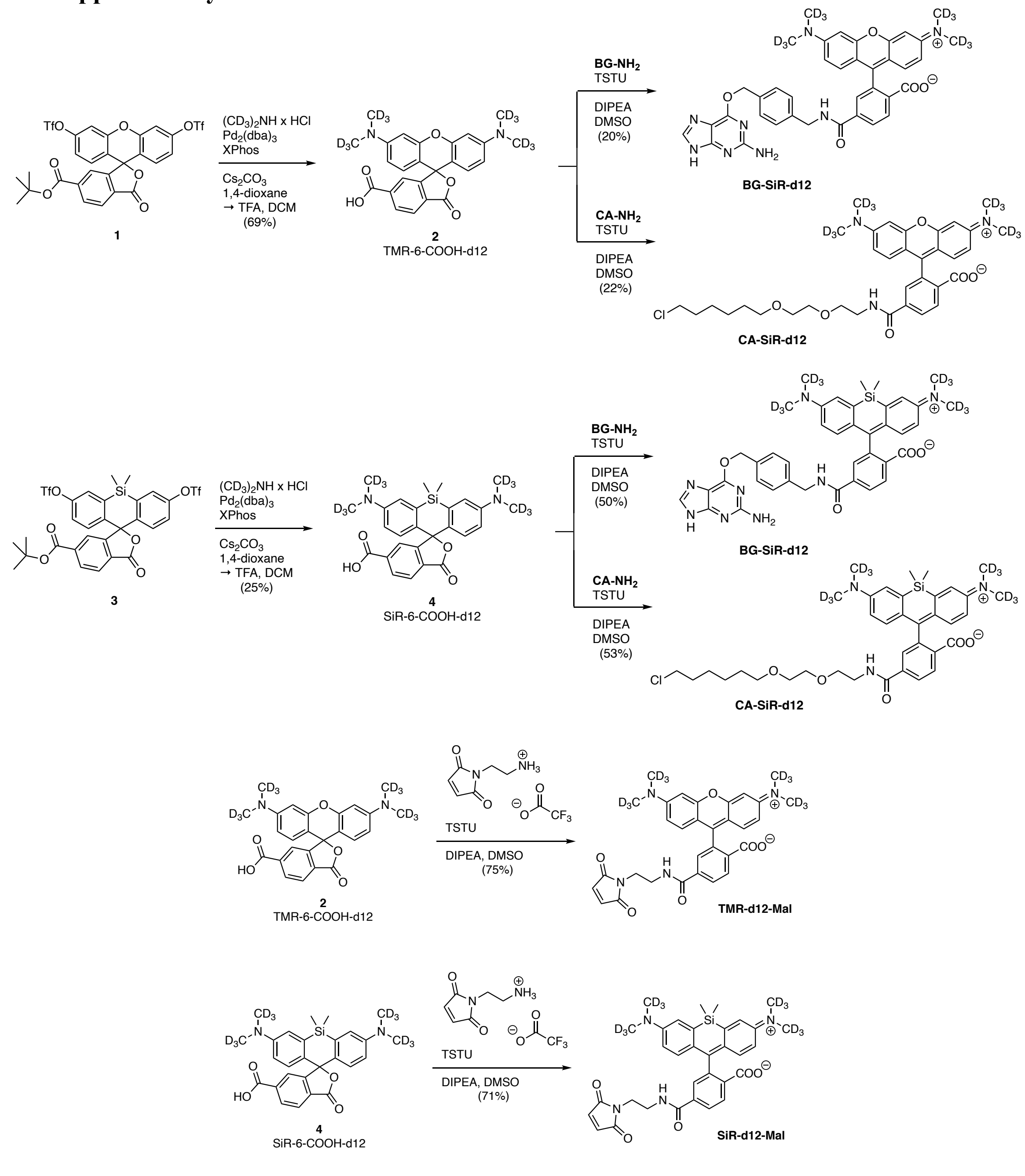

Supporting Scheme 1: Chemical synthesis of rhodamine d12 probes.

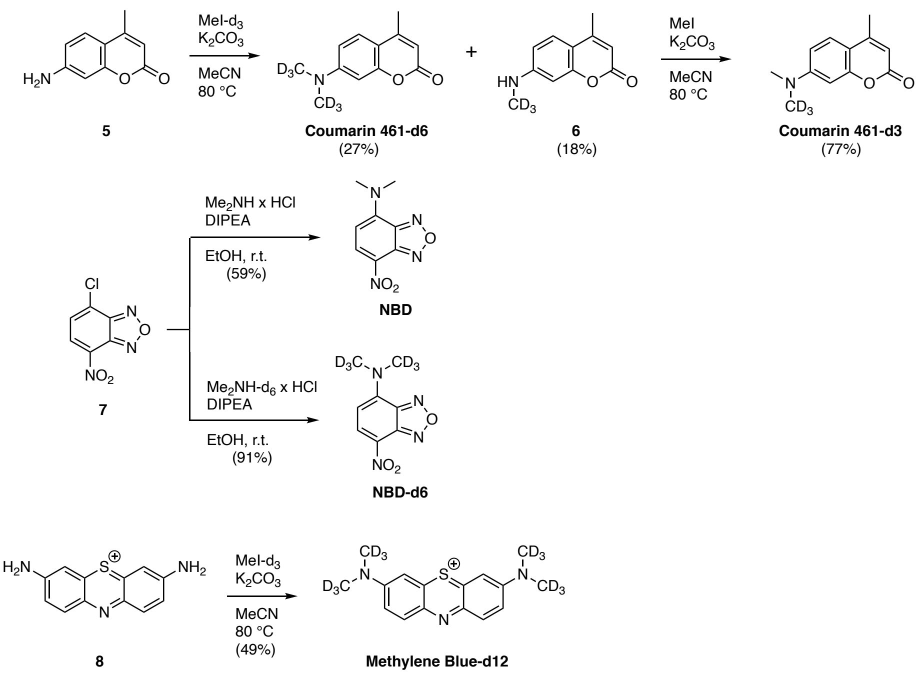

Supporting Scheme 2: Chemical synthesis of (deuterated) coumarin, NBD and methylene blue.

8. Supplementary Tables

Table S1: Photophysical parameters from Figure 1 with error margins.

	TMR	TMR-d12	SiR	SiR-d12
Ext. coefficient [M^{-1} cm^{-1}]	78,000±200	90,000±600	141,000±1,000	156,000±1,600
Quantum yield (integrating sphere) [%]	43.1±5.2	51.3±5.0	35.3±4.8	46.1±3.0
Lifetime [ns]	2.287±0.392	2.588±0.495	2.898±0.383	3.534±0.164

Table S2: Photophysical parameters for LUXendin651(-d12).

	LUXendin651	LUXendin651-d12
Lifetime [ns]	2.448±0.513	2.682±0.400

Table S3: Photophysical parameters from Figure 4 with error margins.

	Coumarin461<br><br>Coumarin461-d6	NBD<br><br>NBD-d6	Methylene blue<br><br>Methylene blue-d12
Ext. coefficient [M^{-1} cm^{-1}]	28,100±400<br><br>27,900±2,000	16,300±700<br><br>16,000±1,000	45,500±2,500<br><br>49,800±1,800
Quantum yield (platreader) [%]	19±0.2<br><br>27±2.4	55±7.0<br><br>59±1.8	1.0±0.05<br><br>1.3±0.02

Table S4: Temperature-dependent lifetimes from Figure 5.

Lifetime [ns]	Halo:SiR	Halo:SiR-d12	SNAP:SiR	SNAP:SiR-d12
20 °C	3.53±0.05	4.02±0.04	3.26±0.27	3.75±0.28
30 °C	3.39±0.04	3.89±0.03	3.03±0.02	3.51±0.05
40 °C	3.16±0.04	3.68±0.03	2.84±0.02	3.37±0.03

Table S5: Normalized values from Table S3.

Lifetime [%]	Halo:SiR	Halo:SiR-d12	SNAP:SiR	SNAP:SiR-d12
20 °C	100	100	100	100
30 °C	96.2±0.7	96.8±0.5	93.5±7.0	93.9±5.3
40 °C	89.6±0.2	91.5±0.6	87.8±7.5	90.3±6.7

9. References

1. Farrants, H. *et al.* SNAP-Tagged Nanobodies Enable Reversible Optical Control of a G Protein-Coupled Receptor *via* a Remotely Tethered Photoswitchable Ligand. *ACS Chem. Biol.* **13**, 2682–2688 (2018).
2. Grimm, J. B. *et al.* A general method to improve fluorophores for live-cell and single-molecule microscopy. *Nat. Methods* **12**, 244–250 (2015).
3. Ast, J. *et al.* Super-resolution microscopy compatible fluorescent probes reveal endogenous glucagon-like peptide-1 receptor distribution and dynamics. *Nat. Commun.* **11**, 467 (2020).
